# TENT5C extends *Odf1* poly(A) tail to sustain sperm morphogenesis and fertility

**DOI:** 10.1038/s41467-026-71953-4

**Published:** 2026-04-20

**Authors:** Marine Baptissart, Ankit Gupta, Maira L. Perez, Alexander C. Poirot, Brian N. Papas, Carlos M. Guardia, Marcos Morgan

**Affiliations:** 1 Male Reproduction and RNA Biology Group, Reproductive and Developmental Biology Laboratory, National Institute of Environmental Health Sciences, National Institutes of Health, Durham, NC USA; 2 Placental Cell Biology Group, Reproductive and Developmental Biology Laboratory, National Institute of Environmental Health Sciences, National Institutes of Health, Durham, NC USA; 3 Integrative Bioinformatics, Biostatistics and Computational Biology Branch, National Institute of Environmental Health Sciences, National Institutes of Health, Durham, NC USA

**Keywords:** Spermatogenesis, RNA modification

## Abstract

Changes in the poly(A) tail length of *Odf1* and other transcripts critical for male fertility have been linked to translational activation during sperm formation^1–3^. The mRNA poly(A) polymerase TENT5C is required for fastening the flagellum to the sperm head, but its role in shaping the poly(A) tail profile of the spermatid transcriptome remains unclear ^4,5^. Here, we comprehensively document how changes in mRNA poly(A) tail length across the transcriptome reflect transcript metabolism in spermatids. In the absence of TENT5C polymerase activity, *Odf1* transcripts show shorter poly(A) tails and, together with ODF1 protein, fail to accumulate at the spermatid neck. Mice expressing a catalytically inactive TENT5C produce headless spermatozoa with flagellar abnormalities associated with ODF1 deficiency ^6^. We propose that TENT5C poly(A) polymerase activity regulates the stability and local translation of *Odf1* mRNAs at the neck of late-stage spermatids, a process critical for sperm morphogenesis and fertility. These findings highlight the power of poly(A) tail profiling to identify abnormal mRNA processing causative of infertility.

## Abstract (web summary)

Here they show that mRNA poly(A) tail elongation by TENT5C promotes ODF1 accumulation at the sperm neck to prevent sperm decapitation. These findings highlight poly(A) tail profiling as a powerful approach for identifying male infertility factors.

## Introduction

Spermiogenesis is the final step of spermatogenesis, where haploid spermatids exchange histones for protamines and elongate to produce sperm with a condensed nucleus and a flagellum. Spermiogenesis is one of the rare events where post-transcriptional regulation occurs in the absence of transcription^7^. Transcription ceases in step-10 elongating spermatids and remains silent for the rest of the differentiation process^8,9^. In this context, the mRNAs required to orchestrate the late differentiation program are synthesized early on and stabilized before translational activation^1–3,10–12^. However, the post-transcriptional mechanisms controlling the temporal disconnect between mRNA transcription and translation during spermiogenesis are poorly understood.

The poly(A) tail is a stretch of A nucleotides (nt) added co-transcriptionally to the 3′-end of most eukaryotic mRNAs^13^. Once in the cytoplasm, poly(A)-binding proteins (PABPs) coat the poly(A) tail to favor transcript stability and translation^14^. Deadenylase complexes progressively peel off PABPs, leading to poly(A) tail shortening and mRNA decay^15^. A correlation between poly(A) tail length and mRNA stability and translation has been clearly established in cells where transcription is silenced^3,16–18^. The most well-characterized example is the elongation of poly(A) tails during oocyte activation, which leads to translational activation^19–22^. Early reports have shown that during the transition from pachytene spermatocytes to round spermatids (RS), the poly(A) tail of transcripts critical for spermiogenesis is extended by up to 100 additional nt, a process proposed to favor their stability^10–12^. As the RS differentiate into elongated spermatids (ES), mRNAs encoding for key structural and chromatin factors show poly(A) tail shortening from ~150- to ~30-nt long, a dynamic associated with their translational activation^1–3^. A recent study examined transcriptome-wide poly(A) tail dynamics during spermiogenesis using long-read sequencing^23^. However, unlike previous reports, transcripts were proposed to be stored and translationally repressed with tails ~30-nt long. Thus, how the global poly(A) tail dynamics regulate mRNA metabolism and spermatid differentiation is still unclear.

In recent years, several terminal nucleotidyltransferases (TENTs) have been shown to be required to sustain gametogenesis^4,24–26^. Among them, the poly(A) polymerase TENT5C, highly abundant in the manchette of ES, was shown to be required for male fertility^4^. *Tent5c-*null males produce headless spermatozoa due to defective spermiation, the process by which the ES are released from the seminiferous tubules. This phenotype was not associated with global changes in mRNA accumulation when sampling whole testes, suggesting that the poly(A) polymerase activity of TENT5C might not be required for spermatogenesis^4^. More recently, a poly(A) profile of whole testes from *Tent5c-*null animals has shown changes in poly(A) tail length for several transcripts expressed in various testicular cell types^5^. Thus, the role of TENT5C poly(A) polymerase, if any, in late spermatid differentiation, where the phenotype is observed remains unclear.

In this study, we characterize transcriptome-wide poly(A) tail dynamics during spermiogenesis and their impact on mRNA metabolism and fertility. After meiosis, most of the RS transcriptome is stabilized with tails ~150-nt long. As the spermatids elongate, the translational activity of mRNAs is reflected by the accumulation of transcripts with ~60-nt long poly(A) tails. Additionally, we show that the poly(A) polymerase activity of TENT5C is required for fertility and spermiogenesis; male mice expressing catalytically inactive TENT5C (*Tent5c*^*dcat/dcat*^) are sterile and produce headless spermatozoa with abnormal flagellar morphologies. We identify outer dense fiber of sperm tails 1 (*Odf1)* as one of the few transcripts dependent on the poly(A) polymerase activity of TENT5C in ES. In *Tent5c*^*dcat/dcat*^ mice, *Odf1* shows a reduction in poly(A) tail length with an increased proportion of transcripts with tails ~60-nt long. This profile is accompanied by loss of *Odf1* mRNA granules and reduced ODF1 protein accumulation at the neck of step-16 spermatids. We propose that TENT5C poly(A) polymerase activity promotes the local stability and translation of *Odf1* mRNAs during late spermiogenesis to secure head–tail fastening, thereby supporting sperm integrity and male fertility. These results show that poly(A) profiling of germ cells can be used to identify specific transcripts that, when dysregulated, cause male infertility.

## Results

### 150-nt poly(A) tails stabilize the spermatid transcriptome

Seminal studies have shown that transcripts required for sperm metabolism, nuclear compaction, and flagellum formation show dynamic poly(A) tail length profiles during spermiogenesis^1–3,10–12^. To understand whether this behavior is characteristic of a few transcripts or prevalent in the spermatid transcriptomes, we used a well-established FACS sorting strategy to isolate germ cell populations enriched with pachytene and diplotene spermatocytes (P/D spermatocytes), RS or ES (Supplementary Fig. 1a)^27^. The purity of isolated populations was quantitatively determined by microscopy using fluorescent markers (Supplementary Fig. 1a–c). Late spermatocytes were identified with the meiotic marker synaptonemal complex protein 3 (SYCP3), while acrosomal structures characteristic of RS and ES were stained with Peanut agglutinin (PNA). DAPI was used to stain DNA. About 50% of the cells from the sorting gate II were P/D spermatocytes, while the gates IIIa and IIIb were enriched with more than 80% of RS and ES, respectively (Supplementary Fig. 1b, c). Cell identity was further confirmed through direct RNA sequencing. Principal component analysis showed clustering of the populations according to their expected cell types (Supplementary Fig. 1d). We next mapped each sequenced transcriptome to cell clusters from a whole testis single-cell RNA sequencing dataset to computationally predict cell type composition^28^. Each individual sample displayed a strong signature corresponding to the anticipated germ cell transcriptome (Supplementary Fig. 1e).

**Fig. 1:**
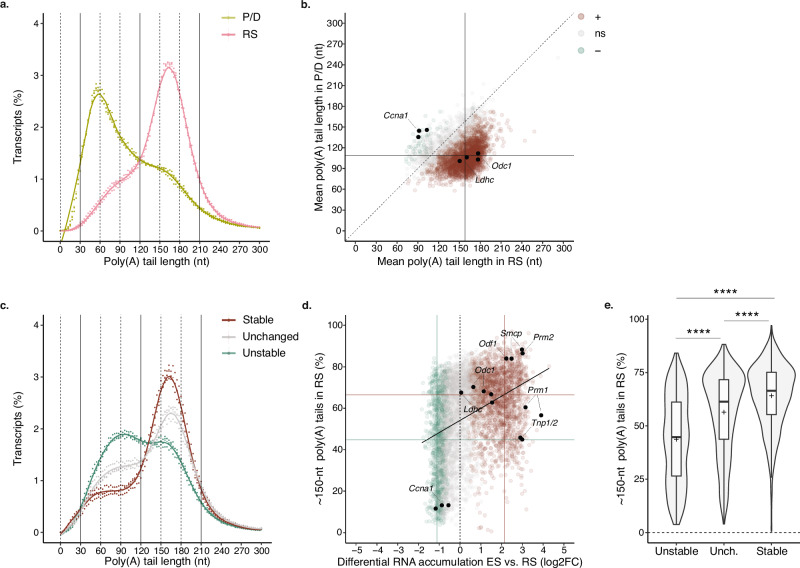
The characteristic accumulation of ~150-nt long poly(A) tails in spermatids is associated with the stabilization of the transcriptome. **a** Poly(A) tail length density plot of pachytene/diplotene spermatocyte (P/D, green) and round spermatid (RS, pink) transcriptomes. Dots indicate values for individual biological replicates. The bars indicate the relative mean percentage of transcripts for each poly(A) tail length. The local polynomial regression fitting is shown as a solid line for each condition. nt nucleotide. **b** Scatter plot comparing the mean poly(A) tail length of each transcript in pachytene/diplotene spermatocytes (P/D) and round spermatids (RS). Each dot represents an individual transcript. Transcripts significantly increasing (+) or decreasing (−) in poly(A) tail length during the transition are shown in brown or green, respectively; Student’s *t*-test, two-tailed, significance threshold *p* < 0.05. ns non-significant. *Odc1*, *Ldhc, and Ccna1* transcripts are indicated in black. **c** Poly(A) tail length density plot as in (**a**) for stable, unchanged, and unstable transcripts in round spermatids (RS) shown in brown, grey, and green, respectively. Transcript stability through spermiogenesis is defined by relative changes in mRNA accumulation between elongated spermatids (ES) and RS. **d** Scatter plot comparing fold change in relative transcript accumulation between round spermatids (RS) and elongated spermatids (ES) to the percentage of reads per transcript with poly(A) tail ~150-nt-long in RS. Each dot represents an individual transcript. Stable, unchanged, and unstable transcripts are indicated in brown, grey, and green, respectively. Solid lines indicate the medians of both variables for stable and unstable mRNAs in brown and green, respectively. The linear fit is shown in black. Specific transcripts are indicated in black. **e** Violin plot showing the proportion of reads per transcript with poly(A) tails ~150-nt long in round spermatids (RS). Transcripts are grouped by stability (Unch.; unchanged). The width of violins shows the density of individual transcripts. The overlaid box plots display means as crosses and medians as lines; the boxes indicate the first and third quartiles and the bars indicate the 10th and 90th percentiles. Kruskal–Wallis test using Bonferroni correction for multiple testing. **** adj.*p* < 0.0001. Unstable vs Unchanged (adj.*p* = 3.46 × 10^−58^), Unstable vs Stable (adj.*p* = 5.41 × 10^−135^), and Unchanged vs Stable (adj.*p* = 4.01 × 10^−32^). *n* = 3 replicates per condition; each replicate represents pooled mRNA extracted from 3 mice. Source data are provided as a Source Data file.

To characterize the global poly(A) profile of each germ cell population, we quantified poly(A) tail length from our direct RNA-seq datasets using Nanopolish^29^. The transcriptome of P/D spermatocytes had predominantly poly(A) tails ~60-nt long, while the transcriptome of RS peaked with poly(A) tails ~150-nt long (Fig. 1a). The transcripts encoding lactate dehydrogenase C (*Ldhc*) and ornithine decarboxylase 1 (*Odc1*) showed poly(A) tails longer in RS than in P/D spermatocytes as previously reported (Fig. 1b and Supplementary Fig. 1f)^10,11^. Our global approach revealed that the transition to long poly(A) tail profiles was not limited to a few targets; the mean poly(A) tail length significantly increased in more than 64% (2469/3825) of the captured transcripts during the transition from P/D spermatocytes to RS (Fig. 1b). Only ~1% (54/3825) of the mRNAs showed a significant reduction in poly(A) tail length from P/D to RS, with the mRNA of cyclin A1 (*Ccna1*), a key regulator of meiotic progression, showing a pronounced shortening (Fig. 1b and Supplementary Fig. 1f)^30^. Thus, the transition from spermatocytes to spermatids is characterized by the global lengthening of transcript poly(A) tails from ~60 to ~150 nt.

Since active transcription ceases early during spermatid elongation^8,9^, the long poly(A) tail profile of RS has been proposed to provide long-term stability to transcripts so they can be used days later to support ES differentiation^10–12^. Given the transcriptional silencing in late spermiogenesis^8,9^, we inferred mRNA stability during spermiogenesis by using DESeq2 to quantify relative changes in mRNA accumulation between ES and RS from our direct RNA sequencing. Downregulated transcripts were considered unstable, while upregulated transcripts were deemed stable. Transcripts with no differential accumulation in the transition from RS to ES were referred to as unchanged (Supplementary Fig. 1g and Supplementary Data 1). Poly(A) tail profiling of RS revealed a higher accumulation of ~150-nt long tails for stable transcripts compared to the rest of the transcriptome (Fig. 1c and Supplementary Fig. 1h). This observation was confirmed at the single transcript level. Changes in mRNA accumulation across the transcriptome positively correlated with the proportion of ~150-nt long poly(A) tails in RS (Fig. 1d). The transcripts encoding sperm mitochondria associated cysteine rich protein (SMCP), protamine 2 (PRM2) and ODF1, key factors during late spermiogenesis, were among those with highest stability and accumulation of ~150-nt long tails in RS (Fig. 1d). Conversely, the unstable *Ccna1* transcripts showed a low proportion of ~150-nt tails in RS (Fig. 1d). When grouping individual transcripts according to their stability, the proportion of mRNAs with ~150-nt long poly(A) tails was on average statistically higher for stable transcripts, while unstable transcripts showed less ~150-nt tails compared to the rest of the transcriptome (Fig. 1e). Transcriptome stability through spermiogenesis was also assessed using absolute changes in mRNA accumulation between RS and ES in a published whole-testis single-cell RNA-seq dataset^28^. This independent analysis confirmed the relative stability of transcripts with ~150-nt poly(A) tails, while also accounting for the absolute decrease in transcript abundance as spermatids elongate (Supplementary Fig. 1i–k and Supplementary Data 2). Together, these results show that the accumulation of poly(A) tails ~150-nt long in RS predicts global transcript stability through spermiogenesis.

### Poly(A) profiles track RNA translation during spermiogenesis

**Fig. 2:**
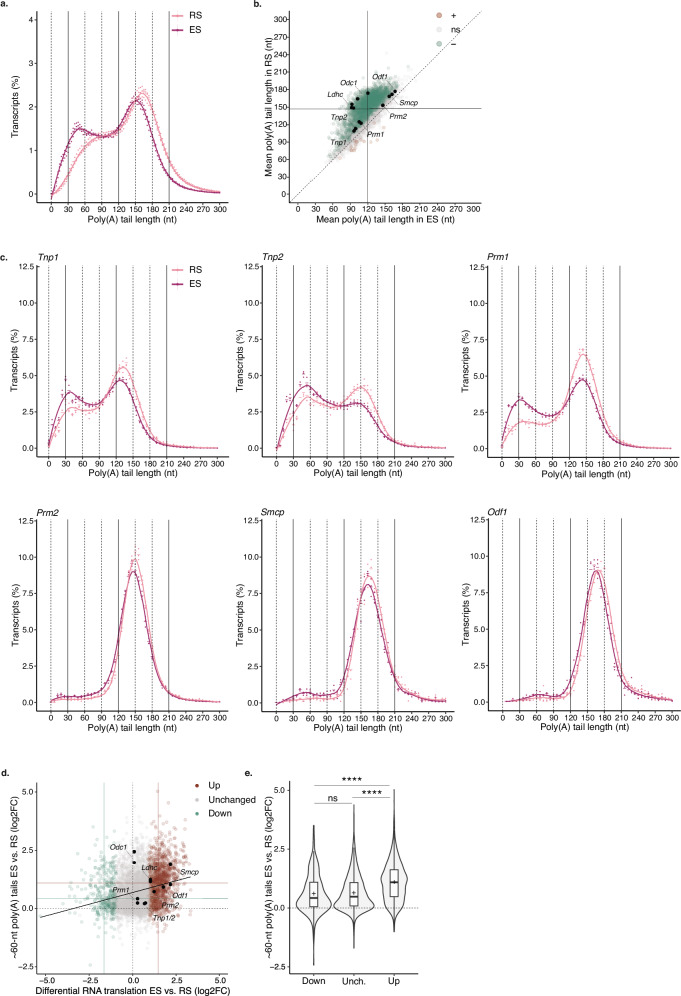
Changes in poly(A) profiles reflect the degree of mRNA translational activation during spermiogenesis. **a** Poly(A) tail length density plot for round spermatid (RS, pink) and elongated spermatid (ES, magenta) transcriptomes. Dots indicate values for individual biological replicates. The bars indicate the relative mean percentage of transcripts for each poly(A) tail length. The local polynomial regression fitting is shown as a solid line for each condition. nt nucleotide. **b** Scatter plot comparing the mean poly(A) tail length of each transcript in round spermatids (RS) and elongated spermatids (ES). Each dot represents an individual transcript. Transcripts significantly increasing (+) or decreasing (−) in poly(A) tail length during the transition are shown in brown or green, respectively; Student’s *t*-test, two-tailed, significance threshold *p* < 0.05. ns non-significant. Specific transcripts are indicated in black. **c** Poly(A) tail length density plots as in (**a**) for *Tnp1*, *Tnp2*, *Prm1*, *Prm2*, *Smcp*, and *Odf1*. **d** Scatter plot comparing fold change in transcript differential translation to the fold change in the percentage of reads per transcript with poly(A) tail ~60-nt long between RS and ES. Each dot represents an individual transcript. Transcripts with increased (Up), unchanged (Unch.) or decreased (Down) levels of translation are indicated in brown, grey and green, respectively. Solid lines indicate the medians of both variables for upregulated and downregulated mRNAs in brown and green, respectively. The linear fit is shown in black. Specific transcripts are indicated in black. **e** Violin plot showing the fold change in the percentage of reads per transcript with poly(A) tail ~60-nt long between round spermatids (RS) and elongated spermatids (ES). Transcripts are grouped by differential translation (Unch.; unchanged). The width of violins shows the density of individual transcripts. The overlaid box plots display means as crosses and medians as lines; the boxes indicate the first and third quartiles and the bars indicate the 10th and 90th percentiles. Kruskal–Wallis test using Bonferroni correction for multiple testing. **** adj.*p* < 0.0001. Down vs Unchanged (adj.*p* = 1.46 × 10^−18^), Down vs Up (adj.*p* = 1.23 × 10^−55^), Unchanged vs Up not significant (ns, adj.*p* = 9.58 × 10^−2^). *n* = 3 replicates per condition; each replicate represents pooled mRNA extracted from 3 mice. Source data are provided as a Source Data file.

Previous studies have shown that the length of transition protein 1 (*Tnp1)*, transition protein 2 (*Tnp2)*, *Prm1*, *Prm2*, *Smcp*, and *Odf1* poly(A) tails decreases during spermiogenesis progression from RS to ES^1–3^. To determine the extent of poly(A) shortening, we compared the poly(A) profiles of RS and ES transcriptomes. Although the transcriptome of ES retained a prominent ~150-nt long poly(A) tail peak, we observed a significant increase in the frequency of transcripts with ~60-nt poly(A) tails compared to RS (Fig. 2a). The increase was mostly driven by stable transcripts which showed a ~one-fold increase in the proportion of ~60-nt long poly(A) tails from RS to ES, while unstable transcripts only showed a modest increase (Supplementary Fig. 2a–c). When analyzed individually, more than 44% (2362/5290) of captured transcripts showed a significant reduction in mean poly(A) tail length during spermiogenesis progression (Fig. 2b). Consistent with previous findings, the poly(A) tail length of the transcripts encoding protamines, transition proteins, ODF1 and SMCP were significantly reduced in ES compared to RS (Fig. 2b)^1–3^. However, the amplitude of their tail shortening was modest compared to most transcripts, since although the fraction of ~60-nt long tails increased significantly, most transcripts were retained with ~150-nt long tails (Fig. 2b, c). In contrast, *Ldhc* and *Odc1*, which largely accumulated with ~150-nt long tails in RS, showed a pronounced poly(A) tail shortening back to ~60 nt in ES (Fig. 2b and Supplementary Fig. 2d). In sum, although most of the transcript poly(A) tails are shortened during spermiogenesis, the amplitude of the shortening varies widely between transcripts.

The shortening of the poly(A) tails during spermiogenesis has been previously associated with the translational activation of *Tnp1*, *Tnp2*, *Prm1*, *Prm2*, *Smcp*, and *Odf1*^1–3^. To understand whether the association is true across the transcriptome, we used previously published ribosome profiling data from RS and ES (E-MTAB-7247) to integrate translational activation during spermiogenesis to our global poly(A)-tail length analysis^31^. Transcripts accumulating with ~60-nt long tails during spermiogenesis progression were also the ones showing significant translational activation compared to the rest of the transcriptome (Fig. 2d, e and Supplementary Fig. 2e). Among the transcripts with poly(A) tail shortening to ~60 nt, *Prm2*, *Smcp* and *Odf1* showed a ~two-fold increase in translational activation (Fig. 2d). A closer inspection of their individual poly(A) profiles revealed that despite their transition to ~60 nt, most transcripts are retained with tails ~150-nt long (Fig. 2c). In contrast, transition proteins and *Prm1* transcripts showed a lower abundance of ~150-nt long tails, and modest translational activation during spermatid elongation (Fig. 2c, d). This indicates that while the transition to ~60-nt poly(A) tail is a signature of translational acceleration, the amplitude of the translational activation increases with the proportion of transcripts retained with ~150-nt tails. Taken together, these results support a model in which the most stable transcripts are stored with tails ~150-nt long in RS. During spermatid elongation, these transcripts become translationally active with tails ~150-nt long. Their poly(A) tail is gradually shortened during translation leading to accumulation of ~60-nt tails before transcripts get processed for further deadenylation and decay.

### TENT5C catalytic activity is required for spermiogenesis

We next sought to identify the enzymes responsible for poly(A) tail elongation during spermiogenesis. TENT5C is a non-canonical poly(A) polymerase shown to be highly expressed in ES^4^. We confirmed TENT5C localization using flow cytometry by tracking the fluorescent signal of germ cells from mice expressing a *Tent5c* knock-in GFP reporter allele^32^. None of the gates enriched in early or late meiotic cells showed differential fluorescence between *Tent5c*^*gfp/gfp*^ and wild-type mice, indicating the absence of significant TENT5C accumulation during meiosis (Supplementary Fig. 3a, b). However, TENT5C fluorescence was detected in isolated RS, with levels significantly increasing in the fraction enriched for ES (Supplementary Fig. 3a, b).

**Fig. 3:**
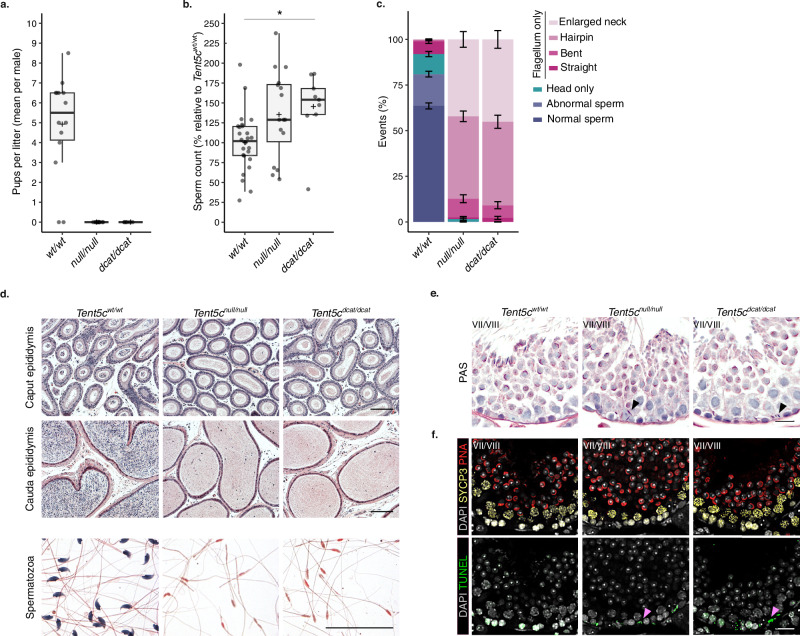
TENT5C poly(A) polymerase activity is required for fertility and spermiogenesis. **a** Box plot showing the average pups per litter obtained per adult male mouse after mating with 2 control females. *Tent5c*^*null/null*^ (*n* = 11 mice) and *Tent5c*^*dcat/dcat*^ (*n* = 6 mice) mice are compared to *Tent5c*^*wt/wt*^ (*n* = 14 mice). Each dot represents one mouse. The crosses display the means; the lines show the medians; the boxes indicate the first and third quartiles and the bars indicate the 10th and 90th percentiles. **b** Box plot as in (**a**) showing the number of flagella from cauda epididymides for *Tent5c*^*null/null*^ (*n* = 16 mice) and *Tent5c*^*dcat/dcat*^ mice (*n* = 9 mice), expressed relative to *Tent5c*^*wt/wt*^ (*n* = 26 mice). Kruskal–Wallis test using Bonferroni correction for multiple testing. * adj*.p* < 0.05. *Tent5c*^*wt/wt*^ vs *Tent5c*^*dcat/dcat*^ (adj.*p* = 1.57 × 10^−2^). *Tent5c*^*wt/wt*^ vs *Tent5c*^*null/null*^ not significant (adj.*p* = 6.10 × 10^−2^), *Tent5c*^*null/null*^ vs *Tent5c*^*dcat/dcat*^ not significant (adj.*p* = 1.00). **c** Bar plot showing the proportion of germ cell morphologies in semen isolated from the cauda epididymides of *Tent5c*^*wt/wt*^ (*n* = 18 mice)*, Tent5c*^*null/null*^ (*n* = 11 mice), and *Tent5c*^*dcat/dcat*^ (*n* = 8 mice). Quantification from Hematoxylin and Eosin (H&E) staining using the classification presented in Supplementary Fig. 3g. **d** Representative micrographs of epididymis cross sections (caput, upper; cauda, middle) and sperm from caudal epididymides (lower) stained with H&E. Sections from *Tent5c*^*null/null*^ and *Tent5c*^*dcat/dcat*^ mice are compared to *Tent5c*^*wt/wt*^. Sperm heads (nuclei) show a blue staining and flagella shades of pink. Scale, 40 μm. *n* = 3 mice per condition. **e** Representative micrographs as in (**d**) showing stage VII/VIII tubule cross sections stained with Periodic Acid Schiff (PAS). Arrowheads indicate sperm heads. Scale, 20 μm. *n* = 3 mice per condition. **f** Representative micrographs of stage VII/VIII tubule cross sections immunostaining from *Tent5c*^*wt/wt*^, *Tent5c*^*null/null*^, and *Tent5c*^*dcat/dcat*^. SYCP3 immunostaining (yellow) marks spermatocytes; PNA labeling (red) marks spermatid acrosomes, TUNEL immunostaining (green) marks apoptotic cells, and DAPI labeling (grey) marks nuclei. Arrowheads indicate apoptotic sperm heads engulfed in Sertoli cells. Scale, 20 μm. *n* = 3 mice per condition. Source data are provided as a Source Data file.

The presence of TENT5C is essential to complete spermiogenesis; *Tent5c*^*null/null*^ mice produce headless spermatozoa and are sterile (Fig. 3a–c and Supplementary Fig. 3c, d)^4,5^. Given the minor differences in RNA accumulation between whole testes of *Tent5c*^*null/null*^ and wild-type mice, it has been proposed that the poly(A) polymerase activity of TENT5C may not be required to support spermatogenesis^4^. Recently, an independent study reported that the sterility of *Tent5c*^*null/null*^ mice is associated with minor poly(A) tail changes limited to a few transcripts when considering whole testes as a starting material for sequencing^5^. To definitively answer whether the poly(A) polymerase activity of TENT5C is required for fertility, we took advantage of a previously characterized mouse strain expressing a catalytically dead version of TENT5C (*Tent5c*^*dcat/dcat*^)^32^. The body mass of the *Tent5c*^*dcat/dcat*^ and *Tent5c*^*null/null*^ mice was comparable to wild-type (Supplementary Fig. 3e). Neither *Tent5c*^*dcat/dcat*^ nor *Tent5c*^*null/null*^ mice showed differences in reproductive organ appearance and weight (Supplementary Fig. 3f). However, *Tent5c*^*dcat/dcat*^ mice, like the *Tent5c*^*null/null*^ mice, were completely infertile (Fig. 3a). To understand the origin of the sterility, we inferred germ cell production from the total number of flagella isolated from the cauda epididymides. While germ cell production was not compromised in *Tent5c* mutants, significant morphological sperm defects were observed (Fig. 3b, c). Only headless spermatozoa were found in cauda epididymis extracts from *Tent5c*^*dcat/dcat*^ and *Tent5c*^*null/null*^ animals (Fig. 3c). Isolated flagella were identified with various degree of morphological abnormalities (Fig. 3c and Supplementary Fig. 3g). About 10% were bent with an acute angle often found between the midpiece and the principal piece; ~50% of the flagella formed complete hairpins overlapping the mid and principal piece together, and ~40% displayed enlarged or coiled midpiece (Fig. 3c). Since histological analyses confirmed the presence of headless flagella in the ducts of the caput and cauda epididymides, we speculated that the morphological defects of *Tent5c*^*dcat/dcat*^ sperm originated at the testes as previously described for *Tent5c*^*null/null*^ sperm (Fig. 3d)^4^. Like *Tent5c*^*null/null*^ mice, *Tent5c*^*dcat/dcat*^ males showed retention of sperm heads close to the basement membrane of stage VII–VIII seminiferous tubules (Fig. 3e and Supplementary Fig. 3h). In addition, a TUNEL assay marked apoptotic events in the basement membrane of stage VII–VIII tubules specifically for both *Tent5c*^*null/null*^ and *Tent5c*^*dcat/dcat*^ mutants (Fig. 3f and Supplementary Fig. 3i). In sum, *Tent5c*^*dcat/dcat*^ males phenocopy the *Tent5c*^*null/null*^ mice showing sterility associated with production of headless spermatozoa, demonstrating that the poly(A) polymerase activity of TENT5C is required for spermiogenesis and fertility.

### *Insl3* and *Ptgds* are TENT5C targets in ES and Leydig cells

**Fig. 4:**
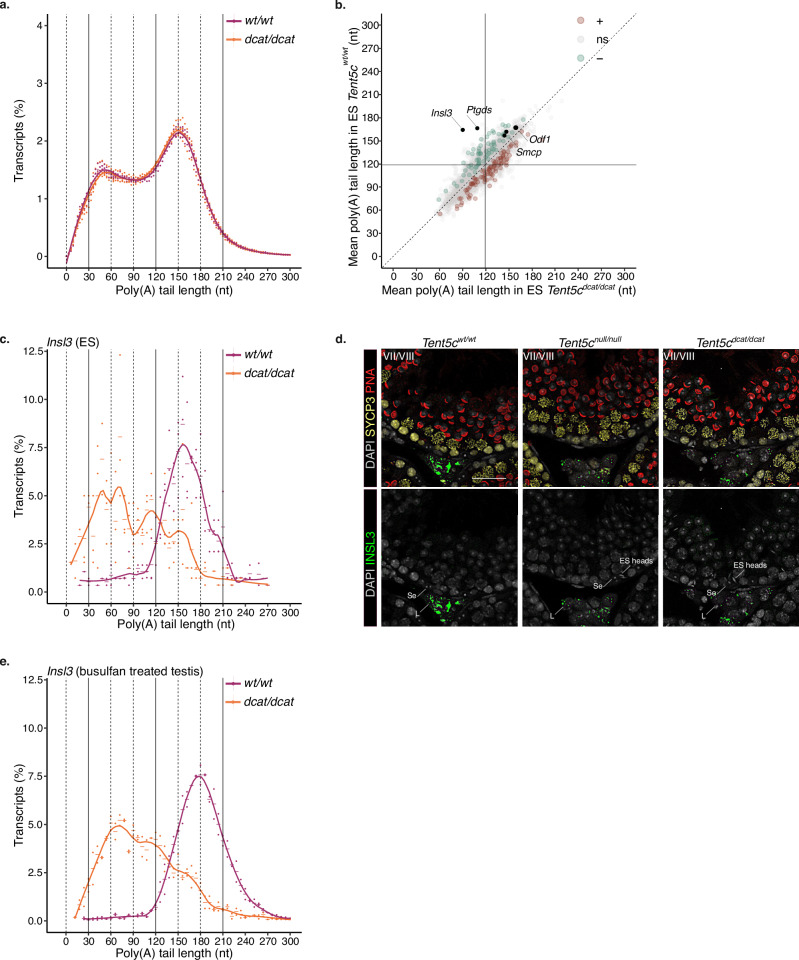
*Insl3* poly(A) tail length depends on TENT5C activity in both elongated spermatids and Leydig cells. **a** Poly(A) tail length density plot of the elongated spermatid (ES) transcriptomes of *Tent5c*^*wt/wt*^ (magenta) *and Tent5c*^*dcat/dcat*^ (orange) mice. Dots indicate values for individual biological replicates. The bars indicate the relative mean percentage of transcripts for each poly(A) tail length. The local polynomial regression fitting is shown as a solid line for each condition. nt nucleotide. *n* = 3 replicates per condition; each replicate represents pooled mRNA extracted from 3 mice. **b** Scatter plot comparing the mean poly(A) tail length of each transcript in elongated spermatids (ES) of *Tent5c*^*wt/wt*^ and *Tent5c*^*dcat/dcat*^ mice. Each dot represents an individual transcript. Transcripts significantly increasing (+) or decreasing (−) in poly(A) tail length between genotypes are shown in brown or green, respectively; Student’s *t*-test, two-tailed, significance threshold *p* < 0.05. ns non-significant. *n* = 3 replicates per condition; each replicate represents pooled mRNA extracted from 3 mice. **c** Poly(A) tail length density plot as in (**a**) for *Insl3* transcripts from the elongated spermatids (ES) of *Tent5c*^*wt/wt*^ (magenta) *and Tent5c*^*dcat/dcat*^ (orange) mice. *n* = 3 replicates per condition; each replicate represents pooled mRNA extracted from 3 mice. **d** Representative micrographs of stage VII/VIII tubule cross-sections from *Tent5c*^*null/null*^ (*n* = 4 mice) and *Tent5c*^*dcat/dcat*^ mice (*n* = 4 mice), compared to *Tent5c*^*wt/wt*^ (*n* = 7 mice). INSL3 immunostaining in green; SYCP3 immunostaining (yellow) marks spermatocytes; PNA labeling (red) marks spermatid acrosomes, and DAPI labeling (grey) marks nuclei. Se Sertoli cells, L Leydig cells, ES Elongated Spermatids. Scale, 20 μm. **e** Poly(A) tail length density plot as in (**a**) for *Insl3* transcripts from whole testes of *Tent5c*^*wt/wt*^ (magenta) and *Tent5c*^*dcat/dcat*^ (orange) busulfan-treated mice. *n* = 2 mice per condition. Source data are provided as a Source Data file.

To identify the transcripts targeted by the poly(A) polymerase activity of TENT5C during spermiogenesis, we performed poly(A) profiling of RS and ES from wild-type and *Tent5c*^*dcat/dcat*^ mice. In the absence of TENT5C poly(A) polymerase activity, we anticipated the poly(A) tail shortening of TENT5C-targeted transcripts. The global poly(A) distributions of the RS and ES transcriptomes were almost identical between *Tent5c*^*dcat/dcat*^ and wild-type mice (Fig. 4a, b and Supplementary Fig. 4a, b). Moreover, DESeq2 analysis on direct RNA-sequencing data from purified RS and ES cells detected no significant differences in relative transcript abundance between the two genotypes (Supplementary Data 1). These results show that TENT5C poly(A) polymerase activity is not required to shape the overall RNA poly(A) tail length profiles, nor transcript composition during spermiogenesis. Instead, we observed a significant poly(A) tail shortening restricted to a specific set of transcripts in RS and ES of *Tent5c*^*dcat/dcat*^ mice (Fig. 4b and Supplementary Fig. 4b). Among them, insulin-like 3 (*Insl3*) and prostaglandin D2 synthase (*Ptgds*) mRNAs stand out as putative TENT5C targets. *Insl3* transcripts showed the highest reduction in mean poly(A) tail length in both RS and ES of *Tent5c*^*dcat/dcat*^ mice compared to wild-type (Fig. 4b and Supplementary Fig. 4b). In ES, *Ptgds* transcript was the second transcript with the highest amplitude of poly(A) tail shortening (Fig. 4b). To better understand the nature of *Insl3* and *Ptgds* tail shortening, we examined their individual poly(A) length profiles. While *Insl3* and *Ptgds* mRNAs accumulate with tails ~150-nt long in wild-type ES, the vast majority of *Insl3* and *Ptgds* transcripts shifted to ~60-nt long poly(A) tails in *Tent5c*^*dcat/dcat*^ mutants (Fig. 4c and Supplementary Fig. 4c). Given that the accumulation of tails ~60-nt long is associated with translational activation, we anticipated an increase in INSL3 protein abundance in *Tent5c*^*dcat/dcat*^ ES. However, no ectopic signal for INSL3 protein was detected in any of the germ cells from wild-type and *Tent5c*^*dcat/dcat*^ testes (Fig. 4d). Instead, INSL3 expression was restricted to Leydig cells as previously described^5^. Thus, although TENT5C catalytic activity is required to sustain *Insl3* poly(A) tail length, the shortening of *Insl3* poly(A) tail in *Tent5c*^*dcat/dcat*^ mutants does not appear to be sufficient to trigger translation of *Insl3* transcript in ES.

*Insl3* transcripts and proteins accumulate at high levels in Leydig cells, but their role in sustaining spermatogenesis after testis descent remains unclear^33^. A closer inspection of our immunostained samples revealed a decrease in INSL3 accumulation in the Leydig cells of *Tent5c*^*dcat/dcat*^ mice compared to wild-type, as recently shown by Brouze and colleagues (Fig. 4d and Supplementary Fig. 4d)^5^. To understand if the decrease in INSL3 protein levels in Leydig cells was associated with changes in the *Insl3* poly(A) tail length in the absence of TENT5C activity, we enriched for interstitial cells the testes of wild-type and *TENT5c*^*dcat/dcat*^ mice by depleting germ cells with busulfan (Supplementary Fig. 4e). Immunostaining confirmed a decrease in INSL3 protein accumulation in *Tent5c*^*dcat/dcat*^ Leydig cells even after busulfan injection (Supplementary Fig. 4f). Sequencing of busulfan-treated testes revealed *Insl3 and Ptgds* transcripts mostly accumulating with poly(A) tails ~180-nt long in wild-type samples (Fig. 4e and Supplementary Fig. 4g). In *Tent5c*^*dcat/dcat*^ busulfan-treated testes, *Insl3* and *Ptgds* transcripts showed gradual deadenylation down to ~60 nt with a significant depletion of the ~180-nt long tail population (Fig. 4e and Supplementary Fig. 4g). Poly(A) tail shortening was associated with the downregulation of *Insl3* transcripts, when comparing relative abundance between busulfan-treated testes of wild-type and *Tent5c-*mutant mice by direct RNA-sequencing (Supplementary Fig. 4h and Supplementary Data 1). Together, these results indicate that TENT5C poly(A) polymerase is required to maintain or readenylate *Insl3* poly(A) tails up to ~180-nt long in Leydig cells and ~150-nt long in ES. In contrast to in ES, *Insl3* transcripts with long tails in Leydig cells were translationally active, given the high accumulation of INSL3 protein in these cells (Fig. 4d). Thus, the decrease in INSL3 protein levels in the Leydig cells from *Tent5c* mutants could be explained by a depletion of the translationally active transcripts in the absence of TENT5C-mediated adenylation. As *Insl3* poly(A) tails get shorter, transcripts may be triggered to decay, resulting in their downregulation.

### TENT5C activity extends *Odf1* poly(A) tails in ES

**Fig. 5:**
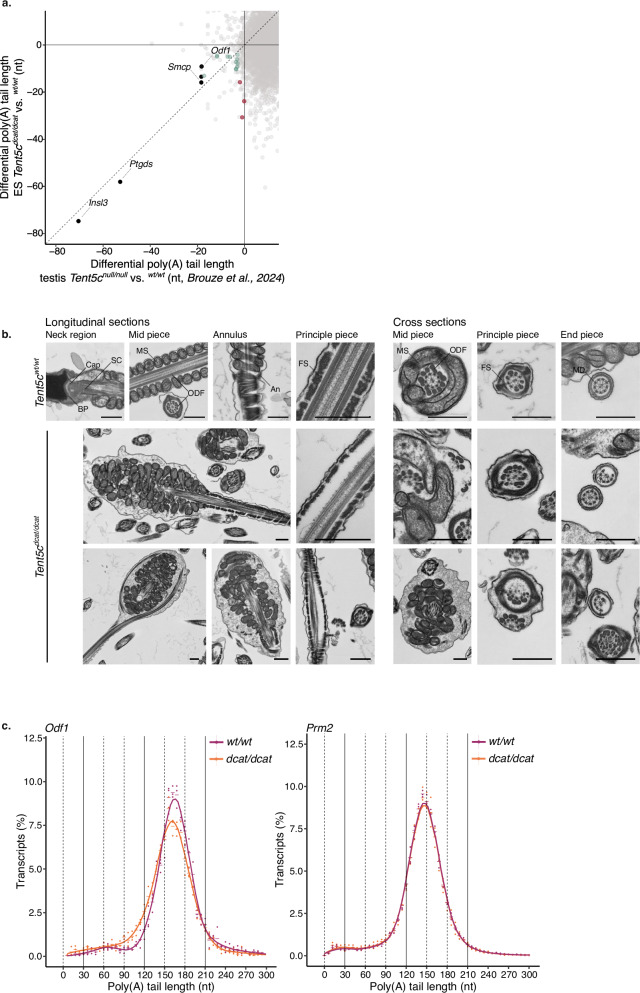
TENT5C activity extends *Odf1* poly(A) tails in elongated spermatids. **a** Scatter plot comparing the differential poly(A) tail length of individual transcripts from the elongated spermatids (ES) of *Tent5c*^*wt/wt*^ and *Tent5c*^*dcat/dcat*^ mice (this study) and the differential poly(A) tail length of the corresponding transcripts from the whole testes of *Tent5c*^*wt/wt*^ and *Tent5c*^*null/null*^ mice^5^. Each dot represents an individual transcript. Transcripts significantly decreasing in poly(A) tail length in ES or whole testes are shown in purple and green, respectively. The transcripts showing significance in both studies are shown in black and labeled by their gene symbol. nt nucleotide. *n* = 3 replicates per condition; each replicate represents pooled mRNA extracted from 3 mice. **b** Representative transmission electron micrographs of sperm from the cauda epididymides of *Tent5c*^*dcat/dcat*^ mice compared to *Tent5c*^*wt/wt*^. BP basal plate, Cap capitulum, SC striated columns, MS mitochondrial sheath, ODFs outer dense fibers, An annulus, FS fibrous sheath, MD microtubule doublets. Scale, 600 nm. **c** Poly(A) tail length density plot of *Odf1* and *Prm2* in elongated spermatids (ES) from *Tent5c*^*wt/wt*^ (magenta) *and Tent5c*^*dcat/dcat*^ (orange) mice. Dots indicate values for individual biological replicates. The bars indicate the relative mean percentage of transcripts for each poly(A) tail length. The local polynomial regression fitting is shown as a solid line for each condition. *n* = 3 replicates per condition; each replicate represents pooled mRNA extracted from 3 mice. Source data are provided as a Source Data file.

To further understand the physiological relevance of TENT5C polyadenylation for sperm morphogenesis, we revisited our list of putative TENT5C targets in ES. In addition to *Insl3* and *Ptgds*, *Smcp*, and *Odf1* were identified as two of the four transcripts showing a significant reduction in poly(A) tail length in both *Tent5c*^*dcat/dcat*^ ES (this study) and Tent5c^*null/null*^ whole testes (Fig. 5a)^5^.

ODF1 is a core component of the outer dense fibers (ODFs), a cytoskeletal structure which runs externally to the axoneme and provides the flagella with elastic properties and resistance to shear forces^34–36^. ODF1 also concentrates at the head–tail coupling apparatus (HTCA), a specialized structure at the spermatid neck that mediates the tight attachment of the sperm tail to the nucleus^37–39^. As a result, ODF1 depletion leads to altered ODF arrangement, sperm decapitation, and sterility^6^. A previous study proposed the disassembly of the head–tail connecting piece as a main mechanism explaining the phenotype of TENT5C-depleted sperm^4^. To further investigate the effects of TENT5C deficiency—and ODF1 poly(A) tail dysregulation—on sperm ultrastructure, sperm pellets extracted from the cauda epididymides were prepared for transmission electron microscopy. Sperm from wild-type mice showed a typical organization of the HTCA, with discernible basal plate, capitulum, and segmented columns (Fig. 5b). Only headless spermatozoa were captured in TENT5C*-*deficient mice. The midpiece of wild-type spermatozoa showed a typical helical coiled sheath of mitochondria organized around a regular assembly of ODFs tightly linked to the nine outer doublet microtubules of a unique axoneme (Fig. 5b). In contrast, TENT5C-deficient flagella presented disturbed structural organization of the mitochondrial sheath. Mitochondria formed multilayered aggregates, and multiple axonemal structures, often incomplete and asymmetric, were observed within the same cytoplasm (Fig. 5b). Longitudinal sections revealed looping of irregular axonemal structures around disorganized mitochondria. Additionally, a mix of mitochondria and fibrous sheath suggested abnormal annulus formation in the mutant cells (Fig. 5b). Aberrations in the principal piece of mutant sperm included disorganized or absent microtubule doublets, along with missing or misaligned ODFs (Fig. 5b). In several cross-sections, the ODFs were displaced to the inner side of the microtubule doublet, not in direct contact with the fibrous sheath (Fig. 5b). Longitudinal sections occasionally revealed gaps between the axoneme and fibrous sheath leading to protrusions along the principal piece (Fig. 5b). These defects closely resembled those characterized in *Odf1*-mutant mice^6^. Therefore, *Odf1* is a strong candidate to causally explain the headless phenotype and flagellar defects observed in *Tent5c*-mutant sperm.

To determine whether other transcripts encoding components of the HTCA/ODFs axis might also be dependent on TENT5C poly(A) polymerase activity, we relied on an exhaustive list of known, predicted, and candidate HTCA/ODF factors implicated in acephalic spermatozoa syndrome and male infertility^38^. This set included coiled-coil domain containing 42 and sperm-associated antigen 4 (SPAG4), which form a complex with ODF1 at the HTCA^37,40^, ODF2, a direct ODF1 interactor at the ODFs^41^, and WD repeat domain 64 (WDR64), reported to interact with ODF1 at the manchette^42^. Notably, none of these transcripts showed reduced poly(A)-tail length in *Tent5c-*mutant ES cells. Within this HTCA/ODF-focused set, *Odf1* was the only transcript that emerged as a TENT5C-dependent polyadenylation target, consistent with a specific link to the head–tail coupling phenotype (Supplementary Data 3).

As for the transition proteins and protamine mRNAs, the post-transcriptional regulation of *Odf1* transcripts is characterized by changes in their poly(A) tail processing^1^. While in RS, *Odf1* transcripts accumulate with tails ~150-nt long and are associated with the non-polysomal fraction, in ES, they enter the polysomal fraction with tails ~150-nt long, which get gradually shortened^1^. Consistently, our sequencing methodology recapitulated the changes in *Odf1* poly(A) tail length observed in the transition from RS to ES in wild-type mice (Fig. 2c). To understand the impact of TENT5C on the poly(A) processing of *Odf1*, we examined their poly(A) tail profile in ES from *Tent5c*^*dcat/dcat*^ animals. *Odf1* showed a decrease in transcripts with ~150-nt poly(A) tails together with an increase in ~60-nt long tails in ES expressing the catalytically mutant TENT5C when compared to wild-type ES (Fig. 5c). The shortening of *Odf1* poly(A) tails observed in the absence of TENT5C activity is comparable to the one observed for the same transcripts during spermiogenesis progression, where a mild accumulation of ~60-nt long tails reflects the translational activation of long-tailed mRNAs^1^ (Fig. 2c, d). In contrast, *Prm2*, which shares a similar poly(A) profile to *Odf1* in the transition from RS to ES, showed no poly(A) profile alteration in *Tent5c*^*dcat/dcat*^ ES (Fig. 5c). Thus, the poly(A) polymerase activity of TENT5C is specifically required to maintain *Odf1* transcripts with ~150-nt poly(A) tails in ES.

### TENT5C promotes ODF1 deposition at the late spermatid neck

We next interrogated possible changes in ODF1 protein abundance between *Tent5c* mutants and wild-type mice during spermiogenesis. Because ODF1 accumulation and distribution are highly dynamic during late spermatid differentiation, we performed a stage-specific analysis of ODF1 immunofluorescence on testis sections. In wild-type mice, ODF1 staining was first detected in step-14 spermatids (stage III–IV) and remained elevated until spermiation (Supplementary Fig. 5a). The staining appeared diffuse across the cytoplasm with enrichment in discrete aggregates. Throughout spermatogenesis, ODF1-positive granules were observed at the base of seminiferous tubules, likely reflecting the elimination of ODF1 in residual bodies, as previously described in rats (Supplementary Fig. 5a)^43^. In *Tent5c*^*null/null*^ and *Tent5c*^*dcat/dcat*^ mice, we observed consistent defects in ODF1 protein accumulation and distribution. The cytoplasmic ODF1 intensity and the area of ODF1 aggregates were reduced specifically in step-16 spermatids (stage VII–VIII) of *Tent5c* mutants compared to wild-type (Supplementary Fig. 5a–d). Throughout all spermatogenic stages, the frequency of ODF1-positive granules found across the epithelium was markedly reduced, with only occasional isolated single granules (Supplementary Fig. 5a).

**Fig. 6:**
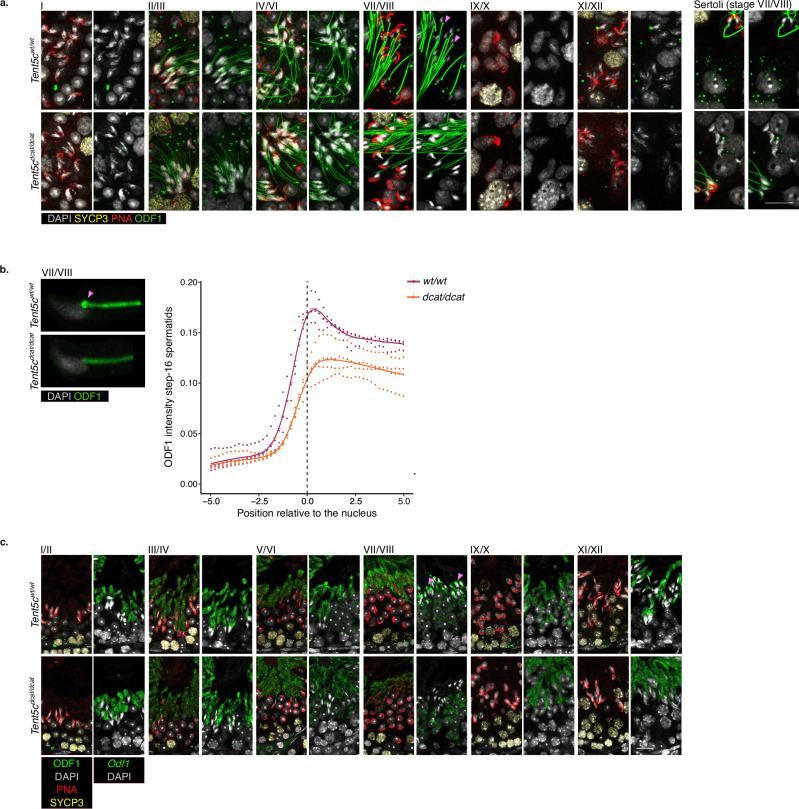
TENT5C polyadenylation promotes the accumulation of *Odf1* mRNA and protein at the neck of late-stage spermatids. **a** Representative micrographs of stage I/II to XI/XII cells from whole-tubule squashes of *Tent5c*^*dcat/dcat*^ mice (*n* = 4 mice) compared to *Tent5c*^*wt/wt*^ (*n* = 3 mice). ODF1 immunostaining in green; SYCP3 immunostaining (yellow) marks spermatocytes; PNA labeling (red) marks spermatid acrosomes, and DAPI labeling (grey) marks nuclei. Arrowheads indicate ODF1 accumulation at the HTCA. Scale, 20 μm. **b** ODF1 line intensity profiles of step-16 spermatids from whole-tubule squashes of *Tent5c*^*dcat/dcat*^ mice (*n* = 4 mice) compared to *Tent5c*^*wt/wt*^ (*n* = 3 mice). Individual spermatids were aligned to the base of the nucleus, defined as coordinate 0. Dots indicate normalized intensity values for individual biological replicates. The bars indicate the mean ODF1 intensity for each position and solid lines show loess smoothing for each genotype. Representative micrographs of step-16 spermatids immunostained for ODF1 and DAPI are shown for both genotypes. Arrowheads indicate ODF1 accumulation at the HTCA. **c** Representative micrographs of stage I/II to XI/XII tubule cross-sections from *Tent5c*^*dcat/dcat*^ mice compared to *Tent5c*^*wt/wt*^. Adjacent serial sections are shown for ODF1 immunostaining (left) and *Odf1* RNA FISH (right), both in green. SYCP3 immunostaining (yellow) marks spermatocytes; PNA labeling (red) marks spermatid acrosomes, and DAPI labeling (grey) marks nuclei. Arrowheads indicate *Odf1* accumulation at the neck of step-16 spermatids. Scale, 20 μm. *n* = 3 mice per condition. Source data are provided as a Source Data file.

To better visualize ODF1 distribution along the developing flagellum, we performed immunostaining on squash preparations of seminiferous tubules. In wild-type spermatids, ODF1 signal first appeared along the flagellar midpiece at step 14 (stage II–III) (Fig. 6a). By step 16 (stage VII–VIII), ODF1 intensity along the flagellum increased, with a prominent enrichment at the base of the nucleus indicative of ODF1 concentration at the HTCA (Fig. 6a, b)^37,38^. ODF1-positive granules were detected near step-14 spermatids (stage II–III) (Fig. 6a). By stage VII–VIII, these granules were observed in regions enriched for Sertoli cells, consistent with residual-body trafficking (Fig. 6a). In *Tent5c*^*dcat/dcat*^ mice, quantitative profiling of ODF1 signal revealed a decrease in ODF1 intensity along the flagellum of step-16 spermatids (stage VII–VIII) when compared to wild-type mice, with a prominent reduction of ODF1 accumulation at the base of the nucleus (Fig. 6b and Supplementary Fig. 5e). Note that this defect is specific to the last step of spermiogenesis as ODF1 intensities in step-15 spermatids (stage IV–VI) are comparable between genotypes (Supplementary Fig. 5f). In *Tent5c*^*dcat/dcat*^ squash preparations, ODF1-positive granules were readily detected around step-14 and -15 spermatids (stage II–VI) like in wild-type but largely disappeared by step-16 (stage VII–VIII), with only rare events observed across other stages (Fig. 6a). Consistent with an effect restricted to the final step of spermatid differentiation, mass spectrometry on bulk RS and ES populations did not capture changes in ODF1 protein abundance between *Tent5c*^*wt/wt*^ and *Tent5c*^*dcat/dcat*^ mice (Supplementary Data 4).

To determine whether the aberrant ODF1 accumulation was preserved in mature sperm, we immunostained sperm isolated from the cauda epididymides. In wild-type mice, ODF1 localized at the base of the sperm head, distributed uniformly along the midpiece to gradually decrease in intensity along the principal piece (Supplementary Fig. 5g). In *Tent5c*^*dcat/dcat*^ mice, ODF1 signal was heterogenous along malformed midpieces, clustering at sites where the flagellum fused to form hairpins (Supplementary Fig. 5g). Aberrant loci of high intensity were detected along the principal piece, down to the distal end of the flagellum (Supplementary Fig. 5g). Together, these results show that TENT5C poly(A) polymerase activity is required to maintain ODF1 accumulation along the late-spermatid flagellar midpiece, and particularly at the HTCA where ODF1 secures head–tail linkage. In sperm from *Tent5c* mutants, ODF1 mislocalization may contribute to the local disorganization of periaxonemal structures.

To evaluate whether the observed effects were restricted to ODF1 or reflected broader structural changes, we extended our investigation to additional HTCA- and ODF-associated proteins. Along with ODF1, ODF2 is the main component of the ODFs surrounding the axoneme^41^. Although the dynamics of ODF2 accumulation and its localization during spermiogenesis were comparable between genotypes, ODF2 signal intensity was increased along the midpiece of step-16 spermatids (stage VII/VIII) in *Tent5c* mutants compared with controls (Supplementary Fig. 5h–k). This may reflect enhanced recruitment of ODF2 to the ODFs and/or increased antigen accessibility where ODF1 accumulation is reduced. Both ODF1 and TENT5C have been shown at the manchette, a transient structure coordinating the formation of the HTCA^42^. Immunostaining for the manchette marker WDR64 showed comparable intensity and distribution between *Tent5c* mutants and wild-type controls (Supplementary Fig. 5l), indicating that manchette integrity is preserved in the absence of TENT5C poly(A) polymerase activity. Subcellular aggregates such as chromatoid remnants and reticulated bodies act as local mRNA translation hubs associated with ODF1 protein in spermatids^44^. In *Tent5c* mutants, these granules showed normal intensity and positioning as evidence by testis-specific serine kinase 2 (TSSK2) immunostaining on squash preparations (Supplementary Fig. 5m). Together, these results show that loss of TENT5C-dependent mRNA polyadenylation selectively reduces ODF1 protein abundance at the head–tail transition of late spermatids while preserving major structural and mRNA-regulatory complexes.

In addition to regulating mRNA translation, poly(A) tail length is known to influence transcript stability and subcellular distribution^45^. To assess the impact of TENT5C-dependent polyadenylation on *Odf1* transcript accumulation and localization, we next performed RNA FISH on testis sections from *Tent5c*^*wt/wt*^ and *Tent5c*^*dcat/dcat*^ mice. In wild-type mice, *Odf1* mRNA signal was first detected in stage V/VI tubules, diffusely distributed in the cytoplasm of step 5–6 RS (Fig. 6c). Signal intensity increased in the cytoplasm of step-9 spermatids (stage IX) (Fig. 6c). In step-16 spermatids (stage VII/VIII), *Odf1* transcripts accumulated in distinct aggregates near the spermatid nucleus (Fig. 6c). In *Tent5c*^*dcat/dcat*^ mice, *Odf1* transcripts showed comparable intensity and distribution across most stages of spermiogenesis relative to wild-type (Fig. 6c). However, *Odf1* mRNA aggregates were not detectable in step-16 spermatids of *Tent5c* mutants (Fig. 6c). Notably, DESeq2 analysis on direct RNA sequencing data from purified RS and ES cells detected no significant differences in *Odf1* abundance between *Tent5c*^*dcat/dcat*^ and wild-type (Supplementary Data 1). This discrepancy likely reflects the limited sensitivity and stage resolution of the approach for detecting decreases in RNA-abundance that arise specifically at the final step of spermatid differentiation.

Together, these results suggest that TENT5C polymerase activity is required to maintain *Odf1* poly(A) tails ~150-nt long in ES, thereby promoting transcript stability and local accumulation near the nucleus to support translation and deposition of ODF1 proteins at the HTCA during late spermiogenesis. In the absence of TENT5C activity, the pool of translationally active *Odf1* transcripts decreases, and *Odf1* mRNAs become unstable, leading to their loss at the latest stage of spermatid differentiation. Together, these effects could contribute to the overall reduction of ODF1 protein at the HTCA and the midpiece in *Tent5c*-mutant spermatids, thereby compromising head–tail fastening and flagellar morphology.

## Discussion

Transition proteins, protamines, *Smcp*, and *Odf1* mRNAs are transcribed in RS, but they only become translationally active as spermatids elongate^1–3^. This temporal separation between transcription and translation suggests that long-term mRNA stabilization is essential for proper spermiogenesis^8,9^. Although poly(A) tails are thought to contribute to RNA storage, observations are mostly limited to a few transcripts^10–12^. Using global and quantitative approaches, we found that from spermatocytes to RS, most transcripts gain longer poly(A) tails. The factors mediating the establishment of the ~150-nt long poly(A) tails in RS remain unclear. The testis-specific poly(A) polymerase tPAP/PAPOLB, which predominantly accumulates in pachytene spermatocytes and RS, extends the poly(A) tails of *Ldhc*, *Odc1*, and specific transcription factor mRNAs in RS^10–12^. Although tPAP activity is essential to support spermatids development, the extent of its activity across the transcriptome is unknown^10^. The emergence of longer poly(A) tails in RS may also result from the de novo synthesis of mRNAs with long tails stabilized at ~150 nt. By contrast, TENT5C is unlikely to account for the widespread polyadenylation observed in RS, as its expression peaks later, in ES, where it maintains the polyadenylation of a limited set of mRNAs, including *Odf1*. Understanding how the RS transcriptome is stabilized with ~150-nt long tails is required to gain insights into mRNA metabolism during spermiogenesis progression.

During spermatid elongation, the translational activation of previously studied transcripts correlates with poly(A) tail shortening^1–3^. Our study shows that this association applies to the whole transcriptome during spermiogenesis. We observed that the emergence of ~60-nt tails is a robust indication of active translation. However, the intensity of the translational activation is proportional to the abundance of transcripts retained with ~150-nt tails. Consistently, previous work has shown that most *Smcp* and *Prm2* transcripts associated with high polysomal fractions have tails ~150-nt long^3^. Thus, translational activation occurs prior to deadenylation and does not require poly(A) tail shortening. Instead, the shortening from ~150- to ~60-nt long tails during spermiogenesis appears to be a byproduct of active translation. In this way, comprehensive poly(A) tail profiles can be used as specific and sensitive resources to infer the temporal dynamics of translation during spermiogenesis. For example, *Prm2* transcripts encode for the last protamine to be expressed in late spermatids^46^. As such, *Prm2* transcripts primarily accumulate with poly(A) tails ~150-nt long in ES. In contrast, *Prm1* mRNAs show a reduced proportion of ~150-nt tails and an accumulation of ~60-nt tails, indicating earlier translational events.

Proteins like PABPC1 and the Y box proteins control the translation of mRNAs in spermatids^47–50^. In the cytoplasm, PABPC1s prevent mRNA deadenylation by coating the poly(A) tails, with each PABPC1 covering ~27 nt^51^. Although PABPC1s can favor translation, in excess, they compete with the translational activation of spermatid mRNAs, ultimately leading to sterility^47^. Thus, in spermatids, 5 to 6 PABPC1s may bind and protect the transcript poly(A) tails from deadenylation and regulate the translational fate of mRNAs. Y-Box protein 2 (YBX2) and Y-Box protein 4 (YBX4) repress the translation of transcripts with long poly(A) tails in spermatids^48–50^. Upon YBX2 depletion, the premature entry of *Prm1* and *Smcp* into the polysomal fraction results in spermiogenesis arrest^48,49^. Similarly, maintaining high levels of YBX4 prevents the normal translational activation of transcripts such as *Tnp2* and *Odf2*^50^. The translational repression by YBX2 and YBX4 may prevent the premature deadenylation of mRNAs in RS, securing their storage for protein synthesis in ES.

How the poly(A) tail polymerase activity of TENT5C may control mRNA metabolism in spermatids remained unclear^4,5^. Here, we show that TENT5C activity is required to maintain the length of *Insl3* and *Ptgds* poly(A) tails at ~150 nt in ES, but the redistribution of *Insl3* tails to ~60-nt in *Tent5c*^*dcat/dcat*^ ES was not associated with *Insl3* translational activation. These observations support that tail shortening is not a prerequisite for active translation. In Leydig cells, *Insl3* and *Ptgds* accumulate with ~180-nt long tails, ~27 nt longer than in spermatids, indicating that they are regulated by a different ribonucleoprotein (RNP) complex with one additional PABP^51^. Unlike the RNP to which *Insl3* is associated in spermatids, the Leydig complex renders *Insl3* translationally active as shown by the presence of INSL3 protein in the interstitial compartment. As observed in spermatids, the maintenance of *Insl3* and *Ptgds* poly(A) tails above 150 nt depends primarily on TENT5C. In the absence of TENT5C poly(A) polymerase activity, both the protein and the mRNA levels of *Insl3* decrease, supporting a model where TENT5C activity is required to maintain translationally active transcripts. In the absence of TENT5C, the shortening of *Insl3* tails may contribute to transcript destabilization and decay.

INSL3 and PTGDS are both required for testicular descent; *Insl3*- and *Ptgds*-deficient animals are cryptorchid and hemi-cryptorchid, respectively^52–54^. Since *Tent5c* mutants do not show obvious aberrations in testis position, TENT5C does not appear to play a critical role during testicular development. The role of INSL3 in sustaining adult spermatogenesis is unclear^33^. *Insl3*-deficient animals are sterile due to cryptorchidism, but their fertility can be recovered if the testes are artificially descended, albeit with very low efficiency^55^. Relaxin/insulin-like family peptide receptor 2 (RXFP2) is the cognate receptor for INSL3. Global *Rxfp2*-knockout mice are cryptorchids as expected; however, when *Rxfp2* is specifically removed from the germline, males sire normal-size litters^56,57^. These observations indicate that INSL3 depletion does not directly impact spermatids. Given the morphological abnormalities identified in *Tent5c*^*dcat/dcat*^ sperm, we also examined TENT5C targets encoding for structural proteins.

ODF1 and SMCP are structural proteins required for sperm integrity^6,58^. We identified *Odf1* and *Smcp* as putative targets of TENT5C poly(A) polymerase activity in ES. Consistently, both transcripts show a poly(A) tail shortening of similar amplitude in global testis samples of *Tent5c*-null mice^5^. Although the changes in poly(A) tail in mutant animals were modest, a reduction in *Odf1* and *Smcp* poly(A) tail length of comparable magnitude was observed upon translational activation during spermiogenesis. Therefore, we deem these transcripts to be high-confidence TENT5C targets and propose that the shortening of *Odf1* and *Smcp* poly(A) tails in the absence of TENT5C activity affects their stability and translation.

RNA FISH and high-resolution immunostaining show that in step-16 spermatids *Odf1* mRNA and ODF1 protein concentrate in discrete aggregates at the neck, consistent with localized *Odf1* mRNA promoting ODF1 protein accumulation at the HTCA. Importantly, this localization depends on TENT5C polyadenylation: in Tent5c^*dcat/dcat*^ step-16 spermatids, *Odf1* mRNA aggregates are lost, and ODF1 protein is markedly reduced along the flagellum, with the strongest decrease at the HTCA. Together, these data indicate that TENT5C poly(A) polymerase activity is required to maintain *Odf1* mRNA aggregates and robust ODF1 protein accumulation at the HTCA, thereby supporting head–tail fastening.

While direct physical association between TENT5C and *Odf1* mRNAs remains to be established, existing observations support a coherent mechanistic model for how they may interact. TENT5C localizes primarily to the manchette of ES and becomes undetectable as the *Odf1* mRNA granules form^4^. The manchette is a transient microtubule scaffold known to deliver factors required for HTCA assembly and flagellum development^59^. We propose that during early spermatid elongation, TENT5C-mediated polyadenylation may stabilize a pool of *Odf1* transcripts at the manchette. As the manchette collapses toward the developing HTCA, these stabilized *Odf1* transcripts persist and aggregate into granules at the neck of step-16 spermatids, where their local translation supports ODF1 accumulation at the HTCA to secure head–tail connection.

The HTCA is a centrosome-based structure derived from the spermatid centrioles^60^. The translational control of mRNAs at centrioles is a conserved regulatory mechanism across animals^61^. In the Drosophila embryo, Oo18 RNA-binding protein supports the local synthesis of pericentrin-like protein at the centrosomes by promoting polyadenylation^62^. Similarly, residual TENT5C might associate with centriole-derived structures to further support *Odf1* mRNA stability and translation at the HTCA of late spermatids. The centriolar protein polo-like kinase 4, a known direct interactor of TENT5C, could mediate its retention at the spermatid neck^63,64^. However, such an interaction, if present in spermatids, would likely involve only a small fraction of TENT5C, as the protein falls below the detection limit in step-16 spermatids^4^.

In *Tent5c*^*dcat/dcat*^ males, ODF1 protein remains detectable and is reduced only at the terminal stage of spermatid differentiation. This would predict a phenotype analogous to *Odf1* heterozygosity^6^. However, *Odf1* heterozygosity is not sufficient to induce sperm decapitation, as headless spermatozoa are observed exclusively in *Odf1*-null animals^6^. These observations suggest that head–tail dissociation in *Tent5c*-mutant mice is unlikely to be explained by an overall reduction in ODF1 abundance. Instead, our data support a model in which the spatial control of *Odf1* translation is a critical determinant of head–tail integrity: in the absence of TENT5C-dependent polyadenylation, failure to stabilize *Odf1* mRNAs in neck aggregates and the resulting failure to concentrate ODF1 synthesis at the HTCA may be sufficient to trigger head–tail dissociation and recapitulate key aspects of the *Odf1*-null phenotype. An additional possibility is that perturbation of other HTCA-related factors could synergize with impaired ODF1 enrichment, rendering the combined defects sufficient to induce sperm decapitation. However, none of the other HTCA-associated transcripts examined in *Tent5c-*mutant ES cells showed reduced poly(A) tail length; *Odf1* was the only transcript that emerged as a TENT5C-dependent polyadenylation target with direct relevance to HTCA integrity, consistent with a central contribution to the headless-sperm phenotype.

In addition to sperm decapitation, *Tent5c*-mutant sperm show defects in periaxonemal structures, including abnormal ODF architecture, fibrous sheath protrusions, and disrupted mitochondrial organization. These abnormalities closely resemble those reported in *Odf1*-mutant mice and can be directly associated with reduced ODF1 accumulation along the late-spermatid flagellar midpiece in the absence of TENT5C activity^6^. Although the axoneme is largely assembled by steps 15–16, periaxonemal structures continue to mature in a tightly coordinated program^65–67^; because ODF1 is a core component of the periaxonemal scaffold, reduced ODF1 enrichment along the midpiece could contribute to broader ultrastructural defects of other accessory structures. Importantly, preserved localization of TSSK2-positive RNA granules at the annulus and ODF2 along the ODFs argues against a global failure of periaxonemal assembly in *Tent5c*-mutant spermatids. Instead, observations suggest a more subtle impairment of ODF1-dependent architecture, potentially through altered interaction with specific binding partners. ODF1 binds the ODFs to the axoneme and the mitochondrial sheath by interacting with SPAG4 and kinesin light chain 3 (KLC3), respectively^40,68^. Depletion of SPAG4 results in the partial disassembly of the axonemal complex as observed in animals expressing a catalytically dead TENT5C^69^. Overexpression of a KLC3 mutant unable to interact with ODF1 results in midpiece abnormalities^68^. In addition to *Odf1*, *Smcp* emerged as a putative target of TENT5C poly(A) polymerase activity in ES. *Smcp* encodes a protein closely associated with the mitochondrial capsules of mouse spermatozoa^70^. Although *Smcp-*null sperm display largely normal ultrastructure, they exhibit reduced motility leading to infertility^58^. While *Smcp* poly(A)-tail shortening and transcript dysregulation are unlikely to be sufficient to recapitulate the effects of TENT5C deficiency, we cannot exclude that they may contribute along *Odf1* to the mitochondrial disorganization observed in *Tent5c*-mutant mice.

In summary, our study comprehensively documents the transcriptome-wide changes in poly(A) tails during spermiogenesis. Consistent with previous observations made on a few transcripts, a global increase in transcript poly(A) tail length in the transition from spermatocytes to spermatids is followed by a gradual shortening of poly(A) tails during spermatid elongation^1–3,10–12^. While most mRNAs exhibit similar trends, each transcript displays unique variations in poly(A) profile reflective of specific mRNA stability and translation efficiency during spermiogenesis. In addition, our study shows the critical contribution of the poly(A) polymerase activity of TENT5C to support fertility by targeting a few transcripts in a cell-type- and organelle-specific manner. The deadenylation of *Odf1* affects the spatio-temporal distribution of the ODF1 protein required for flagellum morphogenesis, explaining how TENT5C polymerase activity supports sperm development and fertility. Given that the etiology of male infertility cannot always be determined by genetic variations or differences in RNA accumulation, our findings show that poly(A) tail length profiling can be used as a powerful tool to screen changes in transcript metabolism causative of sperm abnormalities.

## Methods

### Mouse models

Animal studies were performed in accordance with the Guide for the Care and Use of Laboratory Animals from the National Institutes of Health. Animal protocols were approved by the Institutional Animal Care and Use Committee from the National Institute of Environmental Health Sciences (ASP protocol #019-0004). Sex was not considered in the study design as findings apply to males only. The following mouse strains were maintained to generate experimental animals: C57BL/6J (The Jackson Laboratory, IMSR_JAX:000664) for backcross and fertility test; Tg(CMV-Cre) (B6.C-Tg(CMV-cre)1Cgn/J) (The Jackson Laboratory, IMSR_JAX:006054) for global *null* conversion of *floxed* alleles^71^; *Tent5c* C-terminal-GFP knock-in, *Tent5c*^*gfp/gfp*^ (C57BL/6-Tent5c<tm1(EGFP)Adki>)^32^; loxP-flanked *Tent5c, Tent5c*^*fl/fl*^ (B6.Cg-Tent5c<tm1Mmo>) used to generate global *Tent5c*-knockout mice; *Tent5c*^*null/null*^ (B6.Cg-Tent5c<tm1.1Mmo>) and catalytically dead TENT5C, *Tent5c*^*dcat/dcat*^ (C57BL/6-Tent5c<tm2Adki>)^32^. Mice were socially housed at the animal facility of the National Institute of Environmental Health Sciences and maintained on a 12 h/12 h light/dark cycle at 50 ± 15% humidity and 20.5–23.9 °C with free access to food and water.

### Generation of transgenic mice

The *Tent5c* conditional null (“flox”) locus was generated by inserting loxP sites in intron 1–2 and the 3′ UTR, flanking the single coding exon of *Tent5c*. Two separate donor plasmids were used for each loxP site. The 5′ loxP site donor plasmid consisted of a 400 bp 5′ homology arm “chr3:100,473,608-100,474,007 (mm10)”, 430 bp 3′ homology arm “chr3:100,473,173-100,473,602 (mm10)”, and a genetic payload of 42 bp that included the loxP site and unique restriction enzyme sites (SphI, NsiI, and NdeI). The 3′ loxP site donor plasmid consisted of a 461 bp 5′ homology arm “chr3:100,471,978-100,472,438 (mm10)”, 379 bp 3′ homology arm “chr3:100,471,596-100,471,974 (mm10)”, and a genetic payload of 43 bp that included the loxP site and unique restriction enzyme sites (KpnI, PsiI, and SspI). Gene targeting was done in B6129F1 embryonic stem cells (G4; 129S6/SvEvTac x C57BL/6Ncr). Embryonic stem cells were lipofected with a 6:6:1:1 molar ratio of donor plasmids and two Cas9-Puro/sgRNA (5′ loxP TACTCTTGGTCGCAGCCGTG NGG and 3′ loxP TAGCACATGGGATCTGAGCT NGG) delivery plasmids (pSpCas9(BB)−2A-Puro (PX459) V2.0), a gift from Feng Zhang (Addgene plasmid #62988)^72^. Following transfection, the cells were exposed to 48 h of puromycin selection (0.9 µg/mL) followed by standard clonal expansion and screening. Clones were screened with 5′ and 3′ PCR screens external to the four homology arms to identify clones homozygous for the proper integration of both loxP sites: *Tent5c* 5′Scr (Fwd: CTTTTGGAAGTTTACCTGCCCG, Rev: CACAGAACCACATCTCTCACCA) and *Tent5c* 3′Scr (Fwd: GGATTTTGAGGAAGCCTTTGACC, Rev: GTTTCCTATTGACAATCACCGCC). Screening amplicons from targeted clones were fully sequenced to confirm homozygous insertion of each loxP site. Homozygous *Tent5c*-flox embryonic stem cells were microinjected into albino C57BL/6 J blastocysts for chimeric founder generation. The *Tent5c flox* allele was re-screened in F1 offspring of chimeric founders. The line was then crossed to C57BL/6J wild-type mice to establish and expand the colony. To confirm proper Cre-mediated recombination and generate global *Tent5c*-knockout mice *Tent5c*^*null/null*^, the *Tent5c flox* mouse line was crossed to Tg(CMV-Cre) mice^71^. The closest predicted Cas9 off-target site was a 3 bp mismatch located 65 Mb away in intergenic sequence, so no attempts were made to screen for linked Cas9-mediated mutations.

### Genotyping

For routine colony maintenance, genotyping was performed by Transnetyx (Memphis, TN, USA) using automated DNA isolation from ear punches, followed by real-time PCR. The genotype of experimental animals was confirmed using conventional PCR from tail DNA. Briefly, a 5 mm tail snip was digested at 56 °C in 200 µL proteinase K buffer until completely lysed. The DNA was precipitated with the addition of 200 µL of AL Buffer (DNeasy Blood & Tissue kit, Qiagen) and 200 µL of ethanol. The mixture was loaded onto a DNeasy Mini spin column, and the DNA captured in the filter was sequentially washed in AW1 and AW2 buffers prior to elution in 100 µL AE Buffer. DNA concentrations were determined by NanoDrop and adjusted to 10 ng/µL for polymerase chain reaction. PCR was performed using ReadyMix Taq PCR Reaction Mix (Sigma-Aldrich, P4600). 20 µL enzymatic mix was prepared according to the manufacturer’s protocol and 20 ng of DNA was added to the reaction for amplification. The primer sequences, number of cycles, annealing temperature, and extension time specific to each amplification are provided in Supplementary Table 1. For each reaction, water was used as a non-template control. When specified, the PCR products were digested with PfeI restriction enzymes (NEB) according to the manufacturer’s instructions. Amplicons were run together with DNA ladders on a 1.4% agarose gel with ethidium bromide. Imaging was acquired using the Amersham ImageQuant800 v1.2.0 imaging system (Cytiva).

### Testicular single-cell suspensions

Testes isolated from 8 to 12 week-old male mice were decapsulated and placed in 25 mL of a freshly prepared Enriched Krebs-Ringer bicarbonate (EKRB) medium maintained at room temperature (120.1 mM NaCl, 4.8 mM KCl, 25.2 mM NaHCO₃, 1.2 mM KH₂PO₄, 1.2 mM MgSO₄·7H₂O, 11 mM Glucose, 1.3 mM CaCl_2_, 1× Pen/Strep (Sigma, P0781), 1× Essential AAs (Sigma, M71450), pH 7.4). Seminiferous tubules were dissociated from the interstitial tissue by Collagenase Type XI digestion (0.5 mg/mL, Sigma, C7657) in a 34 °C water bath for a minimum of 10 min with periodic tube inversion until tubules appeared dispersed. Tubules were then allowed to sediment for 2 min at room temperature and the interstitial cells were removed with the supernatant. Tubules were washed once with 25 mL of plain EKRB to remove residual collagenase and interstitial cells. The seminiferous tubules were further digested in 10 mL of EKRB supplemented with Trypsin-EDTA (0.005%, Gibco, 25200-056) in a 34 °C water bath for 15 min. After incubation, tubules were dissociated by pipetting 10–15 times with a serological pipet until a single-cell suspension was obtained. Trypsin activity was then stopped quickly by the addition of 1 mL of FBS (10% final concentration) and the solution of cells was centrifuged 500 × *g* for 5 min at RT to remove digestion media. The cell pellet was resuspended in 10 mL EKRB-10% FBS and the cell suspension was passed through a 0.45 µm nylon cell strainer to remove cell clumps. A 20 µL aliquot of cell suspension was removed for cell counting (Cellometer Auto T4 system v3.3.9.5, Nexcelom Bioscience). Cells were centrifuged at 500 × *g* for 5 min at RT and resuspended with EKRB-10% FBS at a concentration of 1 million cells/mL. Aliquots of 4 million cells were set aside as unstained and single-stain controls (Hoechst-only, PI-only). The rest of the cell suspension was sequentially stained with Hoechst 33342 (3.2 µg/mL, Invitrogen, H3570) at 34 °C for 20 min, and PI (1 µg/mL, Invitrogen, P3566) at 34 °C for an additional 10 min. Stained cells were immediately centrifuged at 500 × *g* for 5 min at 4 °C. A suspension of 13 million cells/mL in staining media was prepared and maintained at 4 °C until FACS sorting.

### Flow cytometric analysis and cell sorting

Cells were sorted according to their characteristic Hoechst fluorescence and light scattering using a BD FACSAria II cell sorter (Becton Dickinson Biosciences) equipped with a 70 µm nozzle and FACSDiVa v8.0.1 software (BDbiosciences). Initially, gates were set on a side-scatter (SSC-H vs SSC-W) followed by a forward scatter (FSC-H vs FSC-W) dot plot to isolate single cells. These cells were projected onto a forward scatter (FSC-A) versus side scatter (SSC-A) to identify the principal population of single cells free of debris. Dead cells were excluded using PI (Ex: 561; Em: 585) on a PE-A histogram. One hundred thousand viable cells were recorded for analysis. Viable cells stained with Hoechst were excited using a UV (Ex: 355) laser and were initially analyzed on a UV-A (605/40; 595 LP) versus UV-B (450/50) dot plot. Three distinct populations of cells were gated: Gate I for leptotene/zygotene spermatocytes (L/Z spermatocytes), Gate II for P/D spermatocytes, and Gate III for haploid spermatids. The Gate III population was subsequently examined on a FSC-A versus SSC-A dot plot to identify the Gate IIIa and IIIb populations corresponding to RS and ES, respectively. Cell were sorted and collected from Gates I, II, IIIa and IIIb. In some experiments, the gates were projected onto a FITC (Ex: 488; 525/50) histogram to capture GFP-positive cells. Cells were sorted into 1.5 mL Eppendorf tubes pre-coated with EKRB + 10% FBS. Collected cells were smeared on positively charged glass slides (Superfrost Plus, Thermo Scientific) for histological analyses. Dry pellets were snap-frozen after centrifugation at 500 × *g* for 25 min at 4 °C, for molecular analyses.

### RNA extraction

Frozen testes or germ cell pellets were homogenized in 1 mL of TRIzol and allowed to equilibrate at room temperature. Five minutes later, 200 µL of chloroform-isoamyl alcohol was added. The mixture was vigorously shaken, incubated for 2 min at room temperature, and centrifuged at 12,000 × *g* for 15 min at 4 °C. The aqueous fraction was moved to a new tube, and RNA was precipitated by adding 400 µL isopropanol and 0.5 µL of GlycoBlue to facilitate the visualization of the RNA pellet. After 15 min, the samples were centrifuged at 12,000 × *g* for 10 min at 4 °C. The RNA pellet was washed with 75% ethanol, then centrifuged at 7500 × *g* for 5 min at 4 °C. After ethanol removal, total RNA was resuspended in nuclease-free water and quantified using Qubit 4 Fluorometer APP2.10 + MCUv 0.27 (Qubit™ RNA HS Assay Kit, Thermo Fisher, Q32852). RNA integrity was confirmed on the Agilent TapeStation 4200, Controller Software 5.2 (Agilent Technologies) prior to further analysis.

### Poly(A) tail purification

For Direct RNA sequencing libraries, mRNAs were purified from total RNA using Dynabeads™ Oligo(dT)_25_ beads (Thermo Fisher). 20 µL of oligo(dT) beads per sample were washed with 40 µL of binding buffer (20 mM Tris-HCl, pH 7.5, 1.0 M LiCl, and 2 mM EDTA) and resuspended in 40 µL of binding buffer. 1–4 µg total RNA per sample were diluted to 40 µL in nuclease-free water and mixed with 40 µL of binding buffer, followed by denaturation at 65 °C for 2 min. The denatured RNA was mixed with 40 µL of washed oligo(dT) beads and incubated at room temperature for 5 min. Beads were captured on a magnetic rack and washed twice with wash buffer (10 mM Tris-HCl, pH 7.5, 0.15 M LiCl, and 1 mM EDTA). After the final wash, poly(A)-tailed mRNAs were eluted in 10 µL of elution buffer (10 mM Tris-HCl, pH 7.5) by incubating the beads at 80 °C for 2 min.

### Library multiplexing

For multiplexing of poly(A) tail libraries from different samples, we followed the same barcoded oligonucleotide strategy described by Smith and collaborators^73^. Briefly, four unique barcode sequences (BC1–4) were incorporated into the splint-ligation oligonucleotides; oligonucleotide sequences are listed in Supplementary Table 2. To create barcoded oligonucleotide duplexes, partially reverse-complementary oligonucleotides (final concentration of 1.4 µM each) were incubated together at 94 °C for 5 min in annealing buffer (0.01 M Tris-HCl, pH 7.5, 0.05 M NaCl) and gradually cooled to room temperature (0.1 °C/s). These barcoded oligonucleotide duplexes were used in the initial ligation step following the Oxford Nanopore Technologies Direct RNA sequencing protocol (ONT, SQK-RNA002). This barcoding strategy was used to prepare the following libraries: The P/D spermatocyte library multiplexed 3 mRNA extracts (BC 1–3), each representative of a pool of mRNAs from the P/D spermatocytes of 3 wild-type mice. The RS library multiplexed 3 mRNA extracts (BC 1–3), each representative of a pool of mRNAs from the RS of 3 wild-type mice. The busulfan libraries, replicate 1 and 2, were each multiplexing 4 mRNA extracts (BC 1–4) from 1 mouse per condition (*Tent5c*^*wt/wt*^ and *Tent5c*^*dcat/dcat*^, vehicle or busulfan-injected). Other libraries were designed to detect poly(A) tail length and terminal modifications. For this, we followed a splint-ligation-based strategy previously described in refs. ^73,74^. A barcoded duplex oligonucleotides mix was prepared by mixing different annealed barcoded oligonucleotide duplexes, each designed to capture RNA terminal molecules by identifying associated barcode sequences. Ten sets of annealed duplex oligonucleotides were generated by mixing complementary oligonucleotides (Supplementary Table 2) and incubating them at 94 °C for 5 min, followed by cooling to room temperature in the annealing buffer (0.01 M Tris-HCl, pH 7.5, 0.05 M NaCl). Then, a 100 nM oligonucleotide mix was prepared according to the ratios of A, U, and G capturing oligos listed in Supplementary Table 2. BC1 (Oligo 1 in the BC mix) was used to identify non-modified poly(A) tails and BC2 (Oligo 2 in the BC mix) was used to identify terminal mono-uridylation. BC3 (Oligos 3–6 in the BC mix) and BC4 (Oligos 7–10 in the BC mix) were used to identify oligo-uridylation and terminal guanylation, respectively. This barcoding strategy was used to prepare the RS and ES libraries from mutant mice and their corresponding wild-type. 2 to 3 libraries per genotype and cell type were generated. Each library was built from a pool of mRNAs isolated from the spermatids of 3 independent mice. Each library included four distinct barcodes (BC 1–4) to detect 3′ end residues as described above.

Direct RNA sequencing. Direct RNA sequencing libraries were prepared according to the ONT standard protocol (Direct RNA Sequencing Kit, SQK-RNA002), with modifications to incorporate barcoded oligonucleotides. To capture poly(A) tail length and terminal modifications, a splint-ligation method was used to ligate barcoded oligonucleotides to the 3′ ends of transcripts. For this, a splint-ligation reaction was set up in a 15 µL volume by adding 9 µL of poly(A)-enriched mRNA, 3 µL of ligation buffer (6% PEG8000 in 10× T4 DNA ligation buffer, NEB), 1.5 µL of T4 DNA ligase (NEB), 1 µL of 0.1 ng/µL RNA standards mix^16^, and 1.5 µL of annealed barcoded oligonucleotides (100 nM) for library multiplexing or 1.5 µL barcoded oligonucleotides duplex mix (100 nM) for poly(A) tail terminal modifications (Supplementary Table 2). The reaction was incubated for 10 min at room temperature to anneal barcoded oligonucleotides for multiplexing, or 60 min at room temperature to enhance ligation efficiency when using barcoded oligonucleotides for terminal modifications. To maximize sequencing throughput, the ligated RNA was reverse-transcribed into an RNA-cDNA hybrid according to the manufacturer’s instructions (Thermo Fisher Scientific). Briefly, 15 µL of splint-ligated RNA was mixed with 9 µL of nuclease-free water, 2 µL of dNTPs (10 mM), 8 µL of 5× first-strand buffer, 4 µL of 100 mM DTT, and 2 µL of SuperScript II reverse transcriptase (Thermo Fisher Scientific). The reaction mix was incubated at 50 °C for 50 min, followed by 10 min at 70 °C and cooling to 4 °C in a thermocycler. The resulting RNA-cDNA hybrid was purified using 72 µL of RNAClean XP beads (Beckman Coulter, A63987) according to the manufacturer’s instructions, washed with 150 µL of 70% ethanol, and eluted in 20 µL of nuclease-free water on a magnetic stand. For multiplexing, distinct barcodes were eluted together after 5 min at room temperature in 20 µL of nuclease-free water. The RNA sequencing adapters containing motor protein (RMX, supplied in the Direct RNA Sequencing Kit SQK-RNA002) were ligated to the ends of RNA-cDNA hybrids. Briefly, 8 µL of ligation buffer (6% PEG in 10× T4 DNA ligation buffer, NEB), 6 µL of RMX-RNA adapter, 3 µL of nuclease-free water, and 3 µL of T4 DNA ligase (NEB) were mixed with 20 µL of purified RNA-cDNA hybrid in a final reaction volume of 40 µL. The ligation reaction was incubated for 15 min at room temperature. Adapter-ligated RNA-cDNA hybrids were purified using 40 µL of AMPure beads (Beckman Coulter), washed twice with 150 µL of wash buffer (WSB from SQK-RNA002), and eluted from beads in 21 µL of elution buffer (EB from SQK-RNA002) after a 10-min incubation at room temperature. The libraries were prepared for loading onto ONT flow cells (R9) using the Flow Cell Priming Kit (EXP-FLP002) according to the manufacturer’s instructions. Briefly, 21 µL of adapter-ligated libraries were mixed with 37.5 µL of RNA running buffer (RRB from SQK-RNA002) and 17.5 µL of nuclease-free water. The R9 flow cell was primed by adding 800 µL of the FLT/FB mix (30 µL FLT in 1 mL FB tube, from EXP-FLP002) into the priming port, followed by an additional 200 µL after 5 min of incubation. Finally, 75 µL of the library was loaded through the SpotOn port and sequenced on a MinION or GridION instrument (ONT).

### Fertility test

To test for fertility, each male mice 8–12 weeks-old was housed with two virgin C57BL/6J female mice 6–8 weeks-old for 5 days. Females were monitored for pregnancy and housed individually for delivery. The number of pups per litter was recorded on the day of birth. For each male mouse tested, the number of pups per litter was averaged across the 2 females.

### Busulfan treatment

To significantly decrease the proportion of germ cells in mouse testes and enrich for somatic cells, a freshly prepared busulfan solution (2.5 mg/mL in a 1:1 mixture of dimethyl sulfoxide (DMSO) and distilled water), or the corresponding vehicle, was injected intraperitoneally into *Tent5c*^*wt/wt*^ or *Tent5c*^*dcat/dcat*^ adult male mice in a single dose of 20 mg/kg (0.2 mL for a 25 *g* mouse). Testes were harvested 4 weeks after injection to allow for an adequate depletion of germ cells before histological and molecular analyses^75^.

### Sperm isolation and count

Both epididymides were collected in a 20-mm round-bottom glass dish containing 500 µL of 34 °C EKRB buffer (120.1 mM NaCl, 4.8 mM KCl, 25.2 mM NaHCO₃, 1.2 mM KH₂PO₄, 1.2 mM MgSO₄·7H₂O, 11 mM Glucose, 1.3 mM CaCl_2_, 1× Pen/Strep (Sigma, P0781), 1× Essential AAs (Sigma, M71450), pH 7.4) supplemented with 10% FBS. The cauda epididymides were isolated and punctured four times with a 26-gauge needle. The dish was incubated at 34 °C for 10 min to allow the spermatozoa to swim out. The epididymal tissue was removed and the sperm suspension were transferred into a clean low-binding Eppendorf tube with an additional 500 µL of EKRB buffer for a total volume of 1 mL. The sperm suspension was centrifuged at 500 × *g* for 5 min with the brakes set to minimum to avoid cell damage. The sperm pellet was then resuspended in 500 µL PBS 1×. Droplets corresponding to 20 µL of sperm suspension were smeared on positively charged glass slides (Superfrost Plus; Thermo Scientific) for histological analyses. The original sperm suspension was diluted tenfold in PBS 1× and loaded onto a 0.1-mm-deep Malassez hemocytometer. Flagella, used as a proxy to quantify germ cell production, were counted manually under a brightfield microscope (Axio, Zeiss).

### Tissue and smear processing

For histological analysis, testes and epididymides were fixed in 4% paraformaldehyde (PFA) solution at 4 °C overnight and paraffin-embedded. 5-μm sections were mounted on positively charged glass slides (Superfrost Plus, Thermo Scientific), deparaffinized, and rehydrated prior to staining or immunohistochemistry. Spermatozoa and sorted cells were smeared on positively charged glass slides (Superfrost Plus, Thermo Scientific), allowed to dry overnight, fixed in 4% PFA solution for 15 min, and rehydrated. For whole-tubule squash preparations, mouse testes were dissected and the tunica albuginea were removed to release seminiferous tubules. Tubules were briefly rinsed in PBS and gently unfolded in a fixative solution (0.8% PFA, 0.1% Triton ×-100 in PBS) for 5 min at room temperature. Individual tubules were transferred onto a glass slide (Superfrost Plus, Thermo Scientific) in a drop of PBS. Excess liquid was removed from the slide, a coverslip was placed on top of the tubules, and gentle pressure was used to release cells. Slides were snap-frozen in liquid nitrogen, the coverslip was immediately removed with a razor blade, and slides were air-dried at room temperature for ~1 h before immunostaining.

### Conventional histological staining

For H&E staining, rehydrated tissue sections and spermatozoa smears were stained in Mayer’s hematoxylin for 4 min and washed for 5 min in running water to remove excess dye and allow bluing. Testis and epididymis sections were then stained in eosin solution for 7 s and immediately transferred to 70% ethanol. Spermatozoa smears were left in eosin for 2 min to obtain a better contrast of the flagella. For Periodic Acid-Schiff (PAS) staining, rehydrated testis sections were oxidized in 0.5% periodic acid solution for 5 min and rinsed 3 times in distilled water. Sections were then incubated for 15 min in Schiff’s reagent, washed with distilled water, counterstained in Mayer’s hematoxylin for 1 min and quickly transferred to running water. Sections were then dehydrated with rising ethanol concentrations, transferred into xylene and permanently mounted in a xylene-based mounting medium. Bright-field images were captured on a Zeiss AxioObserver Z1 microscope (Carl Zeiss Microscopy). Transmitted light was collected with a Zeiss AxioCam IC color camera. ZEN Black 2.3 and ZEN Blue 3.9.101.03000 software (Zeiss) were used for image acquisition and processing.

### Immunofluorescence

Rehydrated testis sections, spermatozoa, sorted cell smears and whole tubule squash preparations were treated for antigen retrieval in 250 mL of boiling antigen unmasking solution (Vector, H3300) for 15 min, allowed to cool down for 20 min, and washed in PBS 1× for 5 min. Cell membranes were permeabilized in a 0.4% triton ×-100 solution for 10 min before incubation in a blocking solution (PBS 1×, 0.4% triton ×-100, 1% BSA, 5% normal donkey serum) for 1 h at room temperature. Slides were then incubated overnight at 4 °C with primary antibodies diluted in PBS 1×–0.4% triton ×-100 (INSL3, 1/200, ThermoFisher, PA5-55921; ODF1, 1/200, Abcam, ab197029; ODF2, 1/200, Genetex, GTX114594; TSSK2 (1E12), 1/200, ThermoFisher, H00023617-M01; WDR64, 1/50, Invitrogen, PA5-49160) or no primary antibody (negative controls). To detect cell death, sections were further stained using the In Situ Cell Death Detection Kit (Roche, #11684817910) and incubated 1 h at 37 °C with TUNEL reaction mixture (50 μL of enzyme solution mixed into 450 μL Label solution just prior to use) following the manufacturer’s instructions. Slides were then washed 3 times in PBS 1×–0.4% triton ×-100 and incubated for 1 h at room temperature with secondary antibodies (1/1000), rhodamine-labeled peanut agglutinin to label acrosomes (PNA, 1/1000, Vector Laboratories, #RL-1072), and the Alexa Fluor® 647 anti-SYCP3 antibody (1/200, Abcam, #ab205847) to identify meiotic cells. After 3 washes in PBS 1×, the TrueView Autofluorescence Quenching Kit (Vector Laboratories, #SP-8400-15) was used to reduce cell autofluorescence. DAPI mounting medium was used to stain for nuclei (VECTASHIELD Vibrance® Antifade Mounting Medium with DAPI, Vector Laboratories, H-1800-10). Images were captured using the Zeiss LSM 780 UV confocal microscope. Tissue sections were scanned with a 2 μm interval Z-stack followed by maximum intensity projection. Sperm images were captured on a Zeiss LSM 980 confocal microscope with AiryScan. The laser power and gain settings were held constant for image comparison ZEN Black 2.3 and ZEN Blue 3.9.101.03000 software (Zeiss) were used for image acquisition and processing.

### Electron microscopy

Sperm were isolated from the cauda epididymides and pellets were fixed in Modified Karnovsky fixative at RT for approximately 2 h. Samples were rinsed with 0.1 M phosphate buffer, post-fixed in 1% osmium tetroxide in phosphate buffer, rinsed in distilled water, dehydrated through a graded ethanol series (70, 75, 95, 100%), and transitioned to acetone. The samples were then infiltrated with increasing concentrations of Polybed 812 resin (1:3 resin:acetone, 1:2 resin:acetone, 1:1 resin:acetone, then 100% resin), embedded in absolute resin, and placed in an oven at 60 °C for 48 h to polymerize. Once polymerized, blocks were trimmed and semithin cross-sections of the pellet approximately 0.5 µm thick were cut, mounted on glass slides, and stained with 1% toluidine blue O in 1% sodium borate. The sections were examined by light microscopy to identify the areas of interest. Each block was trimmed to the area of interest and thin-sectioned at approximately 70 nm, placed on a 200-mesh copper grid, and stained with 3% aqueous uranyl acetate and Reynolds lead citrate. Digital images were captured with an AMT 16-megapixel camera attached to a JEOL JEM-1400+ transmission electron microscope operating at an accelerating voltage of 80 kV.

### Mass spectrometry

Peptides from RS and ES samples (isolated from 5 *Tent5c*^*dcat/dcat*^ mice and 5 *Tent5c*^*wt/wt*^ controls) were prepared using the Thermo Fisher EasyPep Mini MS sample preparation kit following the manufacturer’s recommendations. Sample preparation and mass spectrometry were performed in 2 batches comprising 3 and 2 mice per genotype, respectively. Briefly, RS and ES pellets were lysed by the addition of 100 μL of Lysis Buffer and resuspension by up-and-down pipetting and two 30-s pulses in a sonicating water bath at 25 °C. Protein content was quantified using the Bradford Assay, then proteins were reduced, carbamidomethylated, alkylated, and digested with Lys-C and trypsin. After peptides were purified using spin columns, each of the samples were analyzed using LC-MS/MS on a Q Exactive Plus mass spectrometer (ThermoFisher Scientific) interfaced with a nanoAcquity UPLC system (Waters Corporation) equipped with a 75 μm × 200 mm HSS T3 C18 column (1.8 μm particle, Waters Corporation) and a Symmetry C18 trapping column (180 μm × 20 mm) with 5 μm particle size at a flow rate of 450 nL/min. The trapping column was positioned in-line of the analytical column and upstream of a micro-tee union, which was used both as a vent for trapping and as a liquid junction. Trapping was performed using the initial solvent composition. Approximately 3 μg of peptide digest were injected onto the trapping column. Peptides were eluted by using a linear gradient from 99% solvent A (0.1% formic acid in water (v/v)) and 1% solvent B (0.1% formic acid in acetonitrile (v/v)) to 40% solvent B over 100 min. For the mass spectrometry, a data dependent acquisition method was employed with a dynamic exclusion time of 15 s and exclusion of singly charged ions. The mass spectrometer was equipped with a NanoFlex source and a stainless-steel needle and was used in the positive ion mode. Instrument parameters were as follows: sheath gas, 0; auxiliary gas, 0; sweep gas, 0; spray voltage, 2.7 kV; capillary temperature, 275 °C; S-lens, 60; scan range (m/z) of 375 to 1500; 1.6 m/z isolation window; resolution: 70,000; automated gain control (AGC), 3 × 10^6^ ions; and a maximum IT of 100 ms. For the MS/MS scans: TopN: 10; resolution: 17500; AGC 5 × 10^4^; maximum IT of 50 ms; and an (N)CE: 27. Mass calibration was performed before data acquisition using the Pierce LTQ Velos Positive Ion Calibration mixture (ThermoFisher Scientific).

### RNA FISH

Single-molecule RNA FISH was performed using RNAscope Multiplex Fluorescent Reagent Kit v2 (ACD, #323100) following the manufacturer’s instructions with modifications. Testis sections were dehydrated and pretreated in 200 mL of boiling 1× Target Retrieval Reagent (ACD, #322000) for 15 min. For protein degradation, the sections were treated with RNAscope Protease III (ACD, #322381) for 30 min at 40 °C. The probes used were Mm-Odf1 (ACD, #555661), RNAscope 3-plex Positive Control Probe—mouse (ACD, #320881), and RNAscope 3-plex Negative Control Probe (ACD, #320871). Fluorescent signal was obtained using Opal 520 (Akoya Biosciences, #OP-001001) at a 1/2000 dilution with LS Multiplex TSA buffer (ACD, #322810). Nuclear staining was obtained using DAPI (Millipore Sigma, #5087410001) at a 1/1000 dilution with 1× Wash Buffer (ACD, #320058). The DAPI was left on the slides to incubate at room temperature for 3 min and then washed in 1× Wash Buffer for 2 min. Slides were maintained in 1× Wash Buffer until mounting with ProLong™ Gold Antifade Mountant (Invitrogen, #P36930) and covered with a glass coverslip (Corning, #2975-225). The slides were left in the dark for 30 min at room temperature, then stored at 4 °C in the dark. Images were captured using the Zeiss LSM 780 UV confocal microscope. Tissue sections were scanned with a 2 µm interval Z-stack followed by maximum intensity projection. The laser power and gain settings were held constant for image comparison. ZEN Black 2.3 and ZEN Blue 3.9.101.03000 software (Zeiss) were used for image acquisition and processing.

### Direct RNA sequencing analysis and poly(A) tail-length determination

Guppy v6.1.3 (Oxford Nanopore Technologies) was used for read basecalling. The reads were then aligned to the GENCODE vM17 (GRCm38.p6) genes using Minimap2 software v2.24-r1122, with the -x parameter set to map-ont^76^. The length of the poly(A) tail was determined using Nanopolish v0.14, a signal-level analysis tool for Oxford Nanopore direct RNA sequencing that estimates poly(A)-tail length per read from the raw current^29,77^. To run the Nanopolish poly(A) pipeline, reads were indexed with Nanopolish and mapped reads were sorted and indexed with SAMtools v1.7^78^. Reads were demultiplexed using Deeplexicon v1.2^73^. Only reads with poly(A) tail-quality score PASS and confidence interval above 0.85 were selected for further analysis. Mapped read counts per sample after quality check are reported in Supplementary Table 3. For further analysis, minimal pre-filtering was applied to retain the contigs with a minimum of 10 reads in at least 2 replicates in one of the conditions. The resulting files, including transcript ID, poly(A) tail-length, modifications, and replicate information for each read were used as input for poly(A) tail analysis. The mean poly(A) tail length of each contig in each of the replicates was calculated and compared between conditions. A two-tailed *t*-test was used to identify transcripts with poly(A) tails statistically shorter (−) or longer (+) between conditions (*p* ≤ 0.05). For each contig in each of the replicates, the number of reads with poly(A) tail length within 30–120 nt (reported as ~60-nt poly(A) tails) and 120–210 nt (reported as ~150-nt poly(A) tails) were quantified and expressed as a percentage relative to the total number of reads obtained from the same contig.

### Differential transcript accumulation analysis

The DESeq2 v1.44.0 package (Bioconductor 3.19) was used to analyze relative changes in transcript accumulation from our direct RNA sequencing experiments^79^. Briefly, a count matrix was obtained from direct RNA sequencing using the DESeqDataSetFromMatrix function and used as an input to build the DESeqDataSet file. The original DESeq2 functions were used for the standard differential expression analysis steps (normalization, dispersion estimation, and fold change estimation) as described in ref. ^79^. Result tables were generated using the function results, which extracts log2 fold changes, Wald test *p*-values, and adjusted *p*-values corrected for multiple testing using the Benjamini–Hochberg procedure. Result tables are provided in Supplementary Data 1. A significance threshold of *q* ≤ 0.05 was used for data interpretation. The plotPCA function was used to visualize the overall effect of experimental covariates on a PCA. Additionally, a publicly available single-cell transcriptomic dataset of mouse spermatogenesis (GSE accession: GSE104556) was processed using Seurat v4.1.3 to compare absolute transcript abundance between spermatocyte 2 (SC2)/RS1 and condensed spermatids (CS)/ES^28,80^. After normalization of raw counts using SCTransform, differential expression analysis was performed using MAST v1.24 with mitochondrial gene percentage included as a latent variable^81^. MAST fits a two-part hurdle model, and significance was assessed using two-sided likelihood-ratio tests comparing full and reduced models. *P*-values were adjusted for multiple comparisons using the Bonferroni procedure. Result tables are shown in Supplementary Data 2. To ensure that only well-represented genes were included in the analysis, while still accounting for in-type heterogeneity, only genes expressed in >30% of cells in the cell type in which they were considered upregulated were used for downstream analysis.

### Differential translation

Differential translation was determined using Xtail v1.1.5^82^ using publicly available data reporting read counts of ribosome profiling (Ribo-seq) and matched RNA sequencing (RNA-seq) libraries from adult mouse testes (ArrayExpress accession: E-MTAB-7247)^31^.

### Cell composition analysis

The count matrix generated by the DESeq2 workflow from direct RNA sequencing was used as input for cell composition analysis using the Cell Population Mapping (CPM) deconvolution algorithm included in the scBio v0.1.6 package^83^. The abundance of testicular cell types for each sequenced replicate was inferred by comparing expression profiles to a publicly available single-cell transcriptomic dataset of mouse spermatogenesis used here as a reference (GSE accession: GSE104556)^28^.

### INSL3 immunofluorescence quantification

Interstitial areas (6–10 per mouse) were isolated from images of testis sections stained for INSL3 and DAPI using the ROI function of the ZEN Blue v3.9.101.03000 software (Zeiss). Cropped ROI images were then imported into FIJI v1. The green component of the RGB image corresponding to the INSL3 channel was extracted and converted to greyscale using the duplicate channel function. The interstitial compartment was then manually isolated from adjacent tubules with the freehand drawing tool followed by the clear outside function. The isolated interstitial region was converted to a binary mask to identify the INSL3 signal using the setAutoThreshold tool with the “Default dark no-reset” option. Watershed was applied to segment the binary image, and the particles were analyzed using the “Analyze Particle” command with the following options: size = 0-Infinity circularity = 0.00-1.00 show = Masks display exclude summarize add. Particle areas corresponding to the INSL3 signal were normalized to the number of Leydig cells quantified manually from the DAPI channel.

### Mass spectrometry data analysis

Data were processed in Proteome Discoverer v2.5.0.400 (Thermo Fisher Scientific) with a workflow employing nodes for the Minora Feature Detector, Sequest HT, and Percolator. Data were searched against the mouse UniprotKB sequence database (2024_4 release) using Sequest HT with the following settings: trypsin specificity; allowance for two missed cleavages; 20 ppm precursor mass tolerance (MS1) and 0.5 Da fragment mass tolerance (MS2); static carbamidomethylation of cysteine; variable oxidation of methionine; and variable deamidation of asparagine and glutamine, minimum peptide length of 6 amino acids, FDR of 0.01 (high confidence) and FDR of 0.05 (medium confidence) were used for both peptide and protein identifications, and one unique peptide was required for protein identification. A consensus workflow containing Feature Mapper and Precursor Ion Quantifier allowed for label-free quantification. Normalized protein abundances were used for downstream analyses. Exploratory visualization and quality control included heatmaps (ComplexHeatmap v2.15.4) for clustering^84^, manual review for outliers, principal component analysis to assess sample dispersion, and MA plots to confirm that median centering aligned the bulk of signals near *y* = 0 across the dynamic range. All samples had MAD scaling factors <2 within their experimental group. Protein-level detection was defined as an abundance above the limit of detection in >2/3 of samples in at least one experimental group. A total of 3,201 proteins met this criterion and were retained. Abundances were log2-transformed and median-centered per sample using the subset of proteins observed in all samples. This normalization assumes that most proteins are not differentially abundant. Differential abundance testing was performed in R v4.3.1 using limma v3.56.2^85^. Variance moderation was adjusted post-hoc using DEqMS v1.18.0^86^, which accounts for protein-level uncertainty as a function of peptide-spectrum match counts. *p*-values were adjusted for multiple testing using the Benjamini–Hochberg method. Result tables are shown in Supplementary Data 4.

### ODF1 immunofluorescence quantification on testis sections

Individual tubules (at least 3 per stage per mouse) were selected from testis sections stained for ODF1, SYCP3, PNA, and DAPI using the ROI tool in ZEN Blue v3.9.101.03000 (Zeiss). Cropped ROI images were imported into FIJI v1, and the green channel corresponding to ODF1 was extracted from the RGB image. The signal associated with spermatids was manually isolated using the freehand selection tool, followed by Clear Outside and cropping. Masks were generated from the cropped ODF1 image after conversion to 8-bit using fixed intensity thresholds. To define the total ODF1-positive area, a mask was created using a threshold of 10–255. The thresholded mask was converted to ROI (via Analyze Particles; no size or circularity filters specified) and measured to obtain area and mean intensity. To quantify ODF1-positive particles, a second mask was generated using a threshold of 55–255, and particles were segmented with Analyze Particles (size = 0–Infinity; circularity = 0.50–1.00; show = Masks; add; clear; summarize). Particle ROIs were measured to obtain total particle area. For each tubule, total particle area was normalized to the total ODF1-positive area. Measurements were computed for each tubule and averaged per mouse within each condition.

### Spermatid immunofluorescence profiling and quantification

Images (at least 3 40× fields per stage per mouse) from whole-tubule squashes stained for ODF1 or ODF2, PNA, and DAPI were imported into FIJI v1. The channels corresponding to ODF1/ODF2 and DAPI were retained and merged. For each image, individual spermatids were manually selected by drawing ROIs including the head and proximal midpiece (at least 5 spermatids per image). Each ROI was processed independently: pixels outside the ROI were cleared, the ROI was cropped, and the cropped two-channel image was saved as a multi-channel TIFF for downstream processing. Each cropped spermatid ROI was then oriented to a common horizontal axis (head to the left, tail to the right) prior to signal profiling. Orientation was determined on the ODF1/ODF2 channel using the Directionality plugin (Fourier components method; 90 bins from −180° to 180°), and the resulting rotation angle was applied to both channels with identical rotation parameters and canvas expansion to preserve the full signal. After rotation, one-dimensional line intensity profiles were extracted for both ODF1/ODF2 and DAPI using the Plot Profile function and exported for downstream analysis in R. Profiles from different spermatids were aligned using a relative coordinate system. The DAPI profile was smoothed (rolling mean window = 5), and the position of the steepest decrease in DAPI intensity (maximum negative slope), corresponding to the base of the nucleus/head–tail transition, was defined as the reference point (coordinate 0). All coordinates within each ROI were shifted to this reference, and subsequent analyses were restricted to a −5 to +5 window around coordinate 0. For each ROI, area under the curve (AUC) for the ODF1/ODF2 signal within this window was calculated using trapezoidal integration, then averaged per image and per mouse. To control for batch-to-batch variation, AUC values and intensity profiles were normalized by dividing by the mean AUC of a genotype-matched reference mouse processed in the same batch. Normalized profiles were averaged hierarchically (per image, then per mouse) at each relative coordinate and plotted by genotype with loess smoothing; the reference position (0) was indicated on all plots.

### Statistical analysis

Statistical details for each experiment are provided in the corresponding figure legend. To determine whether the data met the assumptions of the statistical tests, normality and homogeneity of variance were assessed using the Shapiro–Wilk and Levene tests, respectively. For non-parametric comparisons among more than two groups, a Kruskal–Wallis one-way analysis of variance applying the Bonferroni correction for multiple testing was used. When data met normality assumptions, parametric tests were used. To compare two unpaired samples with equal variance, an unpaired two-tailed Student’s *t*-test was used. To compare more than two groups with equal variance, statistical analyses were performed using a one-way ANOVA followed by Tukey’s multiple comparison test.

### Reporting summary

Further information on research design is available in the Nature Portfolio Reporting Summary linked to this article.

## Supplementary information

Supplementary Information

Peer Review file

Description of Additional Supplementary Files

Supplementary Data 1

Supplementary Data 2

Supplementary Data 3

Supplementary Data 4

Reporting Summary

## Source data

Source Data

## Data Availability

All the sequencing data are publicly available as of the date of publication and have been deposited at the Gene Expression Omnibus (GEO)^87^ with the accession codes GSE290698, GSE290699, GSE290700. Mass spectrometry data are publicly available as of the date of publication and have been deposited to Massive with the accession code MSV000100875. Publicly available datasets used in this study: GENCODE vM17 (GRCm38.p6); UniProt release 2024_04; Single-cell transcriptomic dataset of mouse spermatogenesis (GSE accession code: GSE104556)^28^; Read counts of ribosome profiling (Ribo-seq) and matched RNA sequencing (RNA-seq) libraries from adult mouse testes (ArrayExpress accession code: E-MTAB-7247)^31^. Source data are provided with this paper.
