# Supplementary material for: TENT5C extends Odf1 poly(A) tail to sustain sperm morphogenesis and fertility
Source: Nat Commun. 2026 Apr 20;17:5421. doi: 10.1038/s41467-026-71953-4 (PMC13279820; doi:10.1038/s41467-026-71953-4)
Supplement: Supplementary file 1 — Supplementary Information [file 41467_2026_71953_MOESM1_ESM.pdf]

Supplementary Figure 1 (1/2)

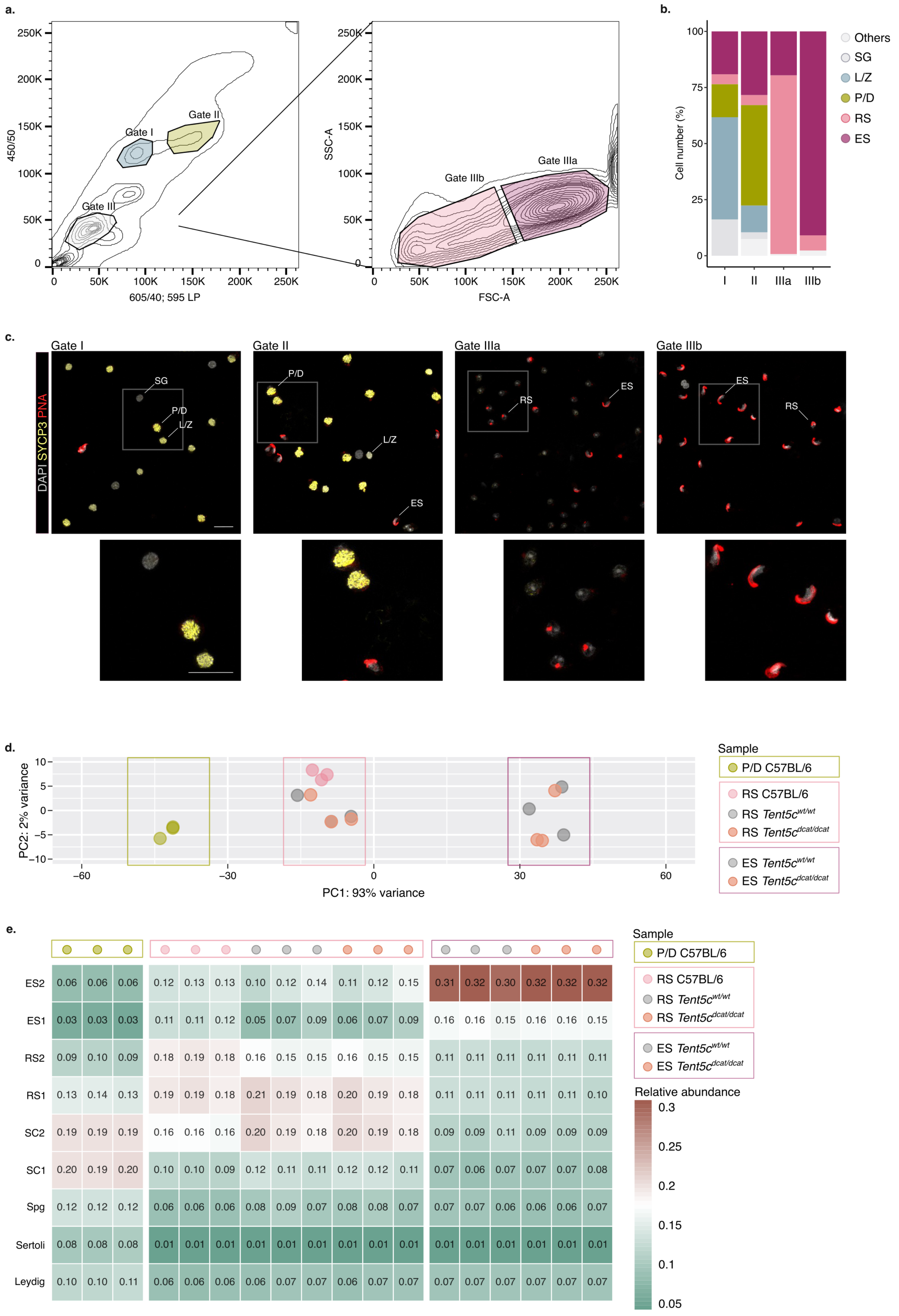

Supplementary Figure 1 (2/2)

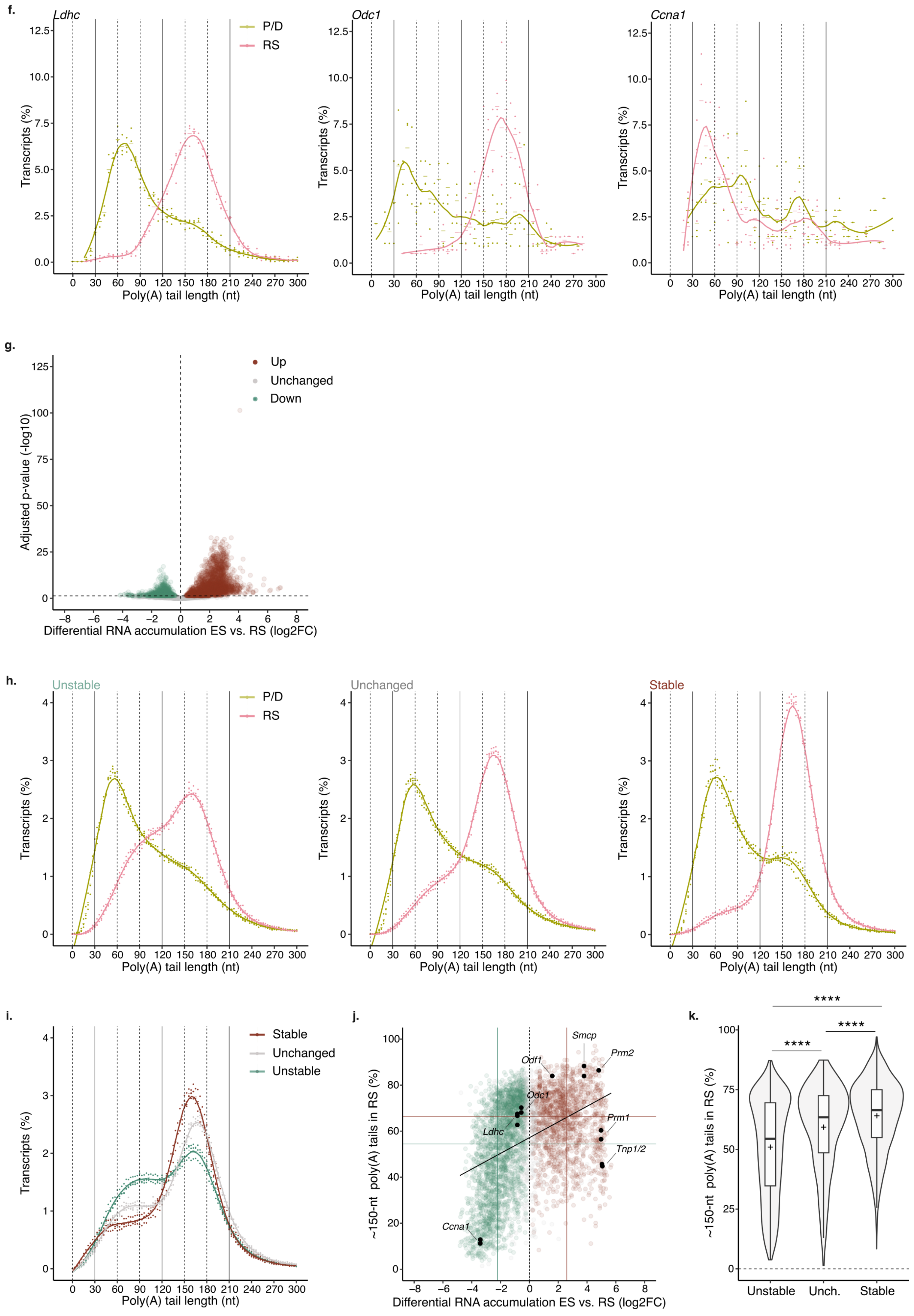

Supplementary Figure 1, related to Figure 1.

(a) Density plot representative of the gating strategy used in flow cytometry to isolate germ cell populations based on Hoechst fluorescence (left) and light scattering (right).
(b) Bar plot showing the proportion of spermatogenic cells in the isolated populations from Gate I, II, IIIa and IIIb. SG, spermatogonia; L/Z, leptotene/zygotene spermatocytes; P/D, pachytene/diplotene spermatocytes; RS, round spermatids; ES, elongated spermatids.
(c) Representative micrographs of the germ cell populations isolated by flow cytometry in Gate I, II, IIIa and IIIb. SYCP3 immunostaining (yellow) marks leptotene/zygotene spermatocytes (L/Z; punctate signal characteristic of unpaired synaptonemal complex) and pachytene/diplotene spermatocytes (P/D; elongated and intense signal indicating fully synapsed homologous chromosomes). PNA labeling (red) marks the acrosome apparatus of round spermatids (RS; punctate signal) and elongated spermatids (ES; elongated signal). DAPI labeling (grey) marks nuclei. Insets are highlighted in white. Scale, 20 µm.
(d) Principal Component Analysis (PCA) representation of the covariates for all sequenced spermatogenic cell populations (pachytene/diplotene spermatocytes, P/D; round spermatids, RS and elongated spermatids, ES) isolated from C57BL/6, *Tent5c*$^{wt/wt}$, and *Tent5c*$^{dcat/dcat}$ mice.
(e) Cell population mapping (CPM) showing the relative abundance of testicular cell types in all sequenced spermatogenic cell populations isolated from C57BL/6, *Tent5c*$^{wt/wt}$, and *Tent5c*$^{dcat/dcat}$ mice.
(f) Poly(A) tail length density plot for *Ldhc*, *Odc1*, and *Ccna1* in pachytene/diplotene spermatocytes (P/D, green) and round spermatids (RS, pink). Dots indicate values for individual biological replicates. The bars indicate the relative mean percentage of transcripts for each poly(A) tail length. The local polynomial regression fitting is shown as a solid line for each condition. nt: nucleotide.
(g) Volcano plot of differential RNA accumulation in elongated spermatids (ES) relative to round spermatids (RS). Upregulated (up), unchanged, and downregulated transcripts (down) are indicated in brown, grey and green, respectively. Wald test corrected by Benjamini and Hochberg for multiple testing. Significance threshold $q < 0.05$.
(h) Poly(A) tail length density plots as in (f) for pachytene/diplotene spermatocytes spermatocytes (P/D, green) and round spermatids (RS, pink) transcriptomes split by unstable (left), unchanged (middle) and stable (right) transcripts.
(i) Poly(A) tail length density plot as in (f) for stable, unchanged, and unstable transcripts in round spermatids (RS) shown in brown, grey and green, respectively. Transcript stability through spermiogenesis is here defined by absolute changes in RNA accumulation analysis between elongated spermatids (ES) and RS-matched cell clusters from a published whole-testis single-cell RNA-seq dataset [13].
(j) Scatter plot comparing fold change in absolute transcript accumulation between elongated spermatids (ES) and round spermatids (RS)-matched cell clusters from a published whole-testis single-cell RNA-seq dataset [13] to the percentage of reads per transcript with poly(A) tail ~150-nt long in RS. Each dot represents an individual transcript. Stable, unchanged, and unstable transcripts are indicated in brown, grey and green, respectively. Solid lines indicate the medians of both variables for stable and unstable mRNAs in brown and green, respectively. The linear fit is shown in black. Specific transcripts are indicated in black.
(k) Violin plot showing the proportion of reads per transcript with poly(A) tails ~150-nt long in round spermatids (RS). Transcripts are grouped by stability assessed by absolute changes in RNA accumulation between elongated spermatids (ES) and RS-matched cell clusters from a published whole-testis single-cell RNA-seq dataset [13]. The width of violins show the density of individual transcripts. The overlaid box plots display means as crosses and medians as lines; the boxes indicate the first and third quartiles and the bars indicate the 10th and 90th percentiles. Kruskal-Wallis test using Bonferroni correction for mutilple testing. **** adj.$p < 0.0001$. Unstable vs Unchanged (adj.p = 8.43 × 10^-17), Unstable vs Stable (adj.p = 2.08 × 10^-78), Unchanged vs Stable (adj.p = 1.52 × 10^-5).
For panels d-k, n = 3 replicates per condition; each replicate represents pooled mRNA extracted from 3 mice.
Source data are provided as a Source Data file.

Supplementary Figure 2

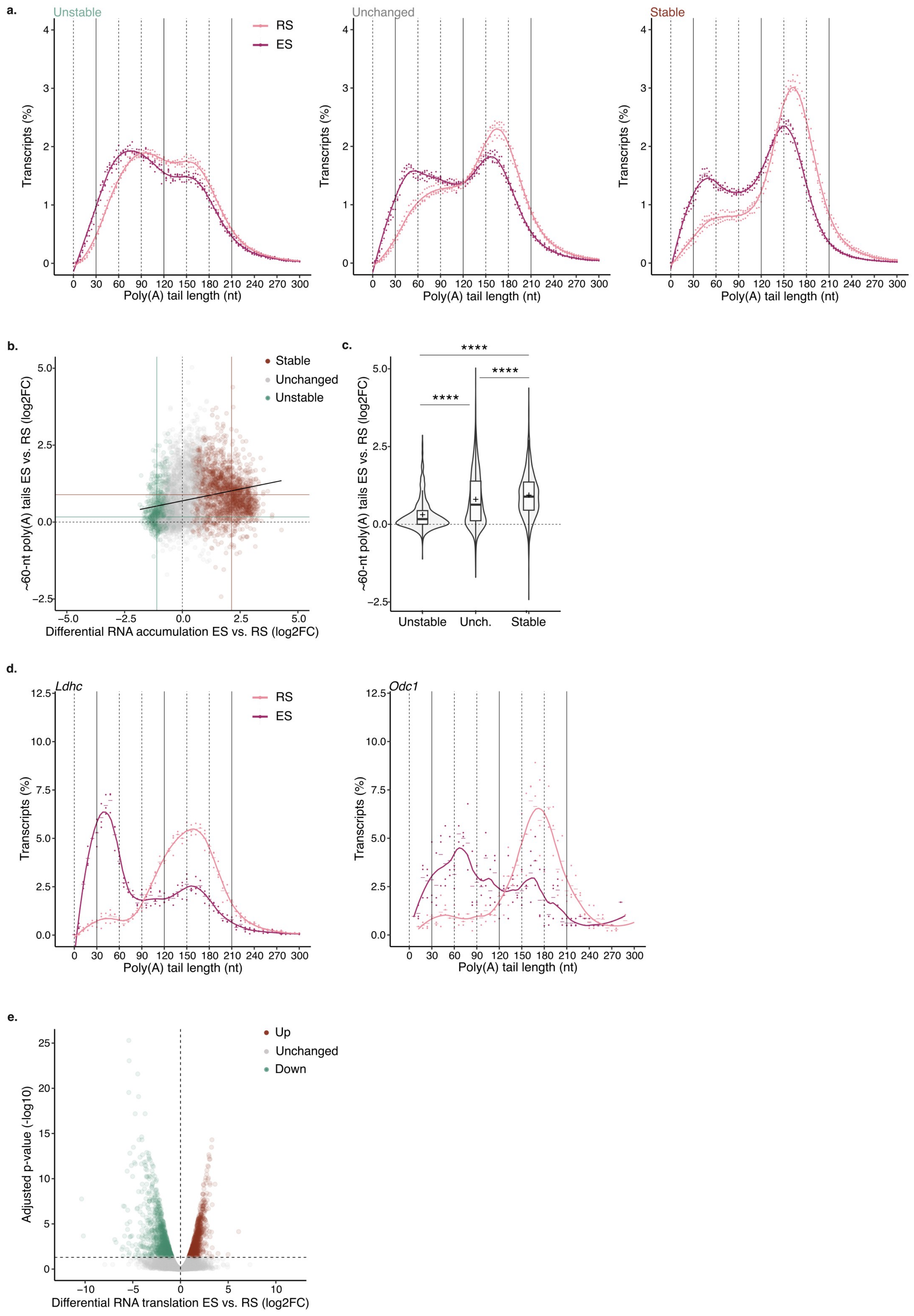

Supplementary Figure 2, related to Figure 2.

(a) Poly(A) tail length density plots for round spermatids (RS, pink) and elongated spermatids (ES, magenta) transcriptomes split by unstable (left), unchanged (middle) and stable (right) transcripts. Dots indicate values for individual biological replicates. The bars indicate the relative mean percentage for each poly(A) tail length. The local polynomial regression fitting is show as a solid line for each condition. nt: nucleotide.
(b) Scatter plot comparing fold change in transcript accumulation to the fold change in the proportion of reads per transcript with poly(A) tails ~60-nt long between round (RS) and elongated spermatids (ES). Each dot represents an individual transcript. Stable, unchanged, and unstable transcripts are indicated in brown, grey and green, respectively. Solid lines indicate the medians of both variables for upregulated and downregulated mRNAs in brown and green, respectively. The linear fit is shown in black.
(c) Violin plot showing the fold change in the proportion of reads per transcript with poly(A) tails ~60-nt long between round (RS) and elongated spermatids (ES) for transcripts grouped by stability. The width of violins show the density of individual transcripts. The overlaid box plots display means as crosses and medians as lines; the boxes indicate the first and third quartiles and the bars indicate the 10th and 90th percentiles. Kruskal-Wallis test using Bonferroni correction for mutilple testing. **** adj.$p < 0.0001$. Unstable vs Unchanged (adj.$p = 5.44 \times 10$^-69), Unstable vs Stable (adj.$p = 1.37 \times 10$^-125), and Unchanged vs Stable (adj.$p = 7.46 \times 10$^-19).
(d) Poly(A) tail length density plot as in (a) for *Ldhc* and *Odc1* in RS (pink) and ES (magenta).
(e) Volcano plot for the differential RNA translation in elongated spermatids (ES) relative to round spermatids (RS). Transcripts with increased (Up), unchanged or decreased (Down) level of translation are indicated in brown, grey and green respectively. Wald test corrected by Benjamini and Hochberg for multiple testing. Significance threshold $q < 0.05$.
For all plots, $n = 3$ replicates per condition; each replicate represents pooled mRNA extracted from 3 mice. Source data are provided as a Source Data file.

Supplementary Figure 3 (1/2)

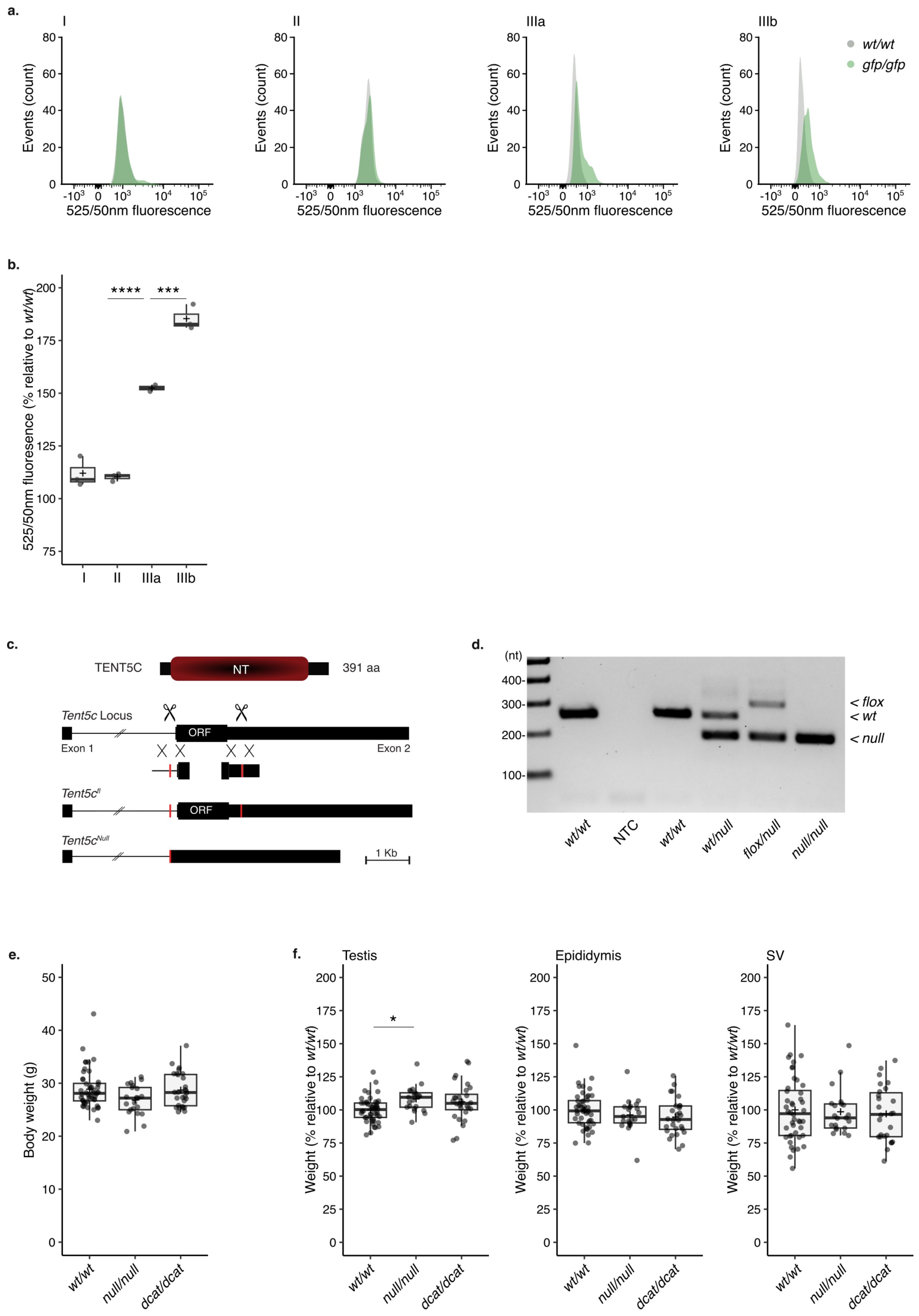

Supplementary Figure 3 (2/2)

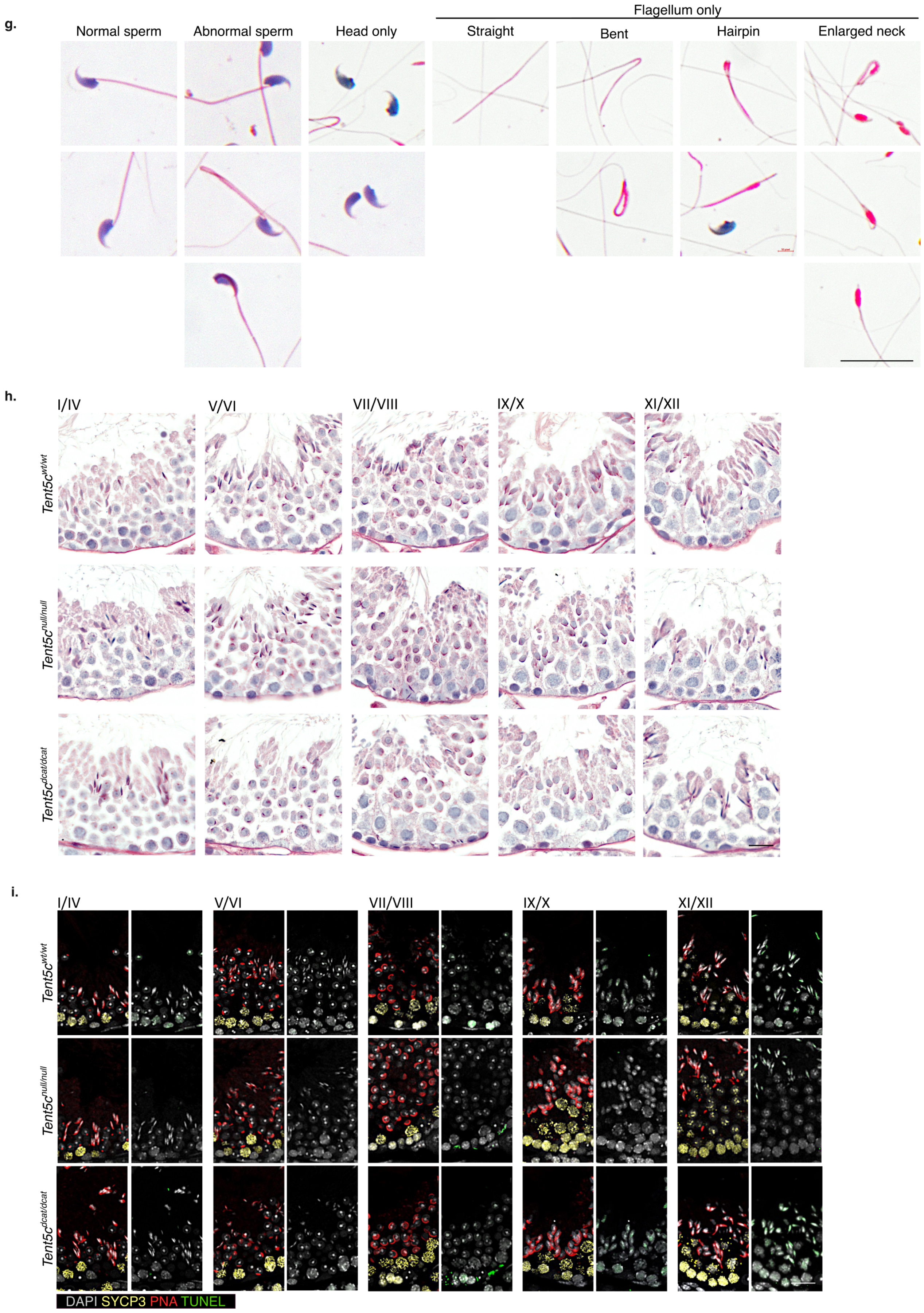

Supplementary Figure 3, related to Figure 3.

(a) Representative histograms of the intensity of 525/50 nm fluorescence in germ cell populations sorted from *Tent5c$^{wt/wt}$ and Tent5c$^{gfp/gfp}$* mice shown in grey and green respectively. *n* = 3 mice per condition.
(b) Box plot showing the intensity of 525/50 nm fluorescence in germ cell populations sorted from *Tent5c$^{gfp/gfp}$* mice. Data are expressed relative to *Tent5c$^{wt/wt}$* mice. Each dot represents one mouse. The crosses display the means; the lines show the medians; the boxes indicate the first and third quartiles and the bars indicate the 10th and 90th percentiles. Ordinary one-way analysis of variance (ANOVA), Tukey's mutilple comparison test. *** adj.$p < 0.001$, **** adj.$p < 0.0001$. IIIa vs I (adj.p = 3.33 × 10^-5), IIIb vs I (adj.p = 3.00 × 10^-7), IIIa vs II (adj.p = 2.41 × 10^-5), IIIb vs II (adj.p = 3.00 × 10^-7), IIIb vs IIIa (adj.p = 1.48 × 10^-4). II vs I not significant (adj.p = 9.67 × 10^-1). *n* = 3 mice per condition.
(c) Schematic of the targeting strategy used to develop the *Tent5c^{flox}* and *Tent5c^{null}* alleles.
(d) Representative gel images of the PCR amplification of the *Tent5c^{wt}*, *Tent5c^{flox}* and *Tent5c^{null}* alleles from DNA isolated from the tails of *Tent5c$^{wt/wt}$*, *Tent5c$^{wt/null}$*, *Tent5c$^{flox/null}$ and* , *Tent5c$^{null/null}$* mice. The molecular marker is shown on the left. nt: nucleotide; NTC: non template control.
(e) Box plot as in (b) showing the body weight of *Tent5c$^{wt/wt}$* (*n* = 47 mice)*, Tent5c$^{null/null}$* (*n* = 21 mice) and *Tent5c$^{dcat/dcat}$* adult mice (*n* = 28 mice) at sacrifice. Each dot represents one mouse.
(f) Box plots as in (b) showing testes, epididymides and seminal vesicle (VS) of *Tent5c$^{wt/wt}$, Tent5c$^{null/null}$* (*n* = 21 mice) and *Tent5c$^{dcat/dcat}$* adult mice (*n* = 28 mice). Organ weights are expressed relative to brain weight, and displayed as percentage relative to the average of the *Tent5c$^{wt/wt}$* controls (*n* = 47 mice). Kruskal-Wallis test using Bonferroni correction for mutilple testing. * adj.$p < 0.05$. For testes: *Tent5c$^{null/null}$* vs *Tent5c$^{wt/wt}$* (adj.p = 1.49 × 10^-2). *Tent5c$^{dcat/dcat}$* vs *Tent5c$^{wt/wt}$* not significant (adj.p = 5.93 × 10^-2), *Tent5c$^{dcat/dcat}$* vs *Tent5c$^{null/null}$* not significant (adj.p = 7.78 × 10^-1).
(g) Representative micrographs showing the range of germ cell morphologies observed in the semen from the caudal epididymides of *Tent5c$^{wt/wt}$, Tent5c$^{null/null}$* and *Tent5c$^{dcat/dcat}$* mice after Hematoxylin and Eosin (H&E) staining. Sperm heads (nuclei) show a blue staining and flagella shades of pink. Scale, 20 µm.
(h) Representative micrographs showing stage I to XII tubule cross-sections stained with Periodic Acid Schiff (PAS). Sections from *Tent5c$^{null/null}$* and *Tent5c$^{dcat/dcat}$* mice are compared to *Tent5c$^{wt/wt}$*. The stage VII/VIII tubule micrographs are also show in Figure 3E. Scale, 20 µm. *n* = 3 mice per condition.
(i) Representative micrographs as in (h) of stage I to XII tubule cross-sections. SYCP3 immunostaining (yellow) marks spermatocytes; PNA labeling (red) marks spermatid acrosomes, TUNEL immunostaining (green) marks apoptotic cells and DAPI labeling (grey) marks nuclei. The stage VII/VIII tubule micrographs are also show in Figure 3F. Scale, 20 µm. *n* = 3 mice per condition.
Source data are provided as a Source Data file.

Supplementary Figure 4 (1/2)

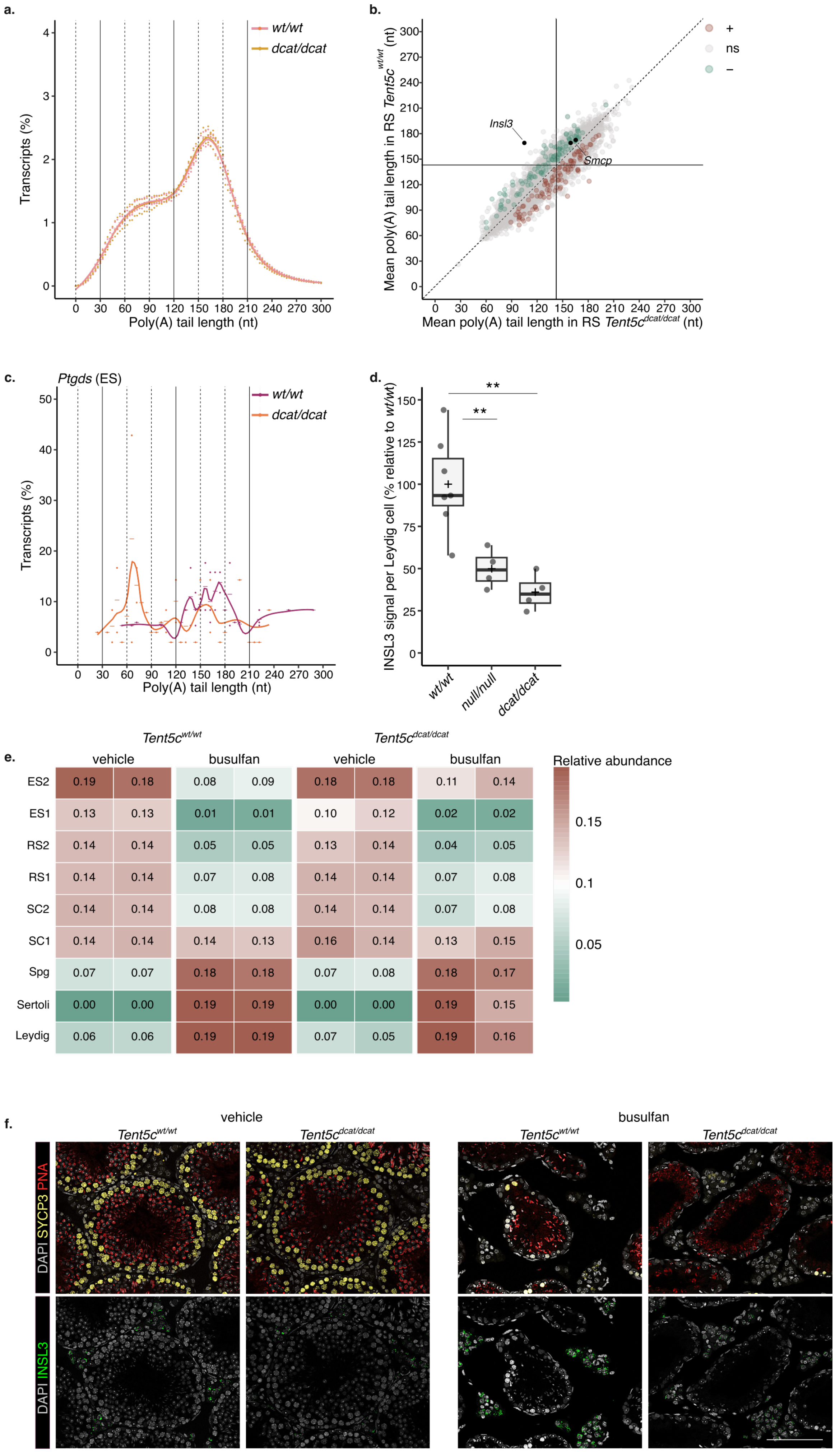

Supplementary Figure 4 (2/2)

g.

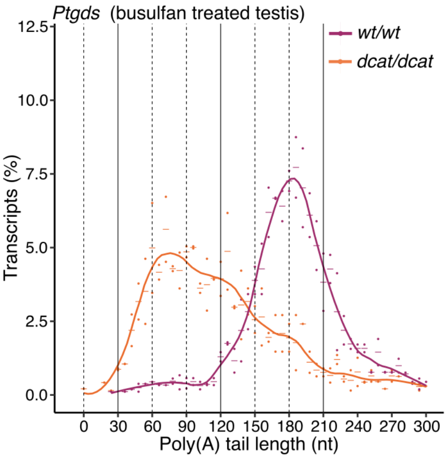

h.

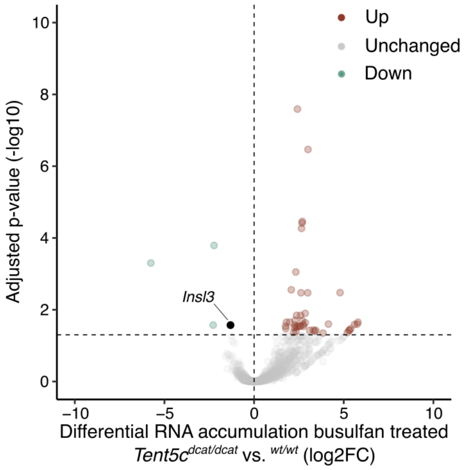

Supplementary Figure 4, related to Figure 4.

(a) Poly(A) tail length density plot of the round spermatid (RS) transcriptomes of *Tent5c$^{wt/wt}$* (pink) *and Tent5c$^{dcat/dcat}$* (yellow) mice. Dots indicate values for individual biological replicates. The bars indicate the relative mean percentage of transcripts for each poly(A) tail length. The local polynomial regression fitting is shown as a solid line for each condition. nt: nucleotide. *n* = 3 replicates per condition; each replicate represents pooled mRNA extracted from 3 mice.
(b) Scatter plot comparing the mean poly(A) tail length of each transcript in round spermatid (RS) of *Tent5c$^{wt/wt}$* and *Tent5c$^{dcat/dcat}$* mice. Each dot represents an individual transcript. Transcripts significantly increasing (+) or decreasing (-) in poly(A) tail length between genotypes are shown in brown or green, respectively; Student's t-test, two-tailed, significance threshold $p < 0.05$. ns: non-significant. *n* = 3 replicates per condition; each replicate represents pooled mRNA extracted from 3 mice.
(c) Poly(A) tail length density plot as in (a) for *Ptgds* transcripts from the elongated spermatid (ES) of *Tent5c$^{wt/wt}$* (magenta) *and Tent5c$^{dcat/dcat}$* (orange) mice. *n* = 3 replicates per condition; each replicate represents pooled mRNA extracted from 3 mice.
(d) Box plot showing the quantification of the INSL3 immunostaining per Leydig cells from testis cross-sections of *Tent5c$^{null/null}$* (*n* = 4 mice) and *Tent5c$^{dcat/dcat}$* mice (*n* = 4 mice), compared to *Tent5c$^{wt/wt}$* (*n* = 7 mice). Data are expressed relative to *Tent5c$^{wt/wt}$* mice. Each dot represents one mouse. The crosses display the means; the lines show the medians; the boxes indicate the first and third quartiles and the bars indicate the 10th and 90th percentiles. Ordinary one-way analysis of variance (ANOVA), Tukey's mutilple comparison test. ** adj.$p < 0.01$. *Tent5c$^{wt/wt}$* vs *Tent5c$^{null/null}$* (adj.p = 7.27 × 10^-3), *Tent5c$^{wt/wt}$* vs *Tent5c$^{dcat/dcat}$* (adj.p = 1.19 × 10^-3). *Tent5c$^{null/null}$* vs *Tent5c$^{dcat/dcat}$* not significant (adj.p = 6.38 × 10^-1).
(e) Cell population mapping (CPM) showing the relative abundance of testicular cell types in whole testes sequenced from *Tent5c$^{wt/wt}$* and *Tent5c$^{dcat/dcat}$* mice 4 weeks after vehicle or busulfan (20 mg/kg) injection. *n* = 2 mice per condition.
(f) Representative micrographs showing testis cross-sections from *Tent5c$^{wt/wt}$* and *Tent5c$^{dcat/dcat}$* mice 4 weeks after vehicle or busulfan (20 mg/kg) injection. INSL3 immunostaining in green; SYCP3 immunostaining (yellow) marks spermatocytes; PNA labeling (red) marks spermatid acrosomes, and DAPI labeling (grey) marks nuclei. Scale, 100 µm. *n* = 2 mice per condition.
(g) Poly(A) tail length density plot as in (a) for *Ptgds* transcripts from whole testes of *Tent5c$^{wt/wt}$* (magenta) *and Tent5c$^{dcat/dcat}$* (orange) busulfan-treated mice. *n* = 2 mice per condition.
(h) Volcano plot of differential RNA accumulation in the busulfan treated testes of *Tent5c$^{dcat/dcat}$* relative to *Tent5c$^{wt/wt}$* mice. Upregulated (up), unchanged, and downregulated transcripts (down) are indicated in brown, grey and green, respectively. *Insl3* transcript is indicated in black. Wald test corrected by Benjamini and Hochberg for multiple testing. Significance threshold $q < 0.05$. *n* = 2 mice per condition.
Source data are provided as a Source Data file.

Supplementary Figure 5 (1/3)

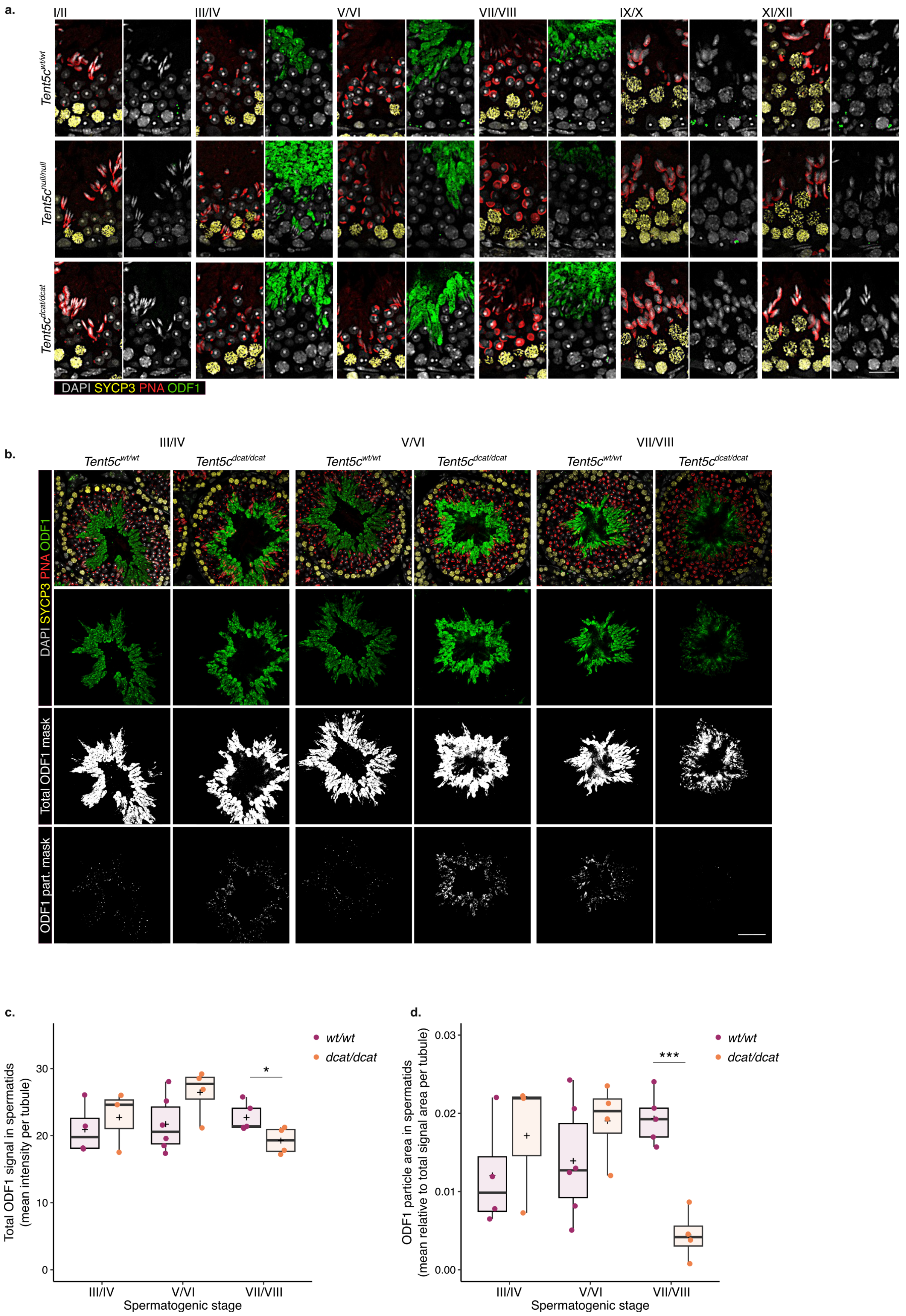

Supplementary Figure 5 (2/3)

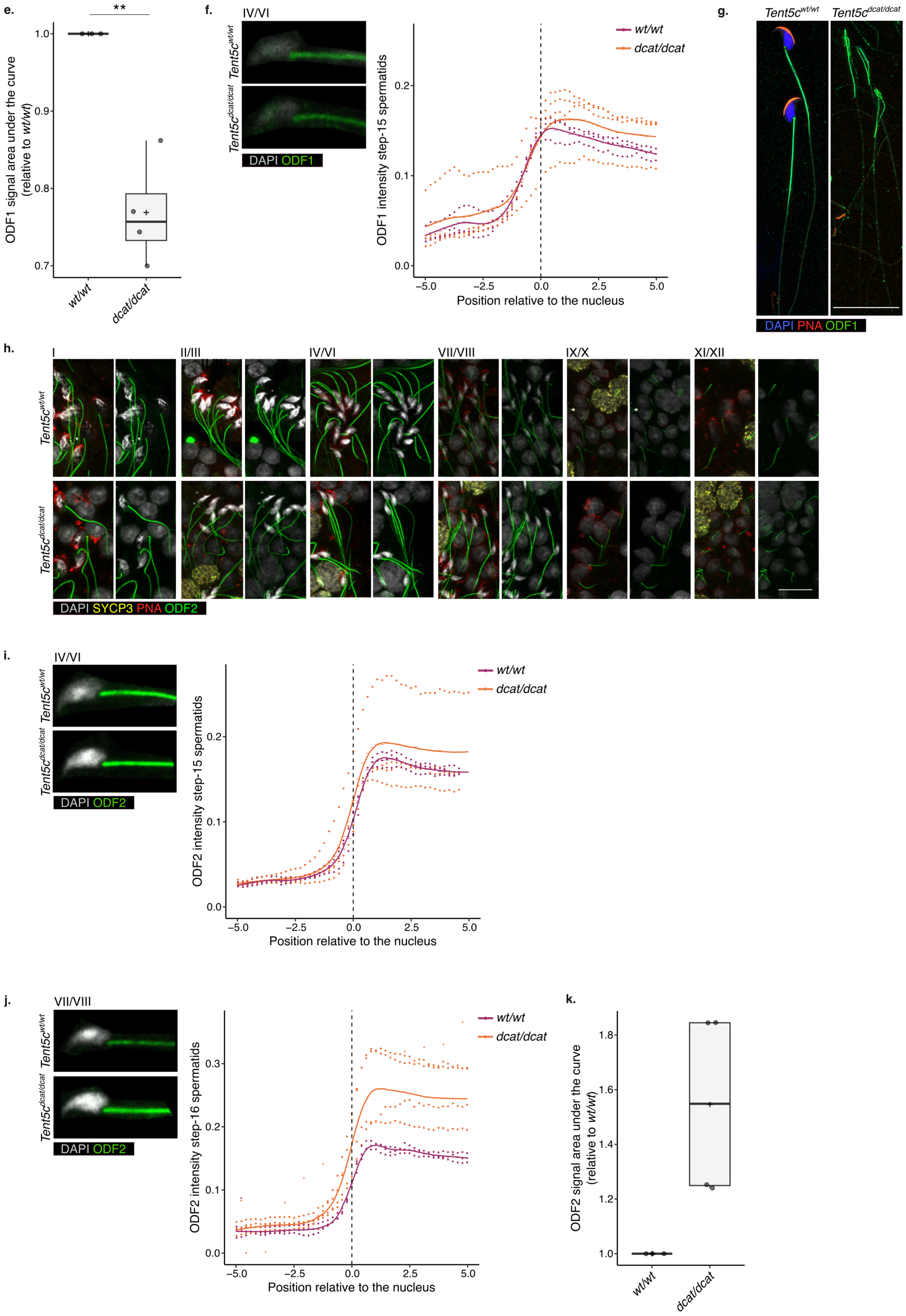

Supplementary Figure 5 (3/3)

l.

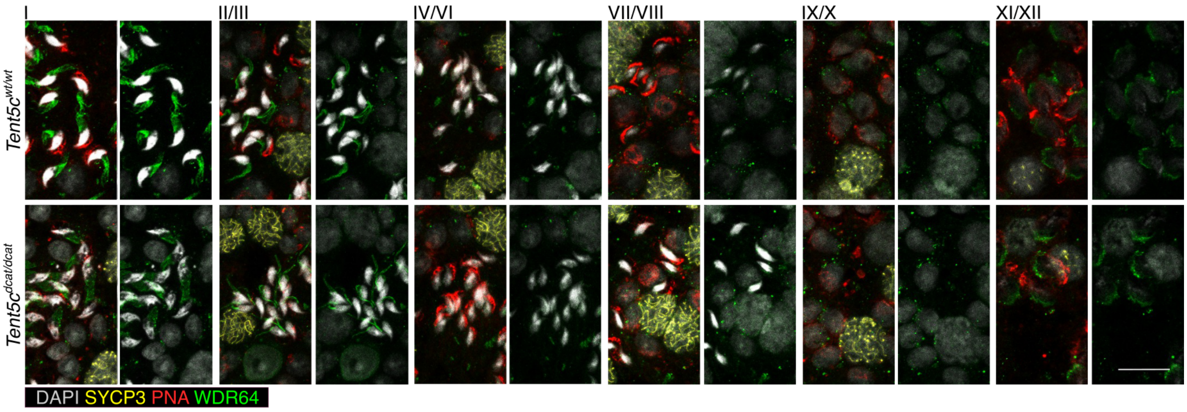

m.

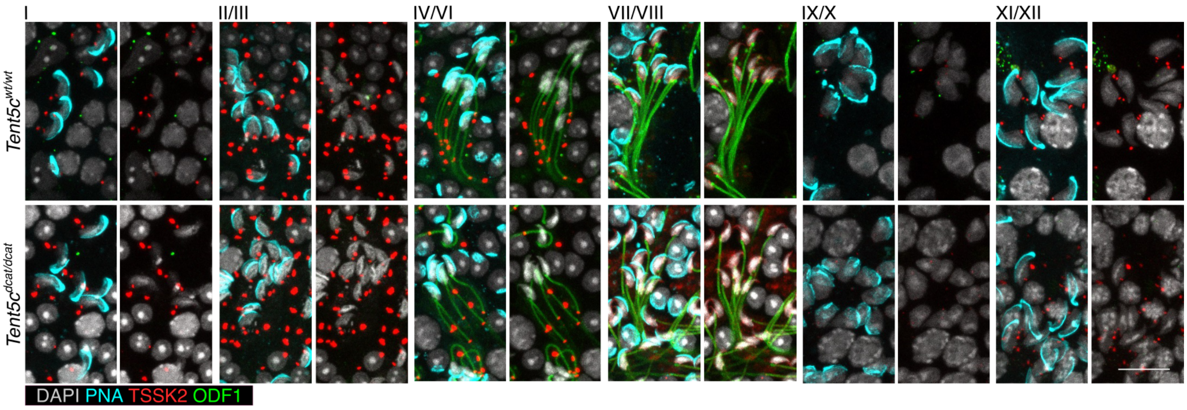

Supplementary Figure 5, related to Figure 6.

(a) Representative micrographs of stage I/II to XI/XII tubule cross-sections from *Tent5c*$^{null/null}$ (n = 2 mice) and *Tent5c*$^{dcat/dcat}$ mice (n = 4 mice) compared to *Tent5c*$^{wt/wt}$ (n = 3 mice). ODF1 immunostaining in green; SYCP3 immunostaining (yellow) marks spermatocytes; PNA labeling (red) marks spermatid acrosomes, and DAPI labeling (grey) marks nuclei. Scale, 20 µm.
(b) Representative micrographs as in (a) for stage III/IV, V/VI and VII/VIII tubule cross-sections from *Tent5c*$^{dcat/dcat}$ mice compared to *Tent5c*$^{wt/wt}$. The ODF1 signal associated with spermatids was isolated. Masks for total ODF1-positive area and ODF1-positive particles in white. Scale, 50 µm. n *Tent5c*$^{wt/wt}$/*Tent5c*$^{dcat/dcat}$ mice: 3/3 for stage III/IV, 6/4 for stage V/VI, and 4/4 for stage VII/VIII.
(c) Box plot showing the mean ODF1 immunostaining intensity in spermatids of stage III/IV, V/VI and VII/VIII tubules. *Tent5c*$^{dcat/dcat}$ mice are compared to *Tent5c*$^{wt/wt}$. Each dot represents one mouse. The crosses display the means; the lines show the medians; the boxes indicate the first and third quartiles and the bars indicate the 10th and 90th percentiles. Student's t-test, two-tailed. *$p < 0.05$. *Tent5c*$^{wt/wt}$ vs *Tent5c*$^{dcat/dcat}$ for stage III/IV (p = 6.09 × 10^-1), stage V/VI (p = 9.81 × 10^-2), and stage VII/VIII (p = 4.43 × 10^-2). n *Tent5c*$^{wt/wt}$/*Tent5c*$^{dcat/dcat}$ mice: 3/3 for stage III/IV, 6/4 for stage V/VI, and 4/4 for stage VII/VIII.
(d) Box plot as in (c) showing ODF1 particle area normalized to the total ODF1-positive area in spermatids of stage III/IV, V/VI and VII/VIII tubules. *Tent5c*$^{dcat/dcat}$ mice are compared to *Tent5c*$^{wt/wt}$. Student's t-test, two-tailed. ***$p < 0.001$. *Tent5c*$^{wt/wt}$ vs *Tent5c*$^{dcat/dcat}$ for stage III/IV (p = 4.50 × 10^-1), stage V/VI (p = 2.24 × 10^-1), and stage VII/VIII (p = 3.28 × 10^-4). n *Tent5c*$^{wt/wt}$/*Tent5c*$^{dcat/dcat}$ mice: 3/3 for stage III/IV, 6/4 for stage V/VI, and 4/4 for stage VII/VIII.
(e) Box plot as in (c) showing area under the curve from ODF1 line-intensity profiles of step-16 spermatids from whole-tubule squashes of *Tent5c*$^{dcat/dcat}$ mice (n = 4 mice) compared to *Tent5c*$^{wt/wt}$ (n = 3 mice). Data are expressed relative to *Tent5c*$^{wt/wt}$ mice. Student's t-test, two-tailed. **$p < 0.01$ (p = 0.006646).
(f) ODF1 line-intensity profiles of step-15 spermatids from whole-tubule squashes of *Tent5c*$^{dcat/dcat}$ mice (n = 4 mice) compared to *Tent5c*$^{wt/wt}$ (n = 3 mice). Individual spermatids were aligned to the base of the nucleus defined as coordinate 0. Dots indicate normalized intensity values for individual biological replicates. The bars indicate the mean ODF1 intensity for each position and solid lines show loess smoothing for each genotype.
(g) Representative micrographs of sperm from the cauda epididymides of *Tent5c*$^{dcat/dcat}$ mice compared to *Tent5c*$^{wt/wt}$. ODF1 immunostaining in green; PNA labeling (red) marks sperm acrosomes, and DAPI labeling (blue) marks nuclei. Scale, 20 µm. n = 2 mice per condition.
(h) Representative micrographs of stage I to XI/XII cells from whole squash tubules of *Tent5c*$^{dcat/dcat}$ mice (n = 4 mice) compared to *Tent5c*$^{wt/wt}$ (n = 3 mice). ODF2 immunostaining in green; SYCP3 immunostaining (yellow) marks spermatocytes; PNA labeling (red) marks spermatid acrosomes, and DAPI labeling (grey) marks nuclei. Scale, 20 µm.
(i) ODF2 line intensity profiles as in (f) for step-15 spermatids from whole-tubule squashes of *Tent5c*$^{dcat/dcat}$ mice (n = 4 mice) compared to *Tent5c*$^{wt/wt}$ (n = 3 mice).
(j) ODF2 line intensity profiles as in (f) for step-16 spermatids from whole-tubule squashes of *Tent5c*$^{dcat/dcat}$ mice (n = 4 mice) compared to *Tent5c*$^{wt/wt}$ (n = 3 mice).
(k) Box plot as in (c) showing area under the curve from ODF2 line intensity profiles of step-16 spermatids from whole-tubule squashes of *Tent5c*$^{dcat/dcat}$ mice (n = 4 mice) compared to *Tent5c*$^{wt/wt}$ (n = 3 mice). Data are expressed relative to *Tent5c*$^{wt/wt}$ mice. Student's t-test, two-tailed. p = 0.05097.
(l) Representative micrographs as in (h) on stage I to XI/XII cells from whole squash tubules of *Tent5c*$^{dcat/dcat}$ mice compared to *Tent5c*$^{wt/wt}$. WDR64 immunostaining in green; SYCP3 immunostaining (yellow) marks spermatocytes; PNA labeling (red) marks spermatid acrosomes, and DAPI labeling (grey) marks nuclei. Scale, 20 µm. n = 3 mice per condition.
(m) Representative micrographs as in (h) on stage I to XI/XII cells from whole squash tubules of *Tent5c*$^{dcat/dcat}$ mice compared to *Tent5c*$^{wt/wt}$. TSSK2 immunostaining in red; ODF1 immunostaining in green; PNA labeling (cyan) marks spermatid acrosomes, and DAPI labeling (grey) marks nuclei. Scale, 20 µm. n = 3 mice per condition.
Source data are provided as a Source Data file.

Supplementary Table 1 | Genotyping primers and PCR conditions for allele amplifcation.

Alleles	*Tent5c wt and gfp*	*Tent5c wt, f and null*	*Tent5c wt and dcat*
**Primers**	(F) CTTCAGAACCACTTCTCGGA	(F1) CCTGGCTGGGTATTACTCTTGG	(F) AGGTCCTGACTGAGGTCGTG
	(R) AGAAGTCACGCCTCCTATTG	(F2) CGTGAGCCAACCCTAAAAGG	(R) TTCCTCAAAATCCCCGTACA
		(R) GCTGAGAATCCTGAATGAGAGAGC	
**Number of cycles**	32	32	32
**Annealing temperature (°C)**	55	55	55
**Elongation time (sec)**	60	30	60
**Expected band size (bp)**	(wt) 532	(wt) 249	(wt Pfe1dig) 486 + 98
	(gfp) 1276	(f) 289	(dcat Pfe1dig) 292 + 194 + 98
		(null) 189	

Supplementary Table 2 | Splint oligos barcoded for Direct RNA Sequencing multiplexing

Barcoded oligos for sample multiplexing

Barcode ID	Oligo A	Oligo B
BC1	/5Phos/GGCTTCTTCTTGCTCTTAGGTAGTAGGTTC	GAGGCGAGCGGTCAATTTTCCTAAGAGCAAGAAGAAGCCTTTTTTTTTT
BC2	/5Phos/GTGATTCTCGTCTTTCTGCGTAGTAGGTTC	GAGGCGAGCGGTCAATTTTCGCAGAAAGACGAGAATCACTTTTTTTTTT
BC3	/5Phos/GTACTTTTCTCTTTGCGCGGTAGTAGGTTC	GAGGCGAGCGGTCAATTTTCCGCGCAAAGAGAAAAGTACTTTTTTTTTT
BC4	/5Phos/GGTCTTCGCTCGGTCTTATTTAGTAGGTTC	GAGGCGAGCGGTCAATTTTAATAAGACCGAGCGAAGACCTTTTTTTTTT

Barcoded oligos to detect terminal RNA modifcations

Oligo name	Oligo sequence	Barcode name	Amount
BC1_anneal_1	/5Phos/GGCTTCTTCTTGCTCTTAGGTAGTAGGTTC<br>GAGGCGAGCGGTCAATTTTCCTAAGAGCAAGAAGAAGCCTTTTTTTTT	Annealed oligo_1	90%
BC2_ anneal_2	/5Phos/GTGATTCTCGTCTTTCTGCGTAGTAGGTTC<br>GAGGCGAGCGGTCAATTTTCGCAGAAAGACGAGAATCACATTTTTTTTT	Annealed oligo_2	3%
BC3_ anneal_3	/5Phos/GTACTTTTCTCTTTGCGCGGTAGTAGGTTC<br>GAGGCGAGCGGTCAATTTTCCGCGCAAAGAGAAAAGTACAATTTTTTTT	Annealed oligo_3	2.40%
BC3_ anneal_4	/5Phos/GTACTTTTCTCTTTGCGCGGTAGTAGGTTC<br>GAGGCGAGCGGTCAATTTTCCGCGCAAAGAGAAAAGTACAAATTTTTTT	Annealed oligo_4	0.45%
BC3_ anneal_5	/5Phos/GTACTTTTCTCTTTGCGCGGTAGTAGGTTC<br>GAGGCGAGCGGTCAATTTTCCGCGCAAAGAGAAAAGTACAAAATTTTTT	Annealed oligo_5	0.08%
BC3_ anneal_6	/5Phos/GTACTTTTCTCTTTGCGCGGTAGTAGGTTC<br>GAGGCGAGCGGTCAATTTTCCGCGCAAAGAGAAAAGTACAAAAATTTTT	Annealed oligo_6	0.08%
BC4_ anneal_7	/5Phos/GGTCTTCGCTCGGTCTTATTTAGTAGGTTC<br>GAGGCGAGCGGTCAATTTTAATAAGACCGAGCGAAGACCCTTTTTTTTT	Annealed oligo_7	3%
BC4_ anneal_8	/5Phos/GGTCTTCGCTCGGTCTTATTTAGTAGGTTC<br>GAGGCGAGCGGTCAATTTTAATAAGACCGAGCGAAGACCTCTTTTTTTT	Annealed oligo_8	0.75%
BC4_ anneal_9	/5Phos/GGTCTTCGCTCGGTCTTATTTAGTAGGTTC<br>GAGGCGAGCGGTCAATTTTAATAAGACCGAGCGAAGACCTTCTTTTTTT	Annealed oligo_9	0.20%
BC4_ anneal_10	/5Phos/GGTCTTCGCTCGGTCTTATTTAGTAGGTTC<br>GAGGCGAGCGGTCAATTTTAATAAGACCGAGCGAAGACCTTTCTTTTTT	Annealed oligo_10	0.05%

Supplementary Table 3 | Direct RNA Sequencing read count obtained after quality check (QC)

Sample name	Read count after QC
pachytene_1	263890
pachytene_2	330400
pachytene_3	293310
round_1	338725
round_2	509157
round_3	381851
RS_TENT5c_WT_1	978889
RS_TENT5c_WT_2	708550
RS_TENT5c_WT_3	1455436
RS_TENT5c_dCAT_1	784068
RS_TENT5c_dCAT_2	647247
RS_TENT5c_dCAT_3	1030092
ES_TENT5c_WT_1	674834
ES_TENT5c_WT_2	742072
ES_TENT5c_WT_3	359278
ES_TENT5c_dCAT_1	603182
ES_TENT5c_dCAT_2	538070
ES_TENT5c_dCAT_3	768682
WT_veh_1	83105
WT_veh_2	119739
WT_bsf_1	101287
WT_bsf_2	179537
DCAT_veh_1	49267
DCAT_veh_2	217305
DCAT_bsf_1	96283
DCAT_bsf_2	154287
