# Supplementary material for: TENT5C extends Odf1 poly(A) tail to sustain sperm morphogenesis and fertility
Source: Nat Commun. 2026 Apr 20;17:5421. doi: 10.1038/s41467-026-71953-4 (PMC13279820; doi:10.1038/s41467-026-71953-4)
Supplement: Supplementary file 3 — Description of Additional Supplementary Files [file 41467_2026_71953_MOESM3_ESM.pdf]

Supplementary Data 1, differential RNA accumulation from direct RNA sequencing.

Output from DESeq2 v 1.44.0. (Bioconductor 3.19.; Love et al., 2014). Statistical significance was assessed using two-sided Wald tests, with multiple-comparison adjustment performed using the Benjamini-Hochberg procedure. A significance threshold of $q \leq 0.05$ was used for data interpretation.

Supplementary Data 2, differential RNA accumulation from single-cell RNA sequencing.

Differential transcript accumulation between elongated and round spermatids-matched cell clusters (CS/ES vs. SC2/RS2) from a published whole-testis single-cell RNA-seq dataset (Lukassen et al., 2018). Output from Seurat v4.1.3. analysis (Stuart et al., 2019). Significance was assessed using MAST v 1.24. (Finak et al., 2015) with two-sided likelihood-ratio tests comparing full and reduced models. P-values were adjusted for multiple comparisons using the Bonferroni procedure.

Supplementary Data 3, ES differential poly(A) tail length and changes in RNA abundance focused on HTCA/ODF transcripts.

Differential poly(A) tail length and relative change in RNA abundance between *Tent5c*$^{dcat/dcat}$ and *Tent5c*$^{wt/wt}$ elongated spermatids. The analysis focuses on transcripts encoding known, predicted, and candidate HTCA/ODF factors implicated in acephalic spermatozoa syndrome and male infertility (Budlak et al., 2025). The mean poly(A) tail length of each contig in each of the replicates was calculated and compared between conditions (delta_polya_nt<0, shorter tail in *Tent5c*$^{dcat/dcat}$; delta_polya_nt >0, longer tail in *Tent5c*$^{dcat/dcat}$). A two-tailed t-test was used to identify transcripts with differential poly(A) tail length ($p \leq 0.05$). Differential transcript accumulation from direct RNA sequencing was analyzed with DESeq2 v 1.44.0. (Bioconductor 3.19.; Love et al., 2014). Statistical significance was assessed using two-sided Wald tests, with multiple-comparison adjustment performed by the Benjamini-Hochberg procedure. A significance threshold of $q \leq 0.05$ was used for data interpretation.

Supplementary Data 4, differential protein accumulation by mass spectrometry.

Differential protein accumulation between *Tent5c*$^{dcat/dcat}$ and *Tent5c*$^{wt/wt}$ for round and elongated spermatids. Differential abundance testing was performed using limma v 3.56.2. (Ritchie et al., 2015). Variance moderation was adjusted post-hoc using DEqMS v 1.18.0. (Zhu et al., 2020), which accounts for protein-level uncertainty as a function of peptide-spectrum match counts. p-values were adjusted for multiple testing using the Benjamini–Hochberg method.
