# Supplementary material for: TENT5C extends Odf1 poly(A) tail to sustain sperm morphogenesis and fertility
Source: Nat Commun. 2026 Apr 20;17:5421. doi: 10.1038/s41467-026-71953-4 (PMC13279820; doi:10.1038/s41467-026-71953-4)
Supplement: Supplementary file 8 — Reporting Summary [file 41467_2026_71953_MOESM8_ESM.pdf]

nature portfolio

Corresponding author(s): Marcos Morgan

Last updated by author(s): Mar 27, 2026

Reporting Summary

Nature Portfolio wishes to improve the reproducibility of the work that we publish. This form provides structure for consistency and transparency in reporting. For further information on Nature Portfolio policies, see our Editorial Policies and the Editorial Policy Checklist.

Statistics

For all statistical analyses, confirm that the following items are present in the figure legend, table legend, main text, or Methods section.

n/a	Confirmed	
☐	☒	The exact sample size (n) for each experimental group/condition, given as a discrete number and unit of measurement
☐	☒	A statement on whether measurements were taken from distinct samples or whether the same sample was measured repeatedly
☐	☒	The statistical test(s) used AND whether they are one- or two-sided<br>*Only common tests should be described solely by name; describe more complex techniques in the Methods section.*
☒	☐	A description of all covariates tested
☐	☒	A description of any assumptions or corrections, such as tests of normality and adjustment for multiple comparisons
☐	☒	A full description of the statistical parameters including central tendency (e.g. means) or other basic estimates (e.g. regression coefficient) AND variation (e.g. standard deviation) or associated estimates of uncertainty (e.g. confidence intervals)
☐	☒	For null hypothesis testing, the test statistic (e.g. F, t, r) with confidence intervals, effect sizes, degrees of freedom and P value noted<br>*Give P values as exact values whenever suitable.*
☒	☐	For Bayesian analysis, information on the choice of priors and Markov chain Monte Carlo settings
☒	☐	For hierarchical and complex designs, identification of the appropriate level for tests and full reporting of outcomes
☒	☐	Estimates of effect sizes (e.g. Cohen's d, Pearson's r), indicating how they were calculated

Our web collection on statistics for biologists contains articles on many of the points above.

Software and code

Policy information about availability of computer code

Data collection	For mouse genotyping: Colony webpage (Transnetyx)<br>For gel imaging: Amersham ImageQuant800 v 1.2.0. (Cytiva)<br>For cell count: Cellometer Auto T4 system v 3.3.9.5. (Nexcelom Bioscience)<br>For FACS data acquisition: FACSDiVa v 8.0.1. (BDbiosciences)<br>For RNA concentration: Qubit 4 Fluorometer APP2.10 + MCUv 0.27 (Invitrogen)<br>For RNA quality: Agilent TapeStation 4200, Controller Software 5.2 (Agilent Technologies)<br>For direct RNA sequencing: MinKNOW software v24.11.16 (Noble; Oxford Nanopore Technologies)<br>For microscopy imaging: ZEN Black 2.3, ZEN Blue v 3.9.101.03000 (Zeiss)
Data analysis	For read base calling: Guppy v 6.1.3. (Oxford Nanopore Technologies)<br>For read alignment: Minimap2 v 2.24-r1122<br>For poly(A) tail length determination: Nanopolish v 0.14<br>For sorting and indexing reads: SAMtools v 1.7<br>For read demultiplexing: Deeplexicon v 1.2<br>For differential transcript accumulation analysis: DESeq2 v 1.44.0. (Bioconductor 3.19.), Seurat v 4.1.3., MAST v 1.24<br>For differential RNA translation: Xtail v 1.1.5.<br>For cell population mapping: scBio package v 0.1.6.<br>For general data processing and visualization: R-studio v 2022.12.0+353 (Posit Software, PBCc). Packages: dplyr v 1.1.4, tidyr v 1.3.1, readr v 2.1.5, ggplot2 v 3.5.1, rstatix v 0.7.2, stats v 4.4.1, biomaRt v 2.60.1, tidyverse v 2.0.0<br>For FACS data analysis: FlowJo v 10.9.0 (BDbiosciences)

For mass spectometry: Proteome Discoverer v 2.5.0.400 (Thermo Fisher Scientific), ComplexHeatmap v2.15.4, R v 4.3.1., limma v 3.56.2., DEqMS v 1.18.0.
For immunofluorescence quantification: FIJI v1
For figure: Inkscape 1.4.3 (0d15f75, 2025-12-25)

For manuscripts utilizing custom algorithms or software that are central to the research but not yet described in published literature, software must be made available to editors and reviewers. We strongly encourage code deposition in a community repository (e.g. GitHub). See the Nature Portfolio guidelines for submitting code & software for further information.

Data

Policy information about availability of data

All manuscripts must include a data availability statement. This statement should provide the following information, where applicable:

- Accession codes, unique identifiers, or web links for publicly available datasets
- A description of any restrictions on data availability
- For clinical datasets or third party data, please ensure that the statement adheres to our policy

All the sequencing data are publicly available as of the date of publication and have been deposited at the Gene Expression Omnibus (GEO) 87 with the accession codes GSE290698 [https://www.ncbi.nlm.nih.gov/geo/query/acc.cgi?acc=GSE290698], GSE290699 [https://www.ncbi.nlm.nih.gov/geo/query/acc.cgi?acc=GSE290699], GSE290700 [https://www.ncbi.nlm.nih.gov/geo/query/acc.cgi?acc=GSE290700]. Mass spectrometry data are publicly available as of the date of publication and have been deposited to Massive with the accession code MSV000100875 [https://massive.ucsd.edu/ProteoSAFe/dataset.jsp?task=0941328dad9f487a91579cdc037af1c0]. Publicly available datasets used in this study: GENCODE vM17 (GRCm38.p6) [https://www.gencodegenes.org/mouse/release_M17.html]; UniProt release 2024_04 [https://www.uniprot.org/release-notes/2024-07-24-release]; Single-cell transcriptomic dataset of mouse spermatogenesis (GSE accession code: GSE104556) [https://www.ncbi.nlm.nih.gov/geo/query/acc.cgi?acc=GSE104556]) 28; Read counts of ribosome profiling (Ribo-seq) and matched RNA sequencing (RNA-seq) libraries from adult mouse testes (ArrayExpress accession code: E-MTAB-7247) [https://www.ebi.ac.uk/biostudies/arrayexpress/studies/E-MTAB-7247] 31. Source data are provided with this paper.

Research involving human participants, their data, or biological material

Policy information about studies with human participants or human data. See also policy information about sex, gender (identity/presentation), and sexual orientation and race, ethnicity and racism.

Reporting on sex and gender	The study did not involve human participants.
Reporting on race, ethnicity, or other socially relevant groupings	The study did not involve human participants.
Population characteristics	The study did not involve human participants.
Recruitment	The study did not involve human participants.
Ethics oversight	The study did not involve human participants.

Note that full information on the approval of the study protocol must also be provided in the manuscript.

Field-specific reporting

Please select the one below that is the best fit for your research. If you are not sure, read the appropriate sections before making your selection.

☒ Life sciences ☐ Behavioural & social sciences ☐ Ecological, evolutionary & environmental sciences

For a reference copy of the document with all sections, see nature.com/documents/nr-reporting-summary-flat.pdf

Life sciences study design

All studies must disclose on these points even when the disclosure is negative.

Sample size	The sample size for each experiment performed in this study was determined based on the expected variability within the population, the size effect, and the desired statistical power to ensure robust and reliable results. In addition, the study complies with appropriate guidelines for the use of animals and accounts for practical constraints such as time and resources.
Data exclusions	No data exclusions were made in this study. The results reflect the full dataset without any omissions or biases introduced by selective exclusion of data points.
Replication	Where applicable, experiments were repeated multiple times using either identical or new sample sets, ensuring that all conditions were consistently included for relative comparison.<br>In certain cases, independent researchers conducted the repeat experiments.<br>Where applicable, positive and negative controls were incorporated to ensure proper calibration of each experimental repeat and to verify the reproducibility of the results.<br>Further details for each experimental component of the study are provided below.<br><br>Tissue collection and sperm isolation were performed independently across multiple sessions as aged-matched mice became available. Each

session included Tent5cwt/wt sibling controls processed in parallel with the corresponding experimental mutants.
Germ cell sorting, RNA isolation and Sequencing. Sequencing was performed in single runs of libraries multiplexed to include all replicates for each condition.
For the P/D-to-RS transition, each of the three biological replicates includes pooled mRNA from germ cells isolated from three mice, for a total of nine mice. Each mouse was processed in an independent flow cytometry experiment using a consistent workflow.
For the RS-to-ES transition in Tent5cwt/wt and dcat/dcat mice, each of the three biological replicates per genotype includes pooled mRNA from germ cells isolated from three mice, for a total of nine mice per genotype. One mouse of each genotype was included in each flow cytometry experiment, repeated across nine independent sessions using a consistent workflow.
All mRNA samples included in each of the sequencing experiments have been extracted in parallel.
Busulfan treatment and sequencing.
Busulfan experiment was performed in a single cohort comprising two mice per condition, for a total of eight mice (Tent5cwt/wt or dcat/dcat, vehicle- or busulfan-treated). This design was deemed sufficient because both the genotype-associated phenotype investigated here and the expected effect of busulfan on germ cell depletion have been extensively characterized previously. Consistent with this, only minor inter-animal variability was observed within each condition (Fig. 4e and Supplementary Fig. 4e–h), indicating that the conclusions were not driven by outlier samples or cohort-specific effects.
For sequencing associated with the busulfan experiment, each of the four conditions included two biological replicates, each corresponding to mRNA isolated from the testis of an independent mouse, for a total of eight mice. Two libraries were prepared and sequenced independently, each including one replicate from each of the four conditions.
All mRNA samples included in each of the sequencing experiments have been extracted in parallel.
Conventional histological stainings (H&E and PAS) were repeated at least twice using independent sample sets. In each case, control and experimental tissues were processed in parallel to ensure that the observed differences reflected biological variation rather than technical variability introduced during staining or sample handling.
Immunofluorescence experiments were repeated at least 3 times using independent sample sets, with control and experimental conditions processed in parallel in each experiment to ensure reproducibility and to minimize technical variation between staining runs. For INSL3 immunostaining, one repeat experiment was independently performed by a second researcher.
RNA FISH was repeated 3 times using independent sample sets, with control and experimental conditions processed in parallel in each experiment
Given the descriptive nature of electron microscopy, one sperm sample from a wild-type control and one from the littermate mutant were processed in parallel and analyzed to confirm ultrastructural features. Interpretation was based on reproducible observations across multiple sections and fields.
For mass spectrometry analysis of RS and ES samples, 5 mice per genotype were analyzed. Sample preparation and mass spectrometry were performed in 2 independent batches comprising 3 and 2 mice per genotype, respectively. Distributing samples across two independent batches provided technical replication of sample processing and data acquisition, while including all genotypes in each batch minimized batch-specific bias.

For all experiment, the exact sample size is provided in the legend.

Randomization

Mice were allocated to experimental groups based on their genotype. Litter mates from heterozygote matings were included in each group to ensure similar genetic background and age match between wild type and mutant mice. Mice from all experimental groups showed comparable body weight and baseline health status. Sex differences are not applicable to this study as only males were considered.
All the different germ cell types (spermatocytes, round spermatids, and elongated spermatids) were systematically isolated from the same mouse to only account for germline differentiation as a variable for comparison and minimize interindividual variability.

Blinding

Blinding was achieved by labeling mice with identification numbers that are only revealed after experimental procedure, data collection and analysis.

Reporting for specific materials, systems and methods

We require information from authors about some types of materials, experimental systems and methods used in many studies. Here, indicate whether each material, system or method listed is relevant to your study. If you are not sure if a list item applies to your research, read the appropriate section before selecting a response.

Materials & experimental systems

n/a	Involved in the study
☐	☒ Antibodies
☒	☐ Eukaryotic cell lines
☒	☐ Palaeontology and archaeology
☐	☒ Animals and other organisms
☒	☐ Clinical data
☒	☐ Dual use research of concern
☒	☐ Plants

Methods

n/a	Involved in the study
☒	☐ ChIP-seq
☐	☒ Flow cytometry
☒	☐ MRI-based neuroimaging

Antibodies

Antibodies used

INSL3, ThermoFisher, PA5-55921, Immunostaining dilution 1/200
ODF1, Abcam, ab197029, Immunostaining dilution 1/200
ODF2, Genetex, GTX114594, Immunostaining dilution 1/200

	TSSK2 (1E12), ThermoFisher, H00023617-M01, Immunostaining dilution 1/200<br>WDR64, Invitrogen, PA5-49160, Immunostaining dilution 1/50<br>Alexa Fluor® 647 Anti-SYCP antibody, Abcam, #ab205847, Immunostaining dilution 1/200
Validation	INSL3 (ThermoFisher, PA5-55921). This Antibody was verified by Relative expression to ensure that the antibody binds to the antigen stated (ThermoFisher statement). This same antibody was used in a peer reviewed study (Brouze et al. 2024).<br><br>ODF1 (Abcam, ab197029). This Antibody was used in a reference study to detect ODF1 localization by immunostaining during mouse spermiogenesis with a protocol similar to the one used in the present article (Zhang et al. 2024). This antibody was also used to detect ODF1 by western blot with a band corresponding to the expected ODF1 molecular weight (Abcam statement).<br><br>ODF2 (Genetex, GTX114594). This Antibody was verified by immunohistochemistery on mouse testis (GeneTex statement). The signal obtained in the present study is consistent with previous reports (Schalles et al. 1998).<br><br>TSSK2 (ThermoFisher, H00023617-M01). This Antibody was used in a reference study to detect TSSK2 localization by immunostaining during mouse spermiogenesis with a protocol similar to the one used in the present article (Lehti et al. 2025).<br><br>WDR64 (Invitrogen, PA5-49160). This Antibody was used in a reference study to detect WDR64 localization by immunostaining during mouse spermiogenesis with a protocol similar to the one used in the present article (Zhang et al. 2024). This antibody was also used to detect WDR64 by western blot with a band corresponding to the expected WDR64 molecular weight (Zhang et al. 2024).<br><br>All immunostaining include a "no primary antibody"; no signal was observed in any of the negative controls confirming the specific binding of the primary antibodies to their targets.

Animals and other research organisms

Policy information about studies involving animals; ARRIVE guidelines recommended for reporting animal research, and Sex and Gender in Research

Laboratory animals	The following mouse strains were maintained to generate experimental animals: C57BL/6J (The Jackson Laboratory, IMSR_JAX:000664) for backcross and fertility test; tg(CMV-Cre) (B6.C-Tg(CMV-cre)1Cgn/J) (The Jackson Laboratory, IMSR_JAX:006054) for global null conversion of floxed alleles 15; Tent5c C-terminal-GFP knock-in, Tent5cgfp/gfp (C57BL/6-Tent5c<tm1(EGFP)Adki>) 16; loxP-flanked Tent5c, Tent5cfl/fl (B6.Cg-Tent5c<tm1Mmo>) used to generate global Tent5c knockout mice; Tent5cnull/null (B6.Cg-Tent5c<tm1.1Mmo>) and catalytically dead TENT5C, Tent5cdcat/dcat (C57BL/6-Tent5c<tm2Adki>) 16. Male mice were euthanized between 8- to 12-week-old.
Wild animals	The study did not involve wild animals.
Reporting on sex	Sex was not considered in the study design as findings apply to males only.
Field-collected samples	The study did not involve samples collected from the field.
Ethics oversight	Animal studies were performed in accordance with the Guide for the Care and Use of Laboratory Animals from the National Institute of Health. Animal protocols were approved by the Institutional Animal Care and Use Committee from the National Institute of Environmental Health Sciences (ASP protocol #019-0004).

Note that full information on the approval of the study protocol must also be provided in the manuscript.

Plants

Seed stocks	The study did not involve plant samples.
Novel plant genotypes	The study did not involve plant samples.
Authentication	The study did not involve plant samples.

Flow Cytometry

Plots

Confirm that:

- [x] The axis labels state the marker and fluorochrome used (e.g. CD4-FITC).
- [x] The axis scales are clearly visible. Include numbers along axes only for bottom left plot of group (a 'group' is an analysis of identical markers).
- [x] All plots are contour plots with outliers or pseudocolor plots.
- [ ] A numerical value for number of cells or percentage (with statistics) is provided.

Methodology

Sample preparation	Testis isolated from 8-12 weeks male mice were decapsulated and collected in 25 mL of a freshly prepared Enriched Krebs-Ringer bicarbonate (EKRB) medium maintained at room temperature (120.1 mM NaCl, 4.8 mM KCl, 25.2 mM NaHCO3, 1.2 mM KH2PO4, 1.2 mM MgSO4.7H2O, 11 mM Glucose, 1.3 mM CaCl2, 1X Pen/Strep (Sigma, P0781), 1X Essential AA (Sigma, M71450), pH 7.4). Seminiferous tubules were dissociated from the interstitial tissue by Type XI Collagenase digestion (0.5 mg/ml, Sigma, C7657) in a 34 °C water bath for a minimum of 10 minutes with periodic tube inversion until tubules appeared dispersed. Tubules were then allowed to sediment for 2 minutes at room temperature and the interstitial cells were removed with the supernatant. Tubules were washed once with 25 mL of plain EKRB to remove residual collagenase and interstitial cells. The seminiferous tubules were further digested in 10 mL of EKRB supplemented with Trypsin-EDTA (0.005 %, Gibco, 25200-056) in a 34 °C water bath for 15 minutes. After incubation, tubules were dissociated by pipetting 10–15 times with a serological pipet until a single-cell suspension was obtained. Trypsin activity was then stopped quickly by the addition of 1 mL of FBS (10 % final concentration) and the solution of cells was centrifuged 500 g for 5 minutes at RT to clear out digestion media. The cell pellet was resuspended in 10 mL EKRB-10 % FBS and cell suspension was passed through a 0.45 uM nylon cell strainer to remove cell clumps. A 20 µL aliquot of cell suspension was removed for cell counting (Cellometer Auto T4 system, Nexcelom Bioscience). Cells were centrifuged at 500 g for 5 minutes at RT and resuspend with EKRB-10 % FBS at a concentration of 1 million cells /mL. Aliquots of 4 million cells were set aside to control for no staining, Hoechst 33342 staining only and PI staining only. The rest of the cell suspension was sequentially stained with Hoechst 33342 (3.2 ug/mL, Invitrogen, H3570) at 34 °C for 20 minutes, and PI (1 ug/mL, Invitrogen, P3566) at 34 °C for an additional 10 minutes. Stained cells were immediately centrifuged at 500g for 5 minutes at 4 °C. A suspension of 13 million cells/mL in staining media was prepared and maintained at 4 °C until FACS sorting.
Instrument	Cells were sorted according to their characteristic Hoechst fluorescence and light scattering using acBD FACSAriaII cell sorter (Becton Dickinson Biosciences) equipped with a 70-micron nozzle.
Software	For FACS data acquisition: FACSDiVa v 8.0.1. (BDbiosciences)<br>For FACS data analysis: FlowJo v 10.9.0 (BDbiosciences)
Cell population abundance	The purity of isolated populations was quantitatively determined by microscopy using fluorescent markers (Extended Data Fig. 1a to c). Late spermatocytes were identified with the meiotic marker synaptonemal complex protein 3 (SYCP), while acrosomal structures characteristic of RS and ES were stained with PNA. DAPI was used to stain DNA. About 50% of the cells from the sorting gate II were P/D spermatocytes, while the gates IIIa and IIIb were enriched with more than 80% of RS and ES, respectively (Extended Data Fig. 1b and c). Cell identity was further confirmed through direct RNA sequencing. Principal component analysis showed clustering of the populations according to their expected cell types (Extended Data Fig. 1d). We next mapped each sequenced transcriptome to cell clusters from a whole testis single-cell RNA sequencing dataset to computationally predict the cell type composition10. Each individual sample displayed a strong signature corresponding to the anticipated germ cell transcriptome (Extended Data Fig. 1e).
Gating strategy	Initially, gates were set on a side-scatter (SSC-H vs SSWC-W) followed by a forward scatter (FSC-H vs FSC-W) dot plot to isolate single cells. These cells were projected onto a forward scatter (FSC-A) versus side scatter (SSC-A) to identify the principal population of single cells free of debris. Dead cells were excluded using PI (Ex: 561; Em: 585) on a PI (PE-A) histogram. One hundred thousand viable cells were recorded for analysis. Viable cells stained with Hoechst excited using a UV (Ex: 355) laser were initially analyzed on a UV-A (605/40; 595 LP) versus UV-B (450/50) dot plot. Three distinct populations of cells were gated: Gate I for leptotene/zygotene spermatocytes (L/Z spermatocytes), Gate II for P/D spermatocytes and Gate III for haploid spermatids. The Gate III population was subsequently examined on a FSC-A versus SSC-A dot plot to identify the Gate IIIa and IIIb populations corresponding to RS and ES respectively. Cell sorting was performed capturing the populations from the Gate I, II, IIIa and b. In some experiments, the gates were projected onto a FITC (Ex: 488; 525/50) histogram to capture GFP positive cells.

- [x] Tick this box to confirm that a figure exemplifying the gating strategy is provided in the Supplementary Information.
