# Supplementary material for: TENT5C extends Odf1 poly(A) tail to sustain sperm morphogenesis and fertility
Source: Nat Commun. 2026 Apr 20;17:5421. doi: 10.1038/s41467-026-71953-4 (PMC13279820; doi:10.1038/s41467-026-71953-4)
Supplement: Supplementary file 2 — Peer Review file [file 41467_2026_71953_MOESM2_ESM.pdf]

nature portfolio

Peer Review File

TENT5C extends Odf1 poly(A) tail to sustain sperm morphogenesis and fertility

Corresponding Author: Dr Marcos Morgan

This file contains all reviewer reports in order by version, followed by all author rebuttals in order by version.

Version 0:

Reviewer comments:

Reviewer #1

(Remarks to the Author)
With this report, Dr. Baptissart and collaborators have investigated the function of the non-canonical cytoplasmic TENTC poly(A) polymerase in the regulation of gene expression during spermatogenesis. The authors have used two genetic mouse models of inactivation of TENTC: a knockout of the TENTC gene that prevents the expression of the protein, and a D90N and D92N mutations in the TENTC catalytic site that disrupts its catalytic activity. Both mouse mutants have the same phenotype of male sterility and production of headless spermatozoa. The authors use Oxford Nanopore Technologies (ONT) to sequence single RNA molecules and to quantify the poly(A) length. They found that the Poly(A) tail length increases at the transition from pachytene to round spermatids and then decreases from round spermatids to elongating spermatids in an mRNA-specific fashion. The authors conclude that during spermatid elongation, transcripts with 150nt poly(A)are stable and are translated. The gradual shortening of the details detected in ES follows translation and eventually leads to destabilization and decay. They then show that TENTC inactivation causes a decrease in poly(A) tail length in a small subset of mRNAs, the most affected transcripts being INSL3 and Ptgds. Given the lack of correlation between INSL3 poly(A) tail length and protein accumulation during spermatogenesis, they focus on ODF1 mRNA whose poly(A) tail is moderately affected by the loss of TENT5C function. Immunofluorescence analysis shows that ODF1 protein is still translated on a TENT5C null background but that protein localization in elongating spermatids and spermatozoa is disrupted. The authors conclude that TENT5C is required for spatial translation of the ODF1 mRNA.
That TENTC plays a critical function in spermatogenesis has already been reported in several publications and linked to the control of poly(A) tail length. However, the present study has the advantage of using isolated spermatogenic cells at different stages to measure the poly(A) length, providing a more accurate picture of the Poly(A) dynamics during spermiogenesis. In addition, the use of two different genetic models to study the impact of TENT5 loss of function adds strength to the study. The conclusion that poly(A) lengthening is associated with increased translation is a novel concept.
Fig. 1 By comparing poly(A) length and transcript levels, the authors establish a correlation between poly(A) length and stability of the mRNA. Can the authors better define the group of transcripts defined as “others” and provide some examples?
Line 301. The authors state that the poly(A) tails of Insl3 and Ptgds mRNAs are the most affected by the loss of TENT5C function. However, the authors do not mention whether this shortening of the poly(A) tail has any effect on transcript levels. Including this information would strengthen the conclusion that the length of the poly(A) tail correlates with mRNA stability.
Line 368. The authors mention ODF1 and Smcp as transcripts affected by the loss of TENT5C function. What about poly (A) tail and translation of the other components of the ODF or components of the connective piece? Is the polyadenylation of these other transcripts not affected?
Line 408. The authors note that the accumulation of the ODF1 protein may increase with the loss of TENT5C activity. This increase is in contrast with the observation that the loss of TENT5C function is associated with a moderate decrease in poly(A) length. A decrease in the length of the poly(A) tail should have affected the translation of the mRNA and the accumulation of the protein. Whether ODF1 protein is indeed increased in the TENT5C null is inferred from immunofluorescence measurements, which are difficult to evaluate even though the signal seems to be increased in the two mutants in Fig. 5C. Can ODF1 western blot be used to quantify the ODF1 protein in Wild type and TENT5C null mutants? Possibly this could be done after isolation of spermatogenic stages where the protein accumulates. The authors should then discuss how an increased accumulation of the proteins relates to decreased poly(A) length.
Line 411 ODF1 appears also to be mis-localized in spermatozoa in Fig. 5d. However, the authors do not report data on poly(A) tail length (see above) and protein localization accumulation for the other components of the outer dense fiber or of the connective piece. This information is necessary to determine whether only ODF1 mis-localization is the cause of the phenotype. On what basis do the authors not consider the possibility that ODF1 protein interactions are disrupted?

A previous report that the authors cite (PMID: 38909026) concluded that Tent5 controls polyadenylation of mRNAs with a leader sequence, thus affecting secretion of specific proteins. Can the authors confirm this prediction? Some of the mRNAs they consider affected do not seem to contain a leader sequence.
Line 556-557 Discussion. The authors conclude that ODF1 is mis-localized in the absence of TENT5C activity. However, this statement is difficult to evaluate with the data provided. Higher magnification data should be included. It is also not clear whether it is the ODF1 mRNA that is also mis-localized. Please discuss. The statement that TENT5C interacts with the manchette and centrioles and PLK4 is intriguing (see PMID: 32433990), but no experimental data are reported to show that in ES, TENT5C protein and ODF1 mRNA colocalize in these structures. Should the authors provide data of colocalization of TENT5C with centrioles in spermatids? Isolation of centrioles and showing that TENT5 C and ODF mRNA are enriched in this fraction would support the authors' conclusions.
The authors correctly state the genetic model used indicates that the catalytic activity of TENT5C is required during spermatogenesis. However, can the authors make predictions on the molecular complexes affected or not in the dcat/dcat mutant? All the interactions of TENT5C involving domains outside of the catalytic domain should be preserved in this genetic model. Would this imply that targeting requires the catalytic domain only?
Minor points
Line 395-397 The authors state that Prm2 poly(A) tail is not changed in the Tent5c dcat/dcat. Then they state that "Consistently, no change TNP2 protein expression were observed in the TENT5C -depleted spermatids." Should the authors show that TNP2 mRNA poly(A) tail is not changed in their genetic model?
Line 499 "For example, Prm2 transcripts encode for the last of the transition proteins and protamines to appear in late spermatids." This sentence is confusing

Reviewer #2

(Remarks to the Author)
In this study, Baptissart et al. examined the temporal dynamics of mRNA poly(A) tail length during spermatogenesis by performing direct RNA sequencing on FACS-sorted spermatogenic cells. Specifically, during the transition from round spermatids to elongated spermatids, the authors observed that the amplitude of translational activation may depend not only on ~60 nt poly(A) tails, as conventionally considered, but also on ~150 nt poly(A) tails.
To explore how poly(A) tails are elongated during spermiogenesis, the authors focused on TENT5C. While previous studies have shown that knockout (KO) of Tent5c is essential for male fertility in mice, the authors further demonstrated that a catalytically dead (dcat) TENT5C mutation also resulted in male infertility and absence of sperm heads in mature spermatozoa, which phenocopied Tent5c KO mice. Although Tent5c dcat mice exhibited a substantial change in the poly(A) tail length of Insl3 mRNA, the authors found that Insl3 may not be responsible for the observed phenotypes. Instead, they focused on Odf1, which showed a more modest change in poly(A) tail length but exhibited abnormal expression during late spermiogenesis in Tent5c dcat mice. As the spermatozoa of Tent5c dcat mice phenocopied those of Odf1 KO mice, the authors conclude that TENT5C-mediated regulation of Odf1 via its poly(A) tail is important for spermiogenesis and male fertility.
While the experimental design and the observation of poly(A) tail dynamics during the transition from round to elongated spermatids are informative and contribute to our understanding of mRNA translation during spermiogenesis, there are concerns regarding the mechanistic interpretation of TENT5C and ODF1. Specifically, compared to the previously reported shortening of immunoglobulin mRNAs in Tent5c dcat mice (Bilska et al., Nat Commun, 2020, 11:2032, PMID: 32341344), the poly(A) tail shortening observed in Odf1 was relatively modest, making it unclear whether the phenotypes can be explained by ODF1.

Major:
(1) The authors should validate the expression of the catalytically inactive TENT5C protein to confirm that the observed phenotypes are due to loss of catalytic activity rather than a reduction or absence of TENT5C protein itself.
(2) The authors should provide direct evidence supporting an interaction between TENT5C and Odf1 mRNA, and demonstrate TENT5C's poly(A) extension activity specifically on Odf1 mRNA.
(3) Previous studies (Zhang et al., Heliyon, 2024, 10:e38263, PMID: 39386799) reported that ODF1 protein was detected from step 8 spermatids onward, whereas the authors found ODF1 signals only after step 14-15. (stage III, Figure 5c). The axonemes are already formed in these steps, although the authors found abnormalities in the axoneme (Figure 5e). The authors should provide antibody validation data to support their immunohistochemistry results. Additionally, quantification of ODF1 protein levels in Tent5c dcat spermatids and spermatozoa would further substantiate their claims.
(4) The authors mixed the word usage of stages of seminiferous tubules and steps of spermatids, including but not limited to L29 and L551. Furthermore, while the authors state that 'ODF1 protein is not detected until spermatid stage (step) 16', Figure 5c clearly shows ODF1 expression beginning in stage III seminiferous tubules, which contain step 14-15 spermatids, but not step 16. These discrepancies should be corrected for clarity and accuracy.

Minor:
(5) L291-337: The data related to Insl3 should be moved to supplementary information rather than included in the main figures, to maintain focus on the central findings of the study.
(6) L507: spelling mistake of 'sterility'.

Reviewer #3

(Remarks to the Author)

The manuscript by Baptissart et al explores the role of TENT5C in mouse sprmiogenesis. A role for both poly(A) length regulation of the Odf1 transcript and the poly(A) polymerase TENT5c in spermiogenesis was previous known. Here, the authors explore the changes in poly(A) length across the entire transcriptome in differentiating spermatids. They unambiguously define the function of TENT5C poly(A) polymerase activity in relation to the dynamics of Odf1 poly(A) tail length. The failure of TENT5c to lengthen Odf1 transcript's poly(A) tail in spermatids is associated with aberrant translation and accumulation of the encoded protein.
Overall, I think the experiments and analyses support the conclusions drawn. In addition to an exciting and precise message, I think the datasets in the paper are of value to the entire field. I am supportive of publication but have a few concerns that would need to be addressed to give my full endorsement.

Major Points
How was the differential RNA between elongating and round spermatids measured? Was this a relative or absolute quantification? An explanation should be provided in the text.

There are many single cell spermatogenesis RNA-seq datasets published, can the principal observations on transcript stability form the bulk RNA-seq analysis be validated through analysis of the single cell datasets.

How was translational activation measured? A brief explanation in the text would be required. Is it from published data sets?

Minor Points

To make the manuscript more approachable to a wide audience, I would avoid the use of the RS, ES, P and D acronyms.

One introductory sentence for the technique Nanopolish would benefit the manuscript.

Maybe the category in 'others' in figure 1 could be renamed unchanged?

For the poly(A) length figures, I suggest removing the vertical lines or to use them across the entire axis.

Version 1:

Reviewer comments:

Reviewer #1

(Remarks to the Author)
The authors have responded to the reviewers' queries by performing additional experiments and by introducing extensive changes in the manuscript. The manuscript is improved and the authors' conclusions are better supported by the data.

It is suggested that the authors add a paragraph in the discussion to clarify how a TENT5C-mediated polyadenylation may function on top of the widespread tPAP mediated polyadenylation in round spermatids. Is the function of TENT5C to temporally extend and stabilize the poly(A) tail of ODF1 in elongating spermatids?

Reviewer #2

(Remarks to the Author)
The authors addressed most of my concerns. I have just some minor comments.

Line 33: step-10 round spermatids → step-10 elongating spermatids.
Lines 423-425: It would be informative to also present the RNA amount of other HTCAs
Figure 5b. In the image of the end piece, the fibrous sheath can be observed, indicating that this section is the principal piece rather than the end piece.
Figure 5c. It would be good if the labels for the red and yellow lines were included in the figure, like in the other figures.
Lines 483-484: The authors stated that 'these granules were found in the vicinity of Sertoli cells' while squashed testicular samples were used.

Open Access This Peer Review File is licensed under a Creative Commons Attribution 4.0 International License, which permits use, sharing, adaptation, distribution and reproduction in any medium or format, as long as you give appropriate credit to the original author(s) and the source, provide a link to the Creative Commons license, and indicate if changes were made.
In cases where reviewers are anonymous, credit should be given to 'Anonymous Referee' and the source.
The images or other third party material in this Peer Review File are included in the article's Creative Commons license, unless indicated otherwise in a credit line to the material. If material is not included in the article's Creative Commons license and your intended use is not permitted by statutory regulation or exceeds the permitted use, you will need to obtain permission directly from the copyright holder.
To view a copy of this license, visit https://creativecommons.org/licenses/by/4.0/

Reviewer #1 (Remarks to the Author)

Comment from reviewer:

With this report, Dr. Baptissart and collaborators have investigated the function of the non-canonical cytoplasmic TENTC poly(A) polymerase in the regulation of gene expression during spermatogenesis. The authors have used two genetic mouse models of inactivation of TENTC: a knockout of the TENTC gene that prevents the expression of the protein, and a D90N and D92N mutations in the TENTC catalytic site that disrupts its catalytic activity. Both mouse mutants have the same phenotype of male sterility and production of headless spermatozoa. The authors use Oxford Nanopore Technologies (ONT) to sequence single RNA molecules and to quantify the poly(A) length. They found that the Poly(A) tail length increases at the transition from pachytene to round spermatids and then decreases from round spermatids to elongating spermatids in an mRNA-specific fashion. The authors conclude that during spermatid elongation, transcripts with 150nt poly(A)are stable and are translated. The gradual shortening of the details detected in ES follows translation and eventually leads to destabilization and decay. They then show that TENTC inactivation causes a decrease in poly(A) tail length in a small subset of mRNAs, the most affected transcripts being INSL3 and Ptgds. Given the lack of correlation between INSL3 poly(A) tail length and protein accumulation during spermatogenesis, they focus on ODF1 mRNA whose poly(A) tail is moderately affected by the loss of TENT5C function. Immunofluorescence analysis shows that ODF1 protein is still translated on a TENT5C null background but that protein localization in elongating spermatids and spermatozoa is disrupted. The authors conclude that TENT5C is required for spatial translation of the ODF1 mRNA.

That TENTC plays a critical function in spermatogenesis has already been reported in several publications and linked to the control of poly(A) tail length. However, the present study has the advantage of using isolated spermatogenic cells at different stages to measure the poly(A) length, providing a more accurate picture of the Poly(A) dynamics during spermiogenesis. In addition, the use of two different genetic models to study the impact of TENT5 loss of function adds strength to the study. The conclusion that poly(A)

lengthening is associated with increased translation is a novel concept.

Answer to the comment:

We thank the reviewer for a thorough and insightful evaluation of the manuscript. Addressing their comments allowed us to substantially strengthen the study. We also appreciate the reviewer's positive remarks regarding the novelty of the results presented.

Comment from reviewer:

Fig. 1 By comparing poly(A) length and transcript levels, the authors establish a correlation between poly(A) length and stability of the mRNA. Can the authors better define the group of transcripts defined as "others" and provide some examples?

Answer to the comment:

We thank the reviewer for allowing us to clarify this point. We initially used the term "Others" to describe transcripts whose abundance did not show a statistically significant change between our conditions (adjusted p-value > 0.05). We agree that this might have caused confusion. We have now replaced this term with the more commonly used label "Unchanged" to better categorize these transcripts.

Comment from reviewer:

Line 301. The authors state that the poly(A) tails of Insl3 and Ptgds mRNAs are the most affected by the loss of TENT5C function. However, the authors do not mention whether this shortening of the poly(A) tail has any effect on transcript levels. Including this information would strengthen the conclusion that the length of the poly(A) tail correlates with mRNA stability.

Answer to the comment:

We thank the reviewer for this important comment highlighting a central aspect of our study.

Insl3 and *Ptgds* indeed show the strongest reduction in mean poly(A)-tail length in round and elongating spermatids (RS, ES) in the absence of TENT5C poly(A) polymerase activity (**Fig.4b and Extended Data Fig. 4b**). However, DESeq2 analysis of our direct RNA-seq data from purified RS and ES cells detected no significant differences in

transcript abundance between wild-type and *Tent5c* mutants (**Extended Data Table 1; lines 313-315 of the revised manuscript**).

The discrepancy between the poly(A) profile and transcript stability likely reflects a latency before decay becomes detectable, as well as sampling effects. In *Tent5c* mutants, *Insl3* and *Ptgds* shift from ~150-nt tails to an intermediate ~60-nt state (**Fig.4c and Extended Data Fig. 4c**). This change primarily affects translation; substantial decay typically follows only after further deadenylation to very short tails (<30 nt), which we expect in the most advanced stages of spermiogenesis. Because the purified RS/ES fractions contain spermatids across multiple differentiation stages, bulk direct RNA-sequencing may lack sensitivity to detect decreases in RNA abundance restricted to the final steps of spermatid differentiation. This interpretation is supported by two lines of evidence:

First, in Leydig cells, where RNA metabolism is at steady state, the shortening of *Insl3* poly(A) tails (e.g., ~180 to ~60 nt) is accompanied by reductions in both protein levels and transcript accumulation, consistent with decay progressing far enough to be detected by bulk RNA-seq (**Fig.4d and e, Extended Data Fig. 4h**).

Second, similar to *Insl3* and *Ptgds*, poly(A) shortening of *Odf1* in ES occurs without detectable changes in bulk RNA-seq (**Extended Data Table 1; lines 536-538 of the revised manuscript**); yet RNA FISH reveals a significant reduction in *Odf1* mRNA abundance restricted to the final stage of spermatid elongation, which coincides with the late phenotype of sperm decapitation (**Fig.6c**).

Comment from reviewer:

Line 368. The authors mention ODF1 and Smcp as transcripts affected by the loss of TENT5C function. What about poly (A) tail and translation of the other components of the ODF or components of the connective piece? Is the polyadenylation of these other transcripts not affected?

Answer to the comment:

We thank the reviewer for the opportunity to address this important question that is essential for understanding the origin of the phenotype.

In elongating spermatids (ES), only a few transcripts (69) showed a reduction in mean poly(A)-tail length and were therefore classified as putative TENT5C targets (**Fig. 4b**). Among them, *Odf1* stood out as the only transcript with direct functional relevance to the HTCA/ODF axis, consistent with the headless spermatozoa phenotype. We have now expanded the manuscript to explicitly clarify the status of other head–tail coupling apparatus (HTCA) and outer dense fiber (ODF)-associated transcripts and proteins.

Aside from *Odf1*, no other mRNAs encoding HTCA/ODF factors implicated in acephalic spermatozoa and male infertility showed reduced poly(A) tail length in *Tent5c*-mutant ES cells (**Extended Data Table 3; lines 416-425 of the revised manuscript**).

We further used mass spectrometry and immunostaining to comprehensively characterize the abundance and localization of ODF2, WDR64 and TSSK2, key components of the ODFs, the manchette and RNA processing granules, structures essential for late spermiogenesis and proper sperm morphogenesis. Consistent with the unchanged poly(A) tail profiles of their transcripts, the abundance and distribution of these key proteins were preserved in the absence of TENT5C-dependent mRNA polyadenylation (**Extended Data Table 4, Extended Data Fig. 5h, l, m; lines 507-525 of the revised manuscript**).

Together, these data indicate that ODF1 is the major structural protein affected by TENT5C deficiency, arguing against a broad disruption of HTCA/ODF-related structures.

<u>Comment from reviewer:</u>

Line 408. The authors note that the accumulation of the ODF1 protein may increase with the loss of TENT5C activity. This increase is in contrast with the observation that the loss of TENT5C function is associated with a moderate decrease in poly(A) length. A decrease in the length of the poly(A) tail should have affected the translation of the mRNA and the accumulation of the protein. Whether ODF1 protein is indeed increased in the TENT5C null is inferred from immunofluorescence measurements, which are difficult to evaluate even though the signal seems to be increased in the two mutants in Fig. 5C. Can ODF1 western blot be used to quantify the ODF1 protein in Wild type and

TENT5C null mutants? Possibly this could be done after isolation of spermatogenic stages where the protein accumulates. The authors should then discuss how an increased accumulation of the proteins relates to decreased poly(A) length.

Answer to the comment:

We would like to thank the reviewer for the opportunity to revisit our results using quantitative analysis.

To assess ODF1 accumulation quantitatively, we performed mass spectrometry on purified round spermatids (RS) and elongating spermatids (ES) from wild-type and *Tent5c* mutants. ODF1 protein abundance was not significantly different between *Tent5c*$^{wt/wt}$ and *Tent5c*$^{dcat/dcat}$ in either RS or ES extracts (**Extended Data Table 4; lines 492-494 of the revised manuscript**).

ODF1 accumulation and localization are highly dynamic during late spermatid differentiation (**Fig. 6a and Extended Data Fig. 5a**). Although quantitative, mass spectrometry on purified RS and ES has two main limitations. First, while our germ cell purification strategy separates RS from ES, each fraction includes spermatids spanning multiple differentiation steps, potentially masking step-specific changes (**Extended Data Fig. 1e**). Second, because we analyze only purified RS and ES, we do not capture ODF1 accumulating into residual bodies that are eliminated during spermiation (**Extended Data Fig. 5a**).

To gain higher temporal and spatial resolution, we performed immunofluorescence on seminiferous tubule squash preparations and testis sections. We combined these approaches with standardized high-resolution imaging and quantification to compare ODF1 signal intensities between *Tent5c* mutants and wild-type controls at individual steps of spermatid differentiation.

Although we initially noted a qualitative increase in ODF1 signal in stage III–VI tubules of TENT5C-deficient mice, this trend was only marginal and did not reach statistical significance upon quantification (**Extended Data Fig. 5a to d**). However, our new analyses revealed a robust and significant ODF1 reduction in two compartments of the mutant spermatids, specifically at step 16: (1) along the flagellar midpiece, with a pronounced loss at the HTCA where ODF1 normally accumulates to secure head-tail

linkage (quantified on squashed tubules; **Fig. 6a and b; lines 477-494 of the revised manuscript**), and (2) in cytoplasmic droplets, becoming almost undetectable in residual bodies (quantified on testis sections; **Extended Data Fig. 5a to d; lines 462-476 of the revised manuscript**).

Our revised conclusions now align with the expected effects of *Odf1* poly(A) tail shortening on RNA metabolism. TENT5C catalytic activity maintains *Odf1* transcripts with ~150-nt poly(A) tails optimal for translation. Loss of TENT5C poly(A) polymerase activity induces premature shortening of *Odf1* poly(A) tails; their translation decreases in parallel with ODF1 protein levels in late spermatids, compromising head–tail fastening.

<u>Comment from reviewer:</u>

Line 411 ODF1 appears also to be mis-localized in spermatozoa in Fig. 5d. However, the authors (see do not report data on poly(A) tail length above) and protein localization accumulation for the other components of the outer dense fiber or of the connective piece. This information is necessary to determine whether only ODF1 mis-localization is the cause of the phenotype. On what basis do the authors not consider the possibility that ODF1 protein interactions are disrupted?

Answer to the comment:

We appreciate the reviewer's emphasis on the value of an exhaustive analysis to better understand the molecular basis of the phenotype. We performed immunostaining for three ODF1-interacting proteins, components of key structures implicated in late spermatid differentiation.

ODF2 was used to assess outer dense fiber integrity (**Extended Data Fig. 5h to k**), WDR64 to track manchette formation (**Extended Data Fig. 5l**), and TSSK2 to examine the dynamics of chromatoid remnants and reticulated bodies, two major hubs of RNA processing during late spermiogenesis (**Extended Data Fig. 5m**). No mislocalization or reduction in ODF2, WDR64, or TSSK2 signal intensity was observed in spermatids of *Tent5c* mutant mice (**lines 507-525 of the revised manuscript**).

These data support a model in which loss of TENT5C activity specifically impacts ODF1 deposition. The preserved localization of multiple ODF1-interacting partners

across relevant subcellular structures rules out a broad impairment of ODF1-associated assemblies. However, we cannot exclude subtle alterations in specific ODF1 protein–protein interactions that are not resolved by these assays. We now comment this possibility in the Discussion (**lines 694-706 of the revised manuscript**).

Comment from reviewer:

A previous report that the authors cite (PMID: 38909026) concluded that Tent5 controls polyadenylation of mRNAs with a leader sequence, thus affecting secretion of specific proteins. Can the authors confirm this prediction? Some of the mRNAs they consider affected do not seem to contain a leader sequence.

Answer to the comment:

We welcome the opportunity to discuss an interesting aspect of TENT5C target specificity across cell types.

We replicated the analysis reported in Brouze et al., with the poly(A) tail profiles generated in our study [1]. Briefly, we used the annotation generated by the TargetP-2.0 server to identify among our poly(A) tail datasets the transcripts encoding signal peptides (SP). A hypergeometric test was used to determine SP enrichment among transcripts categorized according to the dynamics of their poly(A) tail in response to *Tent5c* mutations.

In elongating spermatids (ES), *Ptgds* and *Insl3* were among the nine SP-encoding transcripts showing shorter poly(A) tails in the absence of TENT5C activity; however, SP-encoding mRNAs were not significantly enriched among ES transcripts classified as putative TENT5C targets (**Rebuttal Figure 1**). We applied this same analysis to the poly(A) tail dataset from wild-type and *Tent5c*$^{dcat/dcat}$ testes enriched with interstitial cells. Unlike ES, this cell population shows a statistical overrepresentation of SP mRNAs among TENT5C putative targets (**Rebuttal Figure 1**, 3.28-fold positive enrichment compared to expectation; p-value=0.0005).

In their study, Brouze et al. searched for sequence features across a collection of samples including whole adult testis [1]. Here we refined their original analysis by showing that SP is a determining factor for TENT5C-mediated polyadenylation

specifically in testes enriched for Leydig cells. This result is consistent with the role of TENT5C in another secretory cell type. In plasma cells, TENT5C is associated with the endoplasmic reticulum where it selectively stabilizes mRNAs encoding (ER)-targeted proteins such as immunoglobulin [2].

Overall, these results show that TENT5C targets various physiological functions depending on cell type. In spermatids, TENT5C poly(A) polymerase activity includes, but is not restricted to, SP-encoding transcripts. Our study reveals a critical role for TENT5C in supporting spermatid head-tail fastening, with *Odf1* identified as a new TENT5C target.

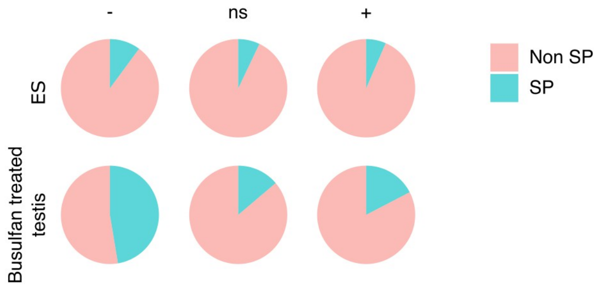

Rebuttal Figure 1: SP-encoding mRNAs are not significantly enriched among ES transcripts classified as putative TENT5C targets.

Pie charts showing the proportion of signal peptide–encoding (SP; blue) and non–signal peptide–encoding (Non-SP; red) mRNAs among transcripts from elongated spermatids (ES) or busulfan-treated testes. Transcripts are categorized according to the dynamic of their poly(A) tail length in response to Tent5c dcat mutations (-, shortening ; ns, non-significant; +, lengthening).

n=3 biological replicates per condition.

<u>Comment from reviewer:</u>

Line 556-557 Discussion. The authors conclude that ODF1 is mis-localized in the absence of TENT5C activity. However, this statement is difficult to evaluate with the data provided. Higher magnification data should be included. It is also not clear whether it is the ODF1 mRNA that is also mis-localized. Please discuss.

Answer to the comment:

The reviewer's comment is entirely justified, particularly considering the new insights provided by the set of higher-resolution immunostainings.

In the initial submission, we focused our interpretations on the ODF1 signal detected on testis sections; particularly on the apparent increase and more diffuse distribution of ODF1 staining in step-14/15 spermatids (stage III/IV) from *Tent5c* mutants. With the quantifications obtained, not only on testis sections, but also using tubule squashes, we can now better understand ODF1 protein signal intensity and localization. Our new conclusion highlights the significant decrease in ODF1 intensity in two cellular compartments in *Tent5c*-mutant mice: (1) A decrease in ODF1 intensity was measured along the midpiece of spermatids step-16 with a prominent drop at the HTCA where ODF1 aggregates in control mice (observed with squash; **Fig. 6a and b; lines 477-494 of the revised manuscript**); (2) ODF1 signal significantly drops in the cytoplasmic droplets of spermatids step-16, and almost disappears as the residual bodies get released to travel down to the basal membrane of the epithelium (observed with testis section and squash; **Extended Data Fig. 5a, Fig. 6a ; lines 462-494 of the revised manuscript**).

Poly(A) tail length is known to influence transcript stability and subcellular distribution [3]. As suggested by the reviewer, we examined the impact of TENT5C loss of function on *Odf1* transcript accumulation and localization using RNA FISH. As reported previously in rat [4], *Odf1* mRNA accumulates early in step 5-6 round spermatids and remains detectable through the final steps of spermiogenesis in wild-type mice (**Fig. 6c**). While broadly distributed throughout the cytoplasm for most of spermiogenesis, *Odf1* mRNAs cluster in a single aggregate near the nucleus of step-16 spermatids just before spermiation (**Fig. 6c**). In *Tent5c* mutants, overall *Odf1* mRNA levels and distribution are largely comparable to wild-type until immediately prior to spermiation, when in step-16 spermatids the *Odf1* mRNA aggregates fail to form and the signal is lost (**Fig. 6c**).

These additional quantitative immunostaining and RNA FISH analyses show that TENT5C activity is required for the late-stage, compartment-specific enrichment of *Odf1* mRNAs and ODF1 protein.

Comment from reviewer:

The statement that TENT5C interacts with the manchette and centrioles and PLK4 is intriguing (see PMID: 32433990), but no experimental data are reported to show that in ES, TENT5C protein and ODF1 mRNA colocalize in these structures. Should the authors provide data of colocalization of TENT5C with centrioles in spermatids? Isolation of centrioles and showing that TENT5 C and ODF mRNA are enriched in this fraction would support the authors' conclusions.

Answer to the comment:

We understand the reviewer's concern and agree that demonstrating enrichment of both TENT5C and *Odf1* mRNA in centriolar/HTCA-associated structures would strengthen evidence for a direct functional relationship.

In our study, single-molecule RNA FISH and high-resolution immunostaining show that *Odf1* mRNA and ODF1 protein increase during spermatid differentiation and, in step-16 spermatids, concentrate in discrete aggregates at the neck (**Fig. 6a to c**). Importantly, this localization depends on TENT5C polyadenylation: in *Tent5c*$^{dcat/dcat}$ step-16 spermatids, *Odf1* mRNA aggregates are lost, and ODF1 protein is significantly reduced along the flagellum, with the most pronounced decrease at the HTCA (**Fig. 6a to c**). Together, these data establish a direct requirement for TENT5C polyadenylation activity in maintaining *Odf1* mRNA aggregates and ODF1 protein accumulation at the HTCA of step-16 spermatids to support head–tail fastening.

Existing observations support coherent working models for how TENT5C and *Odf1* mRNAs may physically interact. TENT5C is enriched at the manchette of early ES and then falls below detection as *Odf1* mRNA granules appear (**Fig. 6c**) [5]. We propose that TENT5C-mediated polyadenylation stabilizes a pool of *Odf1* transcripts in the vicinity of the manchette during early spermatid elongation. As the manchette collapses at the neck of late spermatids, these stabilized mRNAs could persist and concentrate in granules; their local translation may promote ODF1 accumulation at the HTCA to secure head–tail fastening. The HTCA is a centriole-derived structure. Notably, TENT5C has been detected in centrosomal fractions from U2OS cells and reported to interact with the centriolar protein PLK4 [6,7]. If a similar interaction occurs in spermatids, a small

residual pool of TENT5C could remain associated with centriole-derived compartments at the HTCA, where it may further support *Odf1* mRNA stability and localized translation.

To provide direct evidence that TENT5C localizes to the HTCA with *Odf1* transcripts, we attempted to pull down TENT5C from testis extracts of *Tent5c-GFP*-tagged mice and used mass spectrometry or qPCR to detect associated proteins and transcripts. Despite several immunoprecipitation conditions (including previously published protocols) [8], TENT5C immunoprecipitation remains unsuccessful, likely due to low protein abundance and/or weak epitope accessibility. Such limitations would be expected if indeed TENT5C is embedded within a highly organized, stage-restricted structure such as the manchette or the HTCA.

We have added a clarifying statement to the Discussion outlining these mechanistic models, while explicitly noting that direct interaction of TENT5C with *Odf1* mRNA remains to be established (**lines 655-673 of the revised manuscript**).

Comment from reviewer:

The authors correctly state the genetic model used indicates that the catalytic activity of TENT5C is required during spermatogenesis. However, can the authors make predictions on the molecular complexes affected or not in the dcat/dcat mutant? All the interactions of TENT5C involving domains outside of the catalytic domain should be preserved in this genetic model. Would this imply that targeting requires the catalytic domain only?

Answer to the comment:

The dcat model we used carries two point mutations (D90N and D92N) within the catalytic nucleotidyltransferase (NTase) domain of TENT5C [8]. Because these substitutions specifically target TENT5C enzymatic activity, we anticipate that protein–protein interactions mediated by regions outside the catalytic domain should remain intact. Consistently, *in vitro* analyses indicate that the dcat mutations do not compromise TENT5C protein stability or subcellular localization but selectively abolish TENT5C poly(A) polymerase activity (**Rebuttal Figure 2**); moreover, molecular

dynamics simulations support that the dcat substitutions do not destabilize TENT5C structure (**Rebuttal Figure 3**).

Importantly, TENT5C does not contain an intrinsic RNA-binding domain. Therefore, its association with target RNAs is mediated through interacting proteins, such as poly(A)-binding proteins and other RNA-binding factors within a larger ribonucleoprotein complex. Under this model, it is reasonable to propose that in *Tent5c*$^{dcat/dcat}$ mutants, these complexes may still assemble and engage targeted RNAs but would be functionally inactive and therefore unable to extend poly(A) tails.

<u>Comment from reviewer:</u>

Minor points

Line 395-397 The authors state that Prm2 poly(A) tail is not changed in the Tent5c dcat/dcat. Then they state that "Consistently, no change TNP2 protein expression were observed in the TENT5C -depleted spermatids." Should the authors show that TNP2 mRNA poly(A) tail is not changed in their genetic model?

Answer to the comment:

We recognize the source of confusion and have clarified it in the revised manuscript.

We included *Prm2* to illustrate the *Odf1*-specific sensitivity to loss of TENT5C poly(A) polymerase activity, as *Odf1* and *Prm2* share similar poly(A)-tail profiles in elongating spermatids (**Fig. 5c**). However, the impact of *Tent5c* mutation on PRM2 protein accumulation has not been studied. We will revise the text to avoid implying that it has. In contrast, TNP2 protein levels were shown to be unchanged in TENT5C-depleted spermatids [5]. Consistent with this observation, *Tnp2* mRNA also shows no detectable change in poly(A)-tail profile in response to the *Tent5c* mutation. However, *Tnp2* poly(A) tail profile differs from that of *Odf1* and therefore is not the most informative control for arguing *Odf1* specificity (**Fig. 2c**).

We clarified this point in the revised manuscript and adjusted the wording to ensure the *Prm2* and *Tnp2* examples are not conflated (**lines 438-440 of the revised manuscript**).

Comment from reviewer:

Line 499 “For example, Prm2 transcripts encode for the last of the transition proteins and protamines to appear in late spermatids.” This sentence is confusing

Answer to the comment:

We rephrased this paragraph for clarification (**lines 597-601 of the revised manuscript**): “For example, Prm2 transcripts encode for the last protamine to be expressed in late spermatids [46]. As such, Prm2 transcripts primarily accumulate with poly(A) tails ~150-nt long in ES. In contrast, Prm1 mRNAs show a reduced proportion of ~150-nt tails and an accumulation of ~60-nt tails indicating earlier translational events.”

Reviewer #2 (Remarks to the Author)

Comment from reviewer:

In this study, Baptissart et al. examined the temporal dynamics of mRNA poly(A) tail length during spermatogenesis by performing direct RNA sequencing on FACS-sorted spermatogenic cells. Specifically, during the transition from round spermatids to elongated spermatids, the authors observed that the amplitude of translational activation may depend not only on ~60 nt poly(A) tails, as conventionally considered, but also on ~150 nt poly(A) tails.

To explore how poly(A) tails are elongated during spermiogenesis, the authors focused on TENT5C. While previous studies have shown that knockout (KO) of Tent5c is essential for male fertility in mice, the authors further demonstrated that a catalytically dead (dcat) TENT5C mutation also resulted in male infertility and absence of sperm heads in mature spermatozoa, which phenocopied Tent5c KO mice. Although Tent5c dcat mice exhibited a substantial change in the poly(A) tail length of Insl3 mRNA, the authors found that Insl3 may not be responsible for the observed phenotypes. Instead, they focused on Odf1, which showed a more modest change in poly(A) tail length but exhibited abnormal expression during late spermiogenesis in Tent5c dcat mice. As the spermatozoa of Tent5c dcat mice phenocopied those of Odf1 KO mice, the authors conclude that TENT5C-mediated regulation of Odf1 via its poly(A) tail is important for spermiogenesis and male fertility.

While the experimental design and the observation of poly(A) tail dynamics during the transition from round to elongated spermatids are informative and contribute to our understanding of mRNA translation during spermiogenesis, there are concerns regarding the mechanistic interpretation of TENT5C and ODF1. Specifically, compared to the previously reported shortening of immunoglobulin mRNAs in Tent5c dcat mice (Bilska et al., Nat Commun, 2020, 11:2032, PMID: 32341344), the poly(A) tail shortening observed in Odf1 was relatively modest, making it unclear whether the phenotypes can be explained by ODF1.

Answer to the comment:

We thank the reviewer for their careful evaluation of the manuscript and understand the concern that *Odf1* poly(A) tail shortening appears modest in the absence of TENT5C activity.

We now show that this modest shift has mechanistically relevant consequences for *Odf1* mRNA metabolism. Using single-molecule RNA FISH and high-resolution immunofluorescence, we detected a significant decrease in *Odf1* mRNA and ODF1 protein accumulation specifically at the latest step of spermatid differentiation and predominantly at the neck/HTCA, a compartment critical for head–tail attachment (**Fig. 6 a-c**). Because these defects are localized and stage-specific, they are diluted in bulk measurements and were not detected in purified spermatid population analyses (mass spectrometry and DESeq2 analysis of direct RNA sequencing; **Extended Data Table 1 and 4**). Likewise, if premature *Odf1* poly(A) tail shortening occurs mainly at the latest steps of spermiogenesis, only a subset of *Odf1* transcripts would shift toward shorter tails in bulk elongated spermatids, explaining the modest average decrease in *Tent5c*$^{dcat/dcat}$ cells (**Fig. 5c**).

Together, these technical considerations reconcile the modest reduction in *Odf1* poly(A)-tail length observed from bulk RNA sequencing with a specific reduction in *Odf1* transcript and ODF1 protein at the neck of step-16 spermatids, supporting a mechanistic link between TENT5C-dependent polyadenylation and the head–tail attachment defects associated with infertility.

More broadly, we demonstrate that RNA poly(A)-tail profiling can reveal discrete regulatory events that underlie infertility phenotypes when other bulk methods fail to resolve effects confined to a narrow differentiation window.

<u>Comment from reviewer:</u>

Major:

(1) The authors should validate the expression of the catalytically inactive TENT5C protein to confirm that the observed phenotypes are due to loss of catalytic activity rather than a reduction or absence of TENT5C protein itself.

Answer to the comment:

The reviewer raises a key point regarding our model, which we clarify below. The *Tent5c*$^{dcat/dcat}$ mice were previously described, and the stability of the catalytically inactive TENT5C protein was not addressed in the original study [8]. Because no suitable TENT5C antibody is available, we attempted to assess TENT5C accumulation in wild-type and *Tent5c*$^{dcat/dcat}$ mice by mass spectrometry (**Extended Data Table 4**). However, TENT5C levels in both whole testes and purified elongating spermatids (ES) were below the detection limit in both genotypes.

In the absence of more sensitive approaches, we therefore used a synthetic overexpression system in cultured cells to evaluate the intrinsic stability of the catalytically deficient TENT5C protein. We generated expression vectors encoding FHpHG-tagged mouse TENT5C wild-type (WT) and a catalytically inactive version (DCAT; D90A/D92A) analogous to the mutations introduced in the *Tent5c*$^{dcat/dcat}$ mouse model [8]. Twenty-four hours after transfection into HEK293T cells, TENT5C WT and TENT5C DCAT were detected by GFP fluorescence and displayed similar subcellular localization (**Rebuttal Figure 2a**). Moreover, TENT5C WT and DCAT accumulated to comparable levels, as assessed by Western blotting against the HA tag (**Rebuttal Figure 2b**). Together, these results indicate that the *Tent5c*^{dcat} mutations are not expected to alter TENT5C subcellular distribution or intrinsic stability. We also extracted RNA from the corresponding cells to confirm loss of enzymatic activity. Overexpression of TENT5C WT led to a global increase in poly(A)-tail length across the transcriptome

relative to cells transfected with the empty vector (**Rebuttal Figure 2c**). In contrast, expression of TENT5C DCAT did not increase global poly(A)tail length (**Rebuttal Figure 2c**).

Overall, these data support the conclusion that the key difference between TENT5C WT and TENT5C DCAT is functional: the dcat mutations are unlikely to compromise protein stability or localization *in vivo*, but instead specifically abolish TENT5C poly(A) polymerase activity.

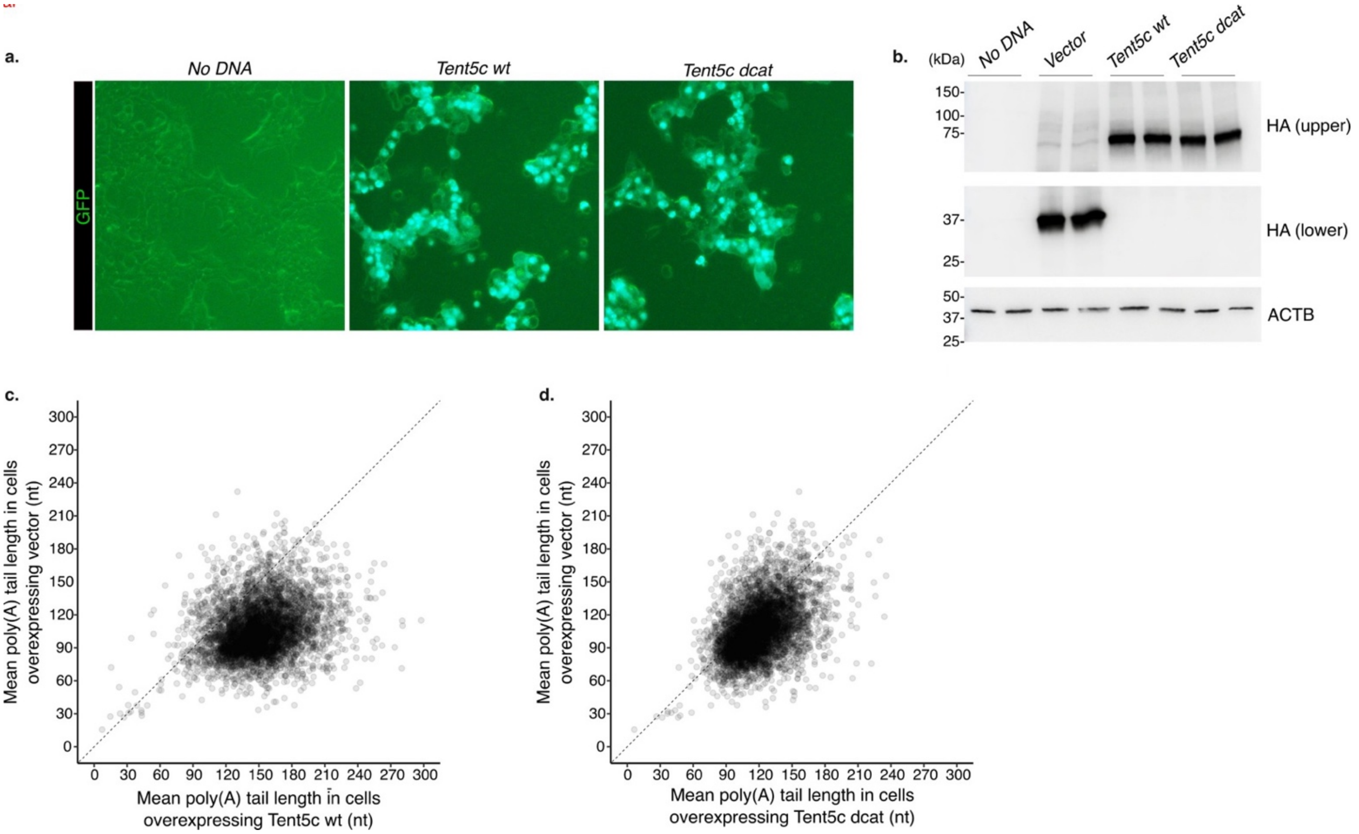

Rebuttal Figure 2: dcat mutations affect TENT5C poly(A) polymerase activity while preserving protein localization and stability *in vitro*.

(a) Representative micrographs of green fluorescence in HEK293T cells 24 hours after transfection with expression vectors encoding FHpHG-tagged mouse TENT5C wild-type (*Tent5c wt*) and a catalytically inactive version of TENT5C (*Tent5c dcat*; D90A/D92A). Cells transfected without DNA are used as control.

(b) Representative immunoblot showing HA and ACTIN B protein accumulation in HEK293T 24 hours after transfection with expression vectors encoding FHpHG-tagged

mouse TENT5C wild-type (*Tent5c wt*) and a catalytically inactive version of TENT5C (*Tent5c dcat*; D90A/D92A). Cells transfected without DNA (No DNA) or with an empty vector (Vector) are used as control.

(c) Scatter plot comparing the mean poly(A) tail length of each transcript in HEK293T cells 24 hours after transfection with an empty vector or *Tent5c wt*. Each dot represents an individual transcript.

(d) Scatter plot as in (c) after transfection with an empty vector or *Tent5c dcat***.**

To confirm that the *Tent5c*^{dcat} mutations (D90N/D92N) do not alter the overall protein structure relative to the WT, we performed molecular dynamics simulations, running three independent 1-*μ*s trajectories per system. The DCAT model was generated by introducing the D90N and D92N substitutions into the solvated WT structure reported previously [9].

Across all trajectories, the backbone RMSD relative to the equilibrated starting conformation remained low and comparable between WT and DCAT, indicating no large-scale structural drift over the microsecond timescale (**Rebuttal Figure 3a**). Residue-level flexibility, assessed by backbone RMSF, showed similar profiles in both systems: higher fluctuations were confined to expected mobile regions (loops and termini), while low RMSF values corresponded to the stable structured core (**Rebuttal Figure 3b**). Finally, dynamic cross-correlation matrices (DCCMs), computed from Cα positions and averaged over each 1-*μ*s trajectory across three runs per system, showed nearly identical patterns for WT and DCAT, consistent with similar coordinated motions (**Rebuttal Figure 3c and d**).

Together, RMSD, RMSF, and DCCM analyses indicate that the DCAT substitutions do not compromise TENT5C structural stability and that the mutant displays dynamics comparable to the WT protein over the simulated microsecond timescale.

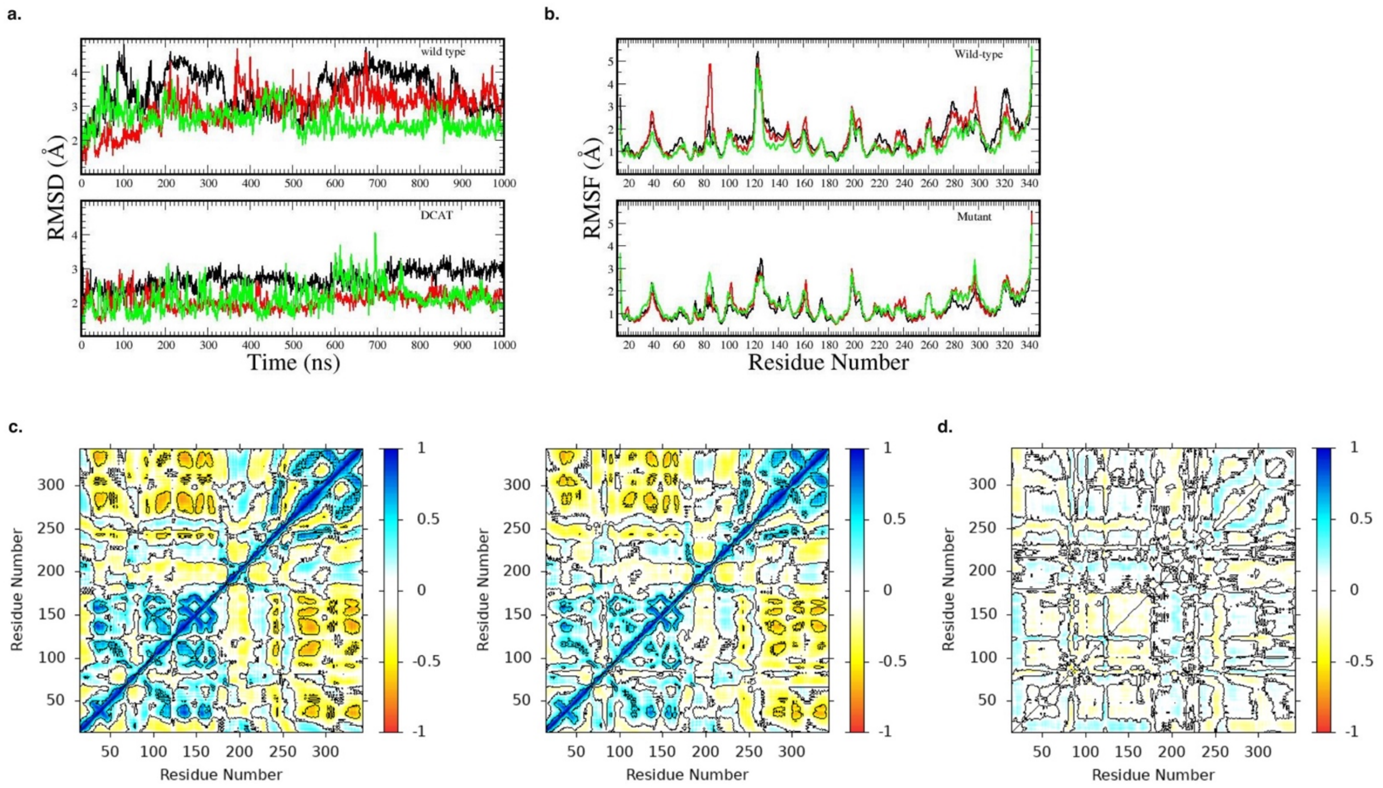

Rebuttal Figure 3: DCAT substitutions do not compromise TENT5C structural stability.
(a) Root mean squared deviations (RMSD) plot of TENT5C WT and DCAT (D90N/D92N). RMSD were calculated by averaging distances between the backbone atoms over 1-microsecond. n= 3 independent runs (black, red, and green).
(b) Root mean squared fluctuations (RMSF) plot of the backbone atoms of TENT5C WT and_DCAT (D90N/D92N). RMSF values were calculated by averaging displacements of individual residues from their mean positions over 1-microsecond. n= 3 independent runs (black, red, and green).
(c) Dynamic cross-correlation matrices (DCCM) showing residue–residue correlated\ motions over 1-microsecond for TENT5C WT (left) and dcat (right; D90N/D92N). n= 3 independent runs.
(d) Differential dynamic cross-correlation matrices (DCCM) obtained by subtracting the WT DCCM from the dcat (D90N/D92N) DCCM shown in (c). n= 3 independent runs.

<u>Comment from reviewer:</u>

(2) The authors should provide direct evidence supporting an interaction between TENT5C and Odf1 mRNA, and demonstrate TENT5C's poly(A) extension activity specifically on Odf1 mRNA.

Answer to the comment:

We agree that demonstrating direct, transcript-specific activity of TENT5C on *Odf1*, including evidence of physical association, would strengthen the case for a central role of *Odf1* in the phenotype.

We discussed multiple lines of indirect evidence supporting a physical association between TENT5C and *Odf1* mRNA in our response to Reviewer 1 (**see lines 266–301 of this rebuttal letter**).

In this answer, we focus on whether TENT5C specifically extends the poly(A) tail of *Odf1* mRNA. We tested this using an *in vitro* reporter assay. Because poly(A)-tail processing is 3′UTR-dependent, we cloned the *Odf1* and *Insl3* 3′UTRs (putative TENT5C targets in ES cells) downstream of a luciferase reporter, using the *Odf2* 3′UTR as a negative control. In HEK293T cells, co-expression of these reporters with TENT5C WT, TENT5C DCAT (D90A/D92A), or control vector showed that all constructs displayed similar poly(A)-tail lengthening upon TENT5C WT overexpression (**Rebuttal Figure 4**). This effect was absent with TENT5C DCAT, confirming dependence on TENT5C poly(A) polymerase activity, but it did not reveal specificity for the *Odf1* or *Insl3* 3′UTRs (**Rebuttal Figure 4**). We interpret this as a limitation of non-physiological TENT5C overexpression in a heterologous system, likely outside its native RNP context. We therefore favor our *in vivo* genetic model to assess TENT5C target specificity.

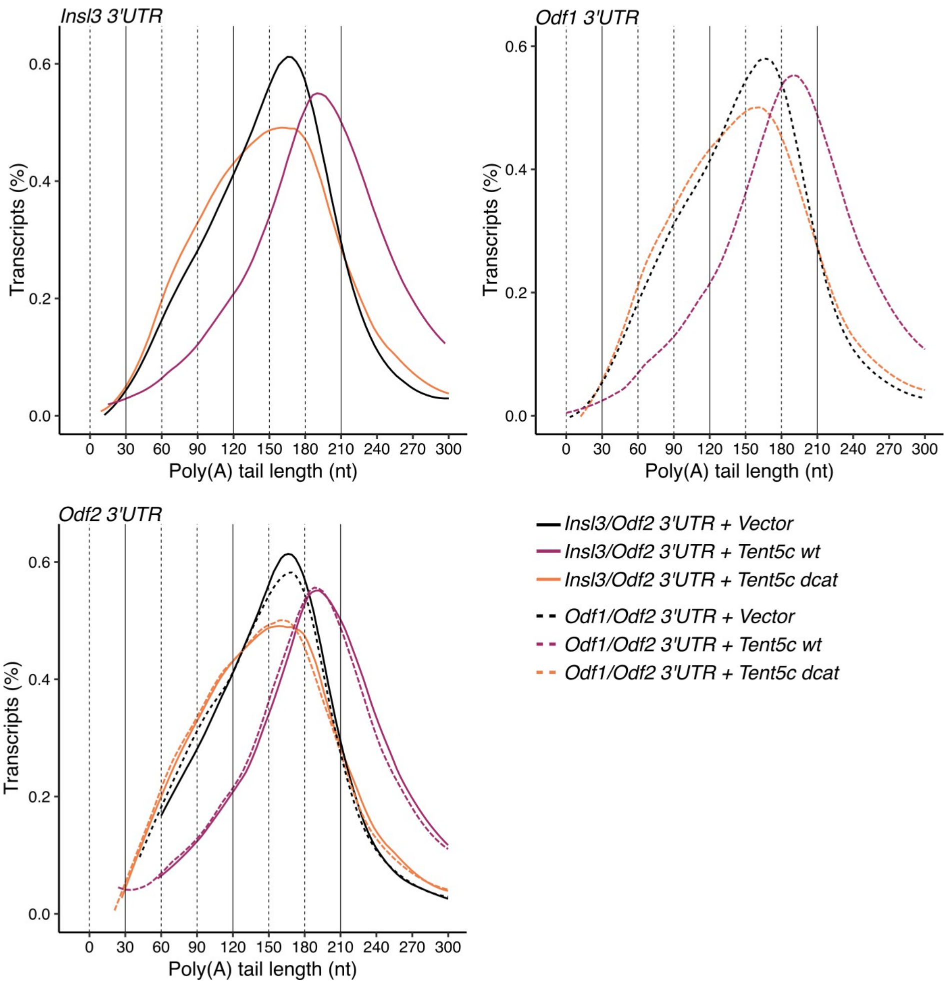

Rebuttal Figure 4: TENT5C-dependent polyadenylation extends the poly(A) tails of *Odf1*-, *Insl3*-, and *Odf2*-3′UTR reporter transcripts in HEK293T cells.

(a) Poly(A) tail length density plot for luciferase reporters bearing the 3′UTR of Odf1 or Insl3 (putative TENT5C targets in elongating spermatids) or the 3′UTR of Odf2 (negative control). Reporters were co-transfected into HEK293T cells together with expression vectors encoding TENT5C WT, catalytically inactive TENT5C DCAT (D90A/D92A), or empty vector. n=1 biological replicate per condition. The local polynomial regression fitting is shown as a solid line for each condition. nt: nucleotide.

We combined a mouse model of TENT5C catalytic inactivation with the purification of ES, setting up a rigorous framework to define physiological TENT5C targets in highly differentiated cells, a cellular context that cannot be recapitulated *in vitro*.

Our data show that the impact of TENT5C catalytic inactivation on ES poly(A)-tail profiles is highly selective: *Odf1* is among the only 69 transcripts that exhibited a measurable reduction in poly(A)-tail length and were therefore classified as putative TENT5C targets (**Fig. 4b**).

Note that this degree of selectivity is consistent with a prior report showing that TENT5C influences a limited number of transcripts in whole testis [1]. *Odf1* stood out as one of only four targets conserved across datasets supporting its identification as a particularly robust candidate (**Fig. 5a**) [1].

Moreover, *Odf1* emerged as the most compelling functional candidate; the *Tent5c* mutant phenotype overlaps with key features of ODF1 loss-of-function, including headless spermatozoa and the presence of flagellar defects (**Fig. 3c and 5b**) [10]. To test whether other components of the head–tail coupling apparatus (HTCA) and outer dense fibers (ODFs) might also be affected, we relied on an exhaustive list of known, predicted, and candidate HTCA/ODF factors implicated in head–tail linkage and male fertility [11], including *Ccdc42*, *Spag4*, *Odf2*, and *Wdr64*. Notably, none of these transcripts showed reduced poly(A)-tail length in *Tent5c* mutant ES cells; within this HTCA/ODF-focused set, *Odf1* was the only transcript that emerged as a TENT5C-dependent polyadenylation target, consistent with a specific link to the head–tail coupling phenotype (**Extended Data Table 3; lines 416-425 of the revised manuscript**).

Together, these data support selective, *in vivo* TENT5C-dependent poly(A) regulation of *Odf1*.

<u>Comment from reviewer:</u>

(3) Previous studies (Zhang et al., Heliyon, 2024, 10:e38263, PMID: 39386799) reported that ODF1 protein was detected from step 8 spermatids onward, whereas the authors found ODF1 signals only after step 14-15. (stage III, Figure 5c). The axonemes are already formed in these steps, although the authors found abnormalities in the axoneme (Figure 5e). The authors should provide antibody validation data to support

their immunohistochemistry results. Additionally, quantification of ODF1 protein levels in Tent5c dcat spermatids and spermatozoa would further substantiate their claims.

Answer to the comment:

We agree that quantitative assessment of ODF1 levels is important, and we now provide quantification of ODF1 accumulation using multiple complementary approaches, as described in our response to Reviewer 1 (**see lines 120–154 of this rebuttal letter**).

We acknowledge the discrepancy in the reported timing of ODF1 protein detection. In our study, we used the same ODF1 antibody as the previous report (Abcam ab197029) [12]. However, our staging strategy differed: rather than relying primarily on nuclear morphology, we combined DAPI with PNA staining, which enables more accurate assignment of spermiogenesis steps. We also assessed ODF1 accumulation using two independent approaches: 1- testis sections, which provide high staging resolution across the full germ-cell population of the epithelium (**Extended Data Fig. 5a to c, Fig. 6c**), and 2- seminiferous tubule squashes, which allow detection of ODF1 in subcellular structures that can be difficult to resolve in sections (**Fig. 6a and b**). Both approaches showed a consistent window of ODF1 accumulation during spermiogenesis: ODF1 was first detected in the cytoplasm and along the proximal axoneme at step 14 (stages II/III), followed by prominent accumulation at the head–tail coupling apparatus (HTCA) by step 16 (stage VII/VIII).

The late accumulation of ODF1 protein coincides with the marked increase in diameter of the ODF's during late spermiogenesis (step 15 to 17 in rats) [13]. In mouse, although the axoneme is largely assembled by steps 15–16, periaxonemal accessory structures continue to mature: the ODFs and the fibrous sheath are assembled around the axoneme [14,15], the annulus reaches its final position and mitochondria organize into the mature midpiece sheath [16]. Consistent with this late maturation window, the abnormalities we observed in *Tent5c*$^{dcat/dcat}$ or *Odf1*-deficient mice (mitochondrial disorganization, misaligned ODFs, and fibrous sheath protrusions) point to defects in late periaxonemal assembly and support a critical role for ODF1 during advanced spermiogenesis.

Our staging of ODF1 accumulation is further validated by the stage-appropriate distributions of additional periaxonemal maturation markers. Specifically:

- ODF2: initial signal in the cytoplasm/proximal axoneme at step 9 (stages IX/X), strongest at step 15 (stages IV/VI), and markedly reduced by step 16 (stage VII/VIII), consistent with prior observations (**Extended Data Fig. 5h**) [17].
- WDR64: detectable posterior to the nucleus and along the manchette from steps 8–14, and decreases after manchette disassembly (steps 15–16) (**Extended Data Fig. 5l**) [18].
- TSSK2: appears as a small granule in step-9 spermatids, enlarges near the neck through steps 13-14, then relocates along the tail to the annulus by step 15; the granule largely disappears by step 16, with residual signal near the acrosome (**Extended Data Fig. 5m**) [19].

The dynamics of ODF1 protein accumulation are also consistent with its RNA metabolism. First, *Odf1* mRNA recruitment to polysomes has been reported during the maturation phase of spermiogenesis, typically corresponding in mouse to step 13-16 spermatids [4]. In our data, the *Odf1* poly(A) profile exhibits an emerging ~60-nt poly(A)-tail peak in elongated spermatids, which is consistent with late translational activation (**Fig. 2c**). This pattern resembles that of *Prm2*, whose protein is detected no earlier than steps 14–16 (**Fig. 2c**) [20]. By contrast, the *Odf2* poly(A) profile shows a stronger ~60-nt peak, consistent with the earlier onset of ODF2 protein detection (~step 9) relative to ODF1 (~step 14) (**Rebuttal Fig. 5, Extended Data Fig. 5h and Fig. 6a**). In addition, *Odf1* mRNA accumulates in discrete aggregates in step-16 spermatids, coinciding with peak ODF1 enrichment at the HTCA at this stage (**Fig. 6a to c**).

Overall, these complementary protein- and RNA-based measurements converge on a late, stage-specific onset of ODF1 expression, supporting our staging of ODF1 accumulation during spermiogenesis.

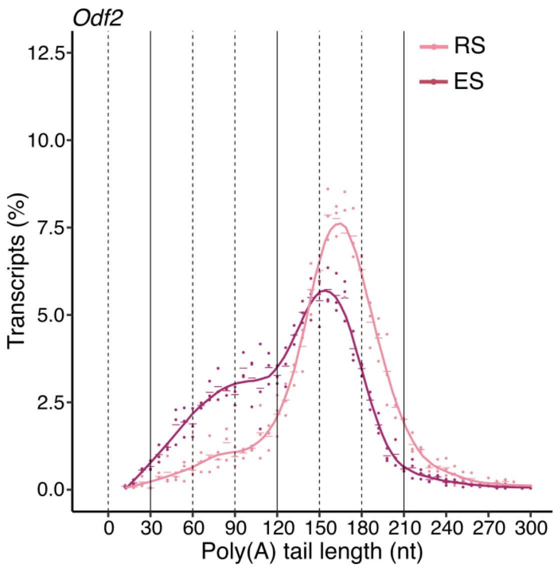

Rebuttal Figure 5: *Odf2* poly(A)-tail distribution suggests early translation during spermiogenesis.

(a) Poly(A) tail length density plot for *Odf2* transcripts in round spermatid (RS, pink) and elongated spermatid (ES, magenta) transcriptomes. Dots indicate values for individual biological replicates. The bars indicate the relative mean percentage of transcripts for each poly(A) tail length. The local polynomial regression fitting is shown as a solid line for each condition. nt: nucleotide.

Comment from reviewer:

(4) The authors mixed the word usage of stages of seminiferous tubules and steps of spermatids, including but not limited to L29 and L551. Furthermore, while the authors state that ‘ODF1 protein is not detected until spermatid stage (step) 16’, Figure 5c clearly shows ODF1 expression beginning in stage III seminiferous tubules, which contain step 14-15 spermatids, but not step 16. These discrepancies should be corrected for clarity and accuracy.

Answer to the comment:

Thank you for pointing out these mistakes. We have updated the manuscript accordingly.

Comment from reviewer:

Minor:

(5) L291-337: The data related to Insl3 should be moved to supplementary information rather than included in the main figures, to maintain focus on the central findings of the study.

Answer to the comment:

We agree that the data related to *Insl3* and *Ptgds* do not contribute to the central finding of the study. However, because *Insl3* is the most prominent TENT5C target identified in our dataset, we believe it provides valuable context for the broader impact of TENT5C-dependent polyadenylation. For this reason, we have retained the *Insl3* results in the main figures (**Fig. 4b**). However, in line with the reviewer's suggestion, we moved the *Ptgds*-related data to the **Extended Data Fig. 4**.

<u>Comment from reviewer:</u>

(6) L507: spelling mistake of 'sterility'.

Answer to the comment:

We apologize for this mistake and have corrected it.

Reviewer #3 (Remarks to the Author):

<u>Comment from reviewer:</u>

The manuscript by Baptissart et al explores the role of TENT5C in mouse sprmiogenesis. A role for both poly(A) length regulation of the Odf1 transcript and the poly(A) polymerase TENT5c in spermiogenesis was previous known. Here, the authors explore the changes in poly(A) length across the entire transcriptome in differentiating spermatids. They unambiguously define the function of TENT5C poly(A) polymerase activity in relation to the dynamics of Odf1 poly(A) tail length. The failure of TENT5c to lengthen Odf1 transcript's poly(A) tail in spermatids is associated with aberrant translation and accumulation of the encoded protein.

Overall, I think the experiments and analyses support the conclusions drawn. In addition to an exciting and precise message, I think the datasets in the paper are of value to the

entire field. I am supportive of publication but have a few concerns that would need to be addressed to give my full endorsement.

Answer to the comment:

We thank the reviewer for their evaluation of our manuscript. We appreciate their support for the main conclusions and for highlighting the value of the datasets generated. Their insightful comments have allowed us to further refine our conclusions.

Comment from reviewer:

Major Points

How was the differential RNA between elongating and round spermatids measured? Was this a relative or absolute quantification? An explanation should be provided in the text.

Answer to the comment:

We used DESeq2 on our direct RNA sequencing dataset to quantify relative changes in RNA accumulation between round spermatids (RS) and elongating spermatids (ES). Because transcription is largely silenced during late spermiogenesis, we interpret RS-to-ES differences in RNA abundance as primarily reflecting relative changes in RNA stability. For better clarity, we detailed the methodology in the results section (**lines 118-121 of the revised manuscript**) and methods (**lines 1278-1288 of the revised manuscript**).

Comment from reviewer:

There are many single cell spermatogenesis RNA-seq datasets published, can the principal observations on transcript stability form the bulk RNA-seq analysis be validated through analysis of the single cell datasets.

Answer to the comment:

We thank the reviewer for this interesting suggestion.

Because their normalization assumes comparable RNA composition across conditions, standard bulk RNA-seq methods provide relative rather than absolute changes in transcript abundance [21]. In our system, we expect a global reduction in RNA

content in ES compared with RS [22,23]. Under these circumstances, DESeq2's median-of-ratios normalization can partially "normalize away" a global decrease in RNA, thereby underestimating the magnitude of downregulation. In contrast, single-cell RNA-seq–based comparisons performed within defined cell states can better capture global shifts in absolute transcript abundance.

To test whether this bias applies in our dataset, our purified RS and ES transcriptomes were mapped to cell clusters from a whole testis single-cell RNA sequencing dataset to computationally predict the cell type composition (**Extended Data Fig. 1e**) [24]. Our RS population mapped predominantly to the SC2 RS1 cluster, whereas our ES fraction most closely matched the CS ES cluster (**Extended Data Fig. 1e**). We then performed absolute differential RNA accumulation analysis between SC2 RS1 and CS ES within the same single-cell dataset using Seurat and compared the cumulative fold-change distributions from the single-cell analysis to those obtained from bulk DESeq2 (**Extended Data Table 2)** [25].

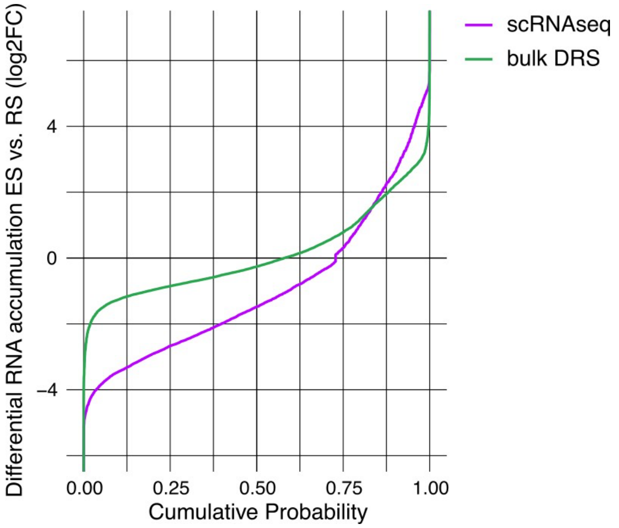

Rebuttal Figure 6: Bulk direct RNA sequencing underestimates the number and magnitude of downregulated transcripts.

Cumulative distribution curves of fold changes in RNA abundance between elongated spermatids (ES) and round spermatids (RS). Single-cell results (purple) are compared to those obtained from bulk DESeq2 (green).

As anticipated, bulk DESeq2 analysis attenuated the global downshift in ES (**Rebuttal Figure 6**): the number and magnitude of downregulated transcripts were underestimated, whereas the upregulated (stable) population was broadly consistent between the two approaches.

We replicated the correlation analysis between poly(A)-tail length and RNA stability, this time inferred from single-cell differential expression. This independent approach confirms our original DESeq2-based conclusions (**Extended Data Fig. 1i to k, lines 135-139 of the revised manuscript**).

Comment from reviewer:

How was translational activation measured? A brief explanation in the text would be required. Is it from published data sets?

Answer to the comment:

We are happy to clarify this approach.

We reanalyzed a published mouse testis dataset (E-MTAB-7247) [26]. For RS and ES, translation efficiency (TE) was inferred by integrating matched Ribo-seq and RNA-seq read counts. Differential ribosome occupancy (i.e., differential translational activity) between the two cell types was determined using Xtail v1.1.5 [27].

We added a brief clarification in the Results section (**lines 193-196 of the revised manuscript**). Methodological details are provided in the Methods section (**lines 1297-1300 of the revised manuscript**).

Minor Points

Comment from reviewer:

To make the manuscript more approachable to a wide audience, I would avoid the use of the RS, ES, P and D acronyms.

Answer to the comment:

We considered the suggestion by the reviewer, but after deliberation we decided to retain the acronyms for readability.

Comment from reviewer:

One introductory sentence for the technique Nanopolish would benefit the manuscript.

Answer to the comment:

We thank the reviewer for the suggestion. Nanopolish estimates poly(A)-tail length per read from Oxford Nanopore direct RNA sequencing using the raw electrical signal (FAST5), rather than counting A's after basecalling, which is often unreliable in homopolymeric regions. Specifically, it converts the signal span corresponding to the poly(A) segment into a nucleotide-length estimate using an internal model [28,29]. We are now providing methodological details in the Methods section (**lines 1262-1264 of the revised manuscript**).

Comment from reviewer:

Maybe the category in 'others' in figure 1 could be renamed unchanged?

Answer to the comment:

We appreciate the suggestion. We have now replaced the term "others" with the more commonly used label "unchanged" to better categorize these transcripts. See also our response to Reviewer 1 (**lines 40–44 of this rebuttal letter**).

Comment from reviewer:

For the poly(A) length figures, I suggest removing the vertical lines or to use them across the entire axis.

Answer to the comment:

The vertical lines are intended to indicate poly(A)-tail length ranges associated with distinct RNA metabolic outcomes, and we find them helpful for guiding interpretation of the distributions. Specifically, the bold lines delineate the poly(A)-tail length windows we quantified. Methodological details are provided in the Methods section (**lines 1264-1277 of the revised manuscript**). We reviewed the figures to ensure that these markers are displayed consistently across panels.

1. Brouze, M. et al. TENT5-mediated polyadenylation of mRNAs encoding secreted proteins is essential for gametogenesis in mice. Nat Commun 15, 5331 (2024). https://doi.org/10.1038/s41467-024-49479-4
2. Herrero, A. B. et al. FAM46C controls antibody production by the polyadenylation of immunoglobulin mRNAs and inhibits cell migration in multiple myeloma. J Cell Mol Med 24, 4171-4182 (2020). https://doi.org/10.1111/jcmm.15078
3. Groisman, I. et al. CPEB, maskin, and cyclin B1 mRNA at the mitotic apparatus: implications for local translational control of cell division. Cell 103, 435-447 (2000). https://doi.org/10.1016/s0092-8674(00)00135-5
4. Burmester, S. & Hoyer-Fender, S. Transcription and translation of the outer dense fiber gene (Odf1) during spermiogenesis in the rat. A study by in situ analyses and polysome fractionation. Mol Reprod Dev 45, 10-20 (1996). https://doi.org/10.1002/(SICI)1098-2795(199609)45:1<10::AID-MRD2>3.0.CO;2-V
5. Zheng, C. et al. Non-canonical RNA polyadenylation polymerase FAM46C is essential for fastening sperm head and flagellum in mice. Biol Reprod 100, 1673-1685 (2019). https://doi.org/10.1093/biolre/ioz083
6. Kazazian, K. et al. FAM46C/TENT5C functions as a tumor suppressor through inhibition of Plk4 activity. Commun Biol 3, 448 (2020). https://doi.org/10.1038/s42003-020-01161-3
7. Chen, H., Lu, D., Shang, G., Gao, G. & Zhang, X. Structural and Functional Analyses of the FAM46C/Plk4 Complex. Structure 28, 910-921 e914 (2020). https://doi.org/10.1016/j.str.2020.04.023
8. Bilska, A. et al. Immunoglobulin expression and the humoral immune response is regulated by the non-canonical poly(A) polymerase TENT5C. Nat Commun 11, 2032 (2020). https://doi.org/10.1038/s41467-020-15835-3
9. Li, Y. et al. TENT5C functions as a corepressor in the ligand-bound glucocorticoid receptor and estrogen receptor α complexes. FEBS J 292, 5168-5184 (2025). https://doi.org/10.1111/febs.70137

10 Yang, K. et al. The small heat shock protein ODF1/HSPB10 is essential for tight linkage of sperm head to tail and male fertility in mice. Mol Cell Biol 32, 216-225 (2012). https://doi.org/10.1128/MCB.06158-11

11 Buglak, D. B., Galletta, B. J. & Rusan, N. M. Molecular models of the sperm head-tail coupling apparatus. J Cell Sci 138 (2025). https://doi.org/10.1242/jcs.264168

12 Zhang, Y. et al. WDR64, a testis-specific protein, is involved in the manchette and flagellum formation by interacting with ODF1. Heliyon 10, e38263 (2024). https://doi.org/10.1016/j.heliyon.2024.e38263

13 Oko, R. & Clermont, Y. Light microscopic immunocytochemical study of fibrous sheath and outer dense fiber formation in the rat spermatid. Anat Rec 225, 46-55 (1989). https://doi.org/10.1002/ar.1092250108

14 Lehti, M. S., Kotaja, N. & Sironen, A. KIF3A is essential for sperm tail formation and manchette function. Mol Cell Endocrinol 377, 44-55 (2013). https://doi.org/10.1016/j.mce.2013.06.030

15 Clermont, Y., Oko, R. & Hermo, L. Immunocytochemical localization of proteins utilized in the formation of outer dense fibers and fibrous sheath in rat spermatids: an electron microscope study. Anat Rec 227, 447-457 (1990). https://doi.org/10.1002/ar.1092270408

16 Guan, J., Kinoshita, M. & Yuan, L. Spatiotemporal association of DNAJB13 with the annulus during mouse sperm flagellum development. BMC Dev Biol 9, 23 (2009). https://doi.org/10.1186/1471-213X-9-23

17 Schalles, U., Shao, X., van der Hoorn, F. A. & Oko, R. Developmental expression of the 84-kDa ODF sperm protein: localization to both the cortex and medulla of outer dense fibers and to the connecting piece. Dev Biol 199, 250-260 (1998). https://doi.org/10.1006/dbio.1998.8931

18 Hu, W. et al. CAMSAP1 role in orchestrating structure and dynamics of manchette microtubule minus-ends impacts male fertility during spermiogenesis. Proc Natl Acad Sci U S A 120, e2313787120 (2023). https://doi.org/10.1073/pnas.2313787120

19 Lehti, M. S. et al. The late chromatoid body component TSSK2 is involved in translational regulation in elongating spermatids in mice. Reproduction 170 (2025). https://doi.org/10.1530/REP-25-0297

20 Kim, C. R. et al. PHF7 Modulates BRDT Stability and Histone-to-Protamine Exchange during Spermiogenesis. Cell Rep 32, 107950 (2020). https://doi.org/10.1016/j.celrep.2020.107950

21 Love, M. I., Huber, W. & Anders, S. Moderated estimation of fold change and dispersion for RNA-seq data with DESeq2. Genome Biol 15, 550 (2014). https://doi.org/10.1186/s13059-014-0550-8

22 Kierszenbaum, A. L. & Tres, L. L. Structural and transcriptional features of the mouse spermatid genome. J Cell Biol 65, 258-270 (1975). https://doi.org/10.1083/jcb.65.2.258

23 Monesi, V. Ribonucleic Acid Synthesis during Mitosis and Meiosis in the Mouse Testis. J Cell Biol 22, 521-532 (1964). https://doi.org/10.1083/jcb.22.3.521

24 Lukassen, S., Bosch, E., Ekici, A. B. & Winterpacht, A. Characterization of germ cell differentiation in the male mouse through single-cell RNA sequencing. Sci Rep 8, 6521 (2018). https://doi.org/10.1038/s41598-018-24725-0

25 Stuart, T. et al. Comprehensive Integration of Single-Cell Data. Cell 177, 1888-1902.e1821 (2019). https://doi.org/10.1016/j.cell.2019.05.031

26 Wang, Z. Y. et al. Transcriptome and translatome co-evolution in mammals. Nature 588, 642-647 (2020). https://doi.org/10.1038/s41586-020-2899-z

27 Xiao, Z., Zou, Q., Liu, Y. & Yang, X. Genome-wide assessment of differential translations with ribosome profiling data. Nat Commun 7, 11194 (2016). https://doi.org/10.1038/ncomms11194

28 Loman, N. J., Quick, J. & Simpson, J. T. A complete bacterial genome assembled de novo using only nanopore sequencing data. Nat Methods 12, 733-735 (2015). https://doi.org/10.1038/nmeth.3444

29 Simpson, J. T. et al. Detecting DNA cytosine methylation using nanopore sequencing. Nat Methods 14, 407-410 (2017). https://doi.org/10.1038/nmeth.4184

Reviewer #1 (Remarks to the Author):

The authors have responded to the reviewers' queries by performing additional experiments and by introducing extensive changes in the manuscript. The manuscript is improved and the authors' conclusions are better supported by the data.

It is suggested that the authors add a paragraph in the discussion to clarify how a TENT5C-mediated polyadenylation may function on top of the widespread tPAP mediated polyadenylation in round spermatids. Is the function of TENT5C to temporally extend and stabilize the poly(A) tail of ODF1 in elongating spermatids?

We thank the reviewer for this suggestion. We modified a paragraph in the Discussion to clarify how TENT5C-mediated polyadenylation may function in relation to the broader polyadenylation landscape in round spermatids. Specifically, we now distinguish the widespread establishment of ~150-nt poly(A) tails in RS, which is unlikely to be driven by TENT5C, from a later and more selective role of TENT5C in elongating spermatids.

Reviewer #2 (Remarks to the Author):

The authors addressed most of my concerns. I have just some minor comments.

Line 33: step-10 round spermatids → step-10 elongating spermatids.

We thank the reviewer for highlighting this error. The text has been corrected from “step-10 round spermatids” to “step-10 elongating spermatids”.

Lines 423-425: It would be informative to also present the RNA amount of other HTCAs

We updated Extended Data Table 3 to include the DESeq2 results for HTCA-associated transcripts. These data are now available in the revised supplementary table.

Figure 5b. In the image of the end piece, the fibrous sheath can be observed, indicating that this section is the principal piece rather than the end piece.

We thank the reviewer for this observation. Figure 5b has been updated to include representative cross sections of the midpiece, principal piece, and end piece from wild-type and mutant mice.

Figure 5c. It would be good if the labels for the red and yellow lines were included in the figure, like in the other figures.

We thank the reviewer for this helpful suggestion. We have revised Figure 5c to include labels for the red and yellow lines, consistent with the presentation used in the other figures.

Lines 483-484: The authors stated that ‘these granules were found in the vicinity of Sertoli cells’ while squashed testicular samples were used.

The tubule squash preparation was designed to preserve, as much as possible, the relative proximity of cells within the seminiferous epithelium. Individual seminiferous tubules (approximately 3 cm segments) were laid out linearly on slides, and gentle pressure was applied to the coverslip to release cells while limiting disruption of their local organization.

DAPI, PNA, and SYCP3 staining enabled identification of germ-cell differentiation stages. In each region of interest, the combination of germ-cell types was consistent with the cellular associations expected for a given stage of the spermatogenic cycle. The sequential progression of spermatogenic stages was also maintained along the tubule axis. Spermatids (PNA-positive), meiotic cells (SYCP3-positive), and Sertoli cells (identified by their two DAPI-bright chromocenters) remained regionally enriched rather than randomly dispersed, consistent with partial preservation of the radial organization of the seminiferous epithelium.

Therefore, although squash preparations do not fully preserve the native architecture of the seminiferous epithelium, they retain sufficient local organization to support stage assignment and interpretation of the germ-cell environment. In response to the reviewer's comment, we revised "found in the vicinity of Sertoli cells" to "observed in regions enriched for Sertoli cells" to provide a more cautious interpretation.
